# Calculation of the relative metastabilities of proteins in subcellular compartments of *Saccharomyces cerevisiae*

**DOI:** 10.1186/1752-0509-3-75

**Published:** 2009-07-18

**Authors:** Jeffrey M Dick

**Affiliations:** 1 Department of Earth and Planetary Science, University of California, 307 McCone Hall, #4767 Berkeley, CA 94720-4767, USA; 2 Current address : School of Earth and Space Exploration, Arizona State University, P.O. Box 871404, Tempe, AZ 85287-1404, USA

## Abstract

**Background:**

Protein subcellular localization and differences in oxidation state between subcellular compartments are two well-studied features of the the cellular organization of *S. cerevisiae *(yeast). Theories about the origin of subcellular organization are assisted by computational models that can integrate data from observations of compositional and chemical properties of the system.

**Presentation and implications of the hypothesis:**

I adopt the hypothesis that the state of yeast subcellular organization is in a local energy minimum. This hypothesis implies that equilibrium thermodynamic models can yield predictions about the interdependence between populations of proteins and their subcellular chemical environments.

**Testing the hypothesis:**

Three types of tests are proposed. First, there should be correlations between modeled and observed oxidation states for different compartments. Second, there should be a correspondence between the energy requirements of protein formation and the order the appearance of organelles during cellular development. Third, there should be correlations between the predicted and observed relative abundances of interacting proteins within compartments.

**Results:**

The relative metastability fields of subcellular homologs of glutaredoxin and thioredoxin indicate a trend from less to more oxidizing as mitochondrion – cytoplasm – nucleus. Representing the overall amino acid compositions of proteins in 23 different compartments each with a single reference model protein suggests that the formation reactions for proteins in the vacuole (in relatively oxidizing conditions), ER and early Golgi (in relatively reducing conditions) are relatively highly favored, while that for the microtubule is the most costly. The relative abundances of model proteins for each compartment inferred from experimental data were found in some cases to correlate with the predicted abundances, and both positive and negative correlations were found for some assemblages of proteins in known complexes.

**Conclusion:**

The results of these calculations and tests suggest that a tendency toward a metastable energy minimum could underlie some organizational links between the the chemical thermodynamic properties of proteins and subcellular chemical environments. Future models of this kind will benefit from consideration of additional thermodynamic variables together with more detailed subcellular observations.

## Background

A complex interplay of chemical and biological forces is responsible for subcellular structure. There exist in eukaryotic cells gradients between subcellular compartments of chemical properties such as pH, oxidation-reduction (or redox) state and chemical activity of water, among others [1-5]. Different population of proteins are localized within each subcellular compartment [6-8]. Within compartments, the relative abundances or levels of different proteins are not equal [9], and different proteins predominate in the various subcellular populations depending on growth state of the cell and exposure to environmental stress [10]. Physical separation of key enzymes is thought to be essential in the cytoskeletal network and in regulation of metabolic pathways and other cellular functions [11,12]. The patterns of subcellular structure persist even though populations of proteins turnover through continual degradation and synthesis in cells [13].

The biosynthesis and transport of proteins in an energy-demanding process [14]. If cells have evolved to minimize their energy expenditure in the maintenance of biological function, it may be reasonable to expect to find signals of energy minimization in cellular organization. One such example is the finding that the relative abundances of amino acids in proteins correlate inversely with the metabolic cost of amino acid synthesis [15,16], and that this is a temperature-dependent function [17]. This observation is consistent with the notion that not all proteins are equal in energetic terms. In thermodynamic calculations of chemical affinity [18], the energy demands of protein formation (including synthesis and transport) are also a function of the local physical chemical environment, which includes variables such as oxidation-reduction potential [19]. It follows that subcellular structures that are characterized by differences in the amino acid composition of proteins and in chemical potentials have distinct energetic consequences.

For the purposes of this study, the hypothesis is made that cellular organization is in a local energy minimum. Energy minimization in biological operations is not a new hypothesis, especially in the context of fitness and adaptation to the environment [20-22]. However, the implications of this hypothesis for subcellular organization have not been investigated from the standpoint of equilibrium chemical thermodynamics. Algorithms for computing the requisite standard molal Gibbs energies of proteins [23] and the relative abundances of proteins in metastable equilibrium [19] have recently been reported. The goal of this study is to perform these types of calculations for model systems representative of various levels of subcellular organization and to compare the results with observations and measurements reported in the literature. If successful, this exercise may lead to an enhanced awareness about the chemical forces that shape cellular structure.

The theoretical approach adopted here is based on the description of a chemical system in terms of intensive variables. These variables include temperature, pressure and the chemical potentials of the system. It is convenient to denote the chemical potentials by the chemical activities or fugacities of basis species, for example the activity of H^+ ^(which defines pH) or the fugacity of oxygen. This permits comparison of the parameters of the model with reference systems described in experimental and other theoretical biochemical studies. In the following calculations, temperature and pressure were set to 25°C and 1 bar, respectively, and the logarithm of oxygen fugacity is the primary variable of interest. Below, oxidation-reduction potential and oxygen fugacity are used synonymously, and redox refers specifically to Eh. The oxidation-reduction potential of a system can be expressed in terms of Eh using an equation given in the Methods.

*S. cerevisiae *was chosen as a model system for the current investigation because there is abundant information about the subcellular distribution of proteins as well as independent measurements of the pH and oxidation state of some subcellular compartments. Also, the cellular development of yeast is extensively documented, which can yield other comparisons for some of the results of the model calculations.

There are two major parts to this paper. In the first part, the reactions corresponding to intercompartmental interactions between subcellular homologs (or isoforms) of particular enzymes and between reference model proteins for different compartments are quantified by calculating the oxygen fugacities for equal chemical activities of the reacting proteins in metastable equilibrium. The relative metastabilities of the reference model proteins are compared with some observations from the literature about reaction progress during the cell cycle. Specific known interactions between compartments are considered in order to derive values of the oxygen fugacity within compartments that best metastabilize the proteins contained within them. In the second part of this paper, the relative abundances of model proteins in metastable equilibrium are calculated and compared with measured abundances. The range of protein abundances in a metastable equilibrium population often approaches that seen in experiments over a narrow window of oxygen fugacity. Positive and negative correlations between the calculated and experimental relative abundances are found in some cases. The paper concludes with a summary of the findings and other implications of the hypothesis.

## Presentation of the hypothesis

Part of a cell's expenditure of metabolic fuel is directed toward the formation of proteins, including their synthesis and transport to other compartments. Even when it is normalized to the lengths of the proteins, the energy required for protein formation is not a constant, but depends on the composition and environment of the protein. If these energy differences are quantified, the relative abundances of model proteins in metastable equilibrium can be calculated. The compositions of these metastable assemblages depend on local environmental variables such as oxygen fugacity, which is a scale for oxidation-reduction potential in a system. The major hypothesis adopted for this investigation is that energy minimization is a force contributing to the organization of cells; this implies the possibility of an evolutionary convergence between biomolecular composition and the chemical properties of subcellular compartments. In a first set of tests of this hypothesis, chemical reactions among model proteins in known intercompartmental interactions were used to obtain values of oxygen fugacity for subcellular compartments that can be compared with measured redox values. A second set of calculations presented here shows that the relative abundances of proteins within compartments and of those that form complexes can be correlated in some cases with metastable equilibrium assemblages. These results provide theoretical constraints on the spontaneous generation of order in the distributions of proteins within cells and imply that work done by maintaining oxidation-reduction gradients can selectively alter the degrees of formation of assemblages of proteins.

## Testing the hypothesis

### Relative metastabilities of subcellular homologs of redoxins

Yeast cells have cytoplasmic, nuclear and mitochondrial homologs of glutaredoxin [24-26] and cytoplasmic and mitochondrial homologs of thioredoxin and thioredoxin reductase [27,28]. The names and chemical formulas of these proteins are listed in Table 1, together with some computed properties. The average nominal oxidation state of carbon () is a function of the relative proportions of the elements in the chemical formula (see Methods). In Table 1 the proteins with the lowest values of  are the mitochondrial homologs and those with the highest values of  are the nuclear homologs. Accordingly, the formation of the mitochondrial and nuclear proteins are energetically favored by relatively reducing and oxidizing conditions, respectively.

**Table 1:** Subcellular isoforms of glutaredoxin, thioredoxin and thioredoxin reductase in yeast^a^.

Protein	SWISS-PROT	Location	Length	Formula		*Z*	
		Glutaredoxin					
GLRX1	P25373	Cytoplasm	110	C_549_H_886_N_146_O_170_S_4_	-4565	-5.8	-0.182
GLRX2	P17695	Mitochondrion	143	C_715_H_1161_N_181_O_213_S_5_	-5617	0.1	-0.255
GLRX3	Q03835	Nucleus	285	C_1444_H_2195_N_371_O_463_S_10_	-12031	-24.5	-0.094
GLRX4	P32642	Nucleus	244	C_1226_H_1910_N_316_O_389_S_6_	-10276	-17.8	-0.140
GLRX5	Q02784	Mitochondrion	150	C_762_H_1200_N_196_O_227_S_6_	-5841	-6.1	-0.192
							
		Thioredoxin					
TRX1	P22217	Cytoplasm	102	C_502_H_785_N_123_O_150_S_5_	-3969	-3.1	-0.211
TRX2	P22803	Cytoplasm	103	C_497_H_780_N_122_O_153_S_5_	-4056	-3.1	-0.197
TRXB1	P29509	Cytoplasm	318	C_1509_H_2412_N_402_O_471_S_12_	-12330	-4.7	-0.159
TRX3	P25372	Mitochondrion	127	C_651_H_1049_N_167_O_181_S_10_	-4617	4.9	-0.255
TRXB2	P38816	Mitochondrion	342	C_1640_H_2615_N_449_O_501_S_14_	-12841	-1.5	-0.145

**a**. Amino acid compositions of subcellular isoforms of glutaredoxin (GLRX), thioredoxin (TRX) and thioredoxin reductase (TRXB) in *S. cerevisiae *were taken from the SWISS-PROT database [64] (accession numbers shown in the table). Chemical formulas of nonionized proteins, and calculated standard molal Gibbs energy of formation from the elements (, in kcal mol^-1 ^at 25°C and 1 bar) and net ionization state (*Z*) at pH = 7 of charged proteins are listed. Average nominal oxidation state of carbon () was calculated using Eqn. (6).

To quantify the metastability limits of the proteins in terms of the chemical environment, one can assess the energetics of formation reactions for the proteins. At metastable equilibrium, the predominant protein in a population is the one with the highest chemical activity (in this communication, activity refers to chemical activity rather than enzymatic activity; activity is equivalent to concentration for ideal systems, where activity coefficients are unity). This statement implies that the overall formation reaction from basis species (see Methods) for the predominant protein has a lower Gibbs energy (or higher chemical affinity) than any of the others. The consequences of these relationships can be portrayed on chemical activity diagrams using a previously described procedure that is encoded in the CHNOSZ software package [19], which was used to perform the calculations reported below (see Methods). Additional File 1 includes the program script and data files that were used to carry out these calculations and generate the tables and figures. So that the results described in this section can be reconstructed at pH = 7, the standard molal Gibbs energies () and net charges of ionized proteins at this pH are listed in Table 1.

**Figure 1:**
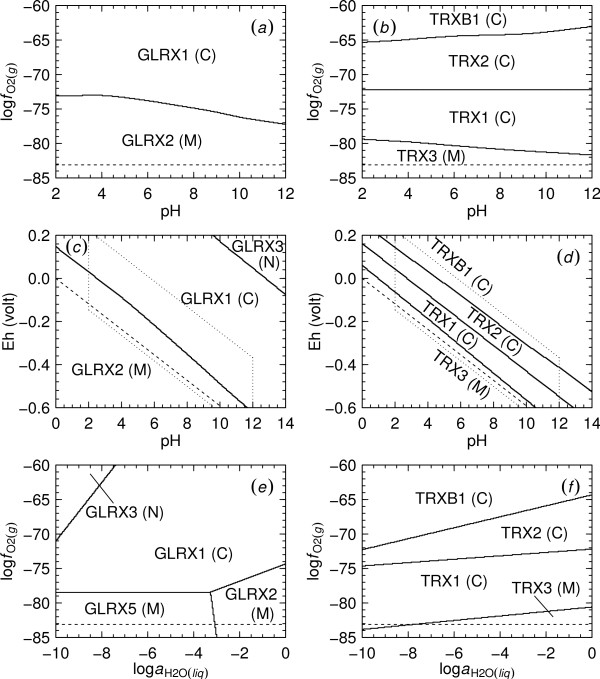
**Relative metastabilities of homologs of glutaredoxin and thioredoxin/thioredoxin reductase**. Predominance diagrams were generated for subcellular isoforms of (*a*, *c*, *e*) glutaredoxin (GLRX) and of (*b*, *d*, *f*) thioredoxin (TRX) and thioredoxin reductase (TRXB) in *S. cerevisiae*. The letters in parentheses following the labels indicate the subcellular compartment to which the protein is localized (C – cytoplasm; M – mitochondrion; N – nucleus). Calculations were performed for ionized proteins at 25°C and 1 bar and for reference activities of basis species noted in the Methods. Reduction stability limits of H_2_O are shown by dashed lines; the dotted lines in (*c*) and (*d*) correspond to the plot limits of (*a*) and (*b*).

In Figs. 1a and 1b the metastable equilibrium predominance limits of ionized proteins in the glutaredoxin and thioredoxin/thioredoxin reductase model systems are shown as a function of the logarithm of oxygen fugacity and pH. The computation of the relative metastabilities of the proteins included all five model proteins in the glutaredoxin system as candidates, but note in Fig. 1a that only two of the five proteins appear on the diagram. Those that do not appear are less metastable, or have greater energy requirements for their formation over the range of conditions represented in Fig. 1a than either of the proteins appearing in the figure.

The equal-activity lines in these pH diagrams are curved because the ionization states of the proteins depend on pH. The observation apparent in Fig. 1a that increasing log  favors formation of the cytoplasmic protein homolog relative to its mitochondrial counterpart is also true for the thioredoxin/thioredoxin reductase system shown in Fig. 1b. In comparing Figs. 1a and 1b note that in the latter figure, predominance fields for a greater number of candidate proteins appear, and that the predominance field boundary between mitochondrial and cytoplasmic proteins occurs at a lower oxidation-reduction potential. The dashed lines shown in each diagram of Fig. 1 are reference lines denoting the reduction stability limit of H_2_O (log  ≈ -83.1 at 25°C and 1 bar [29]).

Predominance diagrams as a function of Eh and pH for the glutaredoxin and thioredoxin/thioredoxin reductase systems are shown in Figs. 1c and 1d. Like log , Eh and pH together are a measure of the oxidation-reduction potential of the system; the different scales can be converted using Eqn. (5) in the Methods. The trapezoidal areas bounded by dotted lines in Figs. 1c and 1d show the ranges of Eh and pH corresponding to the limits of the log -pH diagrams of Figs. 1a and 1b. It can be deduced from these diagrams that if the upper log  limit of Fig. 1a were extended upward, this diagram would include a portion of the predominance field for the nuclear protein GLRX3.

It appears from Figs. 1a–b that increasing increasing log  at constant pH, or increasing pH at constant oxidation-reduction potential have similar consequences for the relative metastabilities of the cytoplasmic and mitochondrial homologs. In this analysis, however, pH does not appear to be a very descriptive variable; the magnitude of the effect of changing oxygen fugacity over several log units is greater than the effect of changing pH by several units. In further calculations described below pH was set to 7.

In Figs. 1e and 1f the logarithm of activity of water (log ) appears as a variable. In Fig. 1e it can be seen that the formation of a nuclear homolog of glutaredoxin is favored relative to the cytoplasmic homologs by decreasing activity of water and/or increasing oxygen fugacity, and that increasing relative metastabilities of the mitochondrial proteins are consistent with lower oxidation-reduction potentials. In Fig. 1f it appears that the formation of the thioredoxin reductase relative to thioredoxin is favored by increasing , and that for the thioredoxin the relative metastabilities of the mitochondrial proteins increase with decreasing .

### Comparison with subcellular redox measurements

Let us compare the positions of the predominance fields in Fig. 1 with measured subcellular redox states. The values of Eh derived from the concentrations of oxidized and reduced glutathione (GSSG and GSH, respectively) [2,30-32] and fluorescent probes [33] in extra- and subcellular environments reported in various studies were converted to corresponding values of log  using Eqn. (5) in the Methods and are listed in Table 2. In order to fill in the table as completely as possible, it was necessary to consider measurements performed on eukaryotic cells other than *S. cerevisiae *(e.g., HeLa [34] and mouse hybridoma [35] cells). The values of pH required for conversion of Eh to log  were also retrieved from the literature [36-38]. The computation of log  from Eh was performed at 25°C and 1 bar and with log  = 0. No measurements of vacuolar Eh were found, but it has been noted that Fe^+3 ^predominates over Fe^+2 ^in this compartment [39]. Hence, a nominal (and relatively very oxidizing) value of Eh for the vacuole was calculated that corresponds to equal activities of Fe^+3 ^and Fe^+2^.

**Table 2:** Nominal electrochemical characteristics of subcellular environments in eukaryotes. Values refer to yeast cells unless noted otherwise.

Environment	Eh, volt	pH	log ^m^
Extracellular (intestine)	-0.137 to -0.80^a^	3^g^	-83.3 to -79.4
Cytoplasm	-0.235 to -0.222^b^	6.5^h^	-75.9 to -75.0
Nucleus	-^c^	7.7^i^	-^c^
Mitochondrion	-0.360^d^	8^j^	-78.3
Endoplasmic reticulum	-0.185 to -0.133^e^	7.2^k^	-69.7 to -66.2
Vacuole	> +0.769^f^	6.2^l^	> -9.2

**a**. [30] (*Homo sapiens*). **b**. The lower and upper values are taken from [32] and [31], respectively. **c**. The state of the GSSG/GSH couple in the nucleus is thought to be more reduced than in the cytoplasm [40] (see text). **d**. [33] (*Homo sapiens *HeLa [34] cells). **e**. [2] (*Mus musculus*: mouse hybridoma cells [35]). **f**. Calculated by combining the law of mass action for Fe^+3 ^+ e^- ^⇌ Fe^+2 ^using standard molal Gibbs energies taken from Ref. [65] with . **g**. [36] (Homo sapiens). **h**. [37] (yeast). **i**. [66] (organism unspecified). **j**. [38] (HeLa) **k**. [67]. **l**. [1]. **m**. Values of Eh and pH listed here were combined with Eqn. (5) at *T *= 25°C, *P *= 1 bar and  = 1 to generate the values of log .

The current understanding of the major trends of redox states in compartments of eukaryotic cells can be summarized as, from most reducing to most oxidizing, mitochondrion – nucleus – cytoplasm – endoplasmic reticulum (ER) – extracellular [40]. Strong redox gradients within the mitochondrion are essential to its function [41], which is not captured by the single values listed in Table 2. Comparison nevertheless with the computational results shown in Fig. 1 indicates that a relatively reducing environment does favor the mitochondrial homologs over the others shown in the diagram.

Measurements of GSH/GSSG concentrations point to a lower redox state in the nucleus than in the cytoplasm, but the present model has the nuclear proteins favored by relatively oxidizing conditions. Studies using nuclear magnetic resonance (NMR) showing that the hydration state of the nucleus is higher than the cytoplasm [42,3] also seem to contradict the trend in Fig. 1e that the formation of the nuclear proteins is favored relative to their cytoplasmic counterparts by decreasing activity of water. Finally, mitochondrial pH is somewhat higher than that of the cytoplasm [37,38], but in Figs. 1a and 1b it appears that the predicted energetic constraints favor the cytoplasmic proteins at higher pHs. These comparisons indicate that the investigated metastable equilibrium constraints are not entirely responsible for the spatial distribution of the isoforms of redoxins in the cell.

### Relative metastabilities of reference model proteins

**Figure 2:**
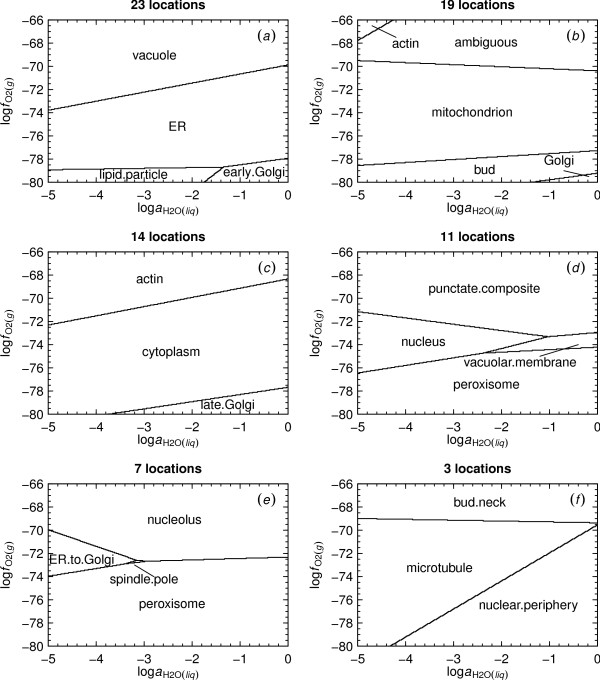
**Relative metastabilities of reference model proteins for compartments**. Predominance diagrams were generated as a function of log  and log  at 25°C and 1 bar for the reference model proteins listed in Table 3. The diagram in (*a*) represents 23 reference model proteins; diagrams in panels (*b*)-(*f*) represent systems with successively fewer reference model proteins as candidates.

The reference model proteins used in this study represent the overall amino acid compositions of the proteins in individual compartments. The amino acid compositions of reference model proteins for 23 subcellular compartments were calculated as described in the Methods and are listed in Additional File 2; the chemical formulas and standard molal Gibbs energies are listed in Table 3. The predominance diagrams in Fig. 2 depicting the relative metastabilities of the reference model proteins as a function of log  and log  were generated in sequential order. The first diagram in this figure corresponds to a system in which all 23 reference model proteins were considered. Subsequent diagrams in Fig. 2 were generated by eliminating from consideration some or all of the reference model proteins represented by predominance fields in the immediately preceding diagram. It can be seen in Fig. 2a that consideration of 23 reference model proteins resulted in predicted predominance fields for four proteins over the ranges of log  and log  shown in the diagram. The reference model proteins appearing in successive diagrams in Fig. 2 are characterized by increasingly higher predicted energy requirements for their formation. Hence, the mitochondrial, nuclear and cytoplasmic reference model proteins appearing in Fig. 2b–d are relatively less metastable compared to those of early Golgi and ER appearing in Fig. 2a.

**Table 3:** Chemical formulas, standard molal Gibbs energies and other properties for reference model proteins for compartments in yeast cells^a^.

Location	Number	Length	Formula		*Z*		log
actin	22	469.41	C_2316_._66_H_3636_._43_N_632_._31_O_721_._64_S_10_._01_	-18506	-5.3	-0.119	-74.7
ambiguous	123	572.02	C_2816_._55_H_4465_._61_N_759_._39_O_870_._87_S_17_._91_	-22616	-9.6	-0.146	NA
bud	57	462.56	C_2366_._32_H_3668_._44_N_614_._02_O_681_._16_S_20_._19_	-16641	5.4	-0.179	-75.4
bud.neck	11	905.26	C_4543_._68_H_7203_._75_N_1250_._35_O_1443_._95_S_26_._67_	-38103	-16.8	-0.113	-69.2
cell.periphery	38	826.07	C_4178_._75_H_6505_._8_N_1098_._18_O_1229_._93_S_33_._77_	-30641	1.7	-0.164	-79.4
cytoplasm	746	436.12	C_2164_._42_H_3440_._33_N_590_._61_O_659_._94_S_12_._66_	-17065	-2.7	-0.149	-73.5
early.Golgi	9	622.83	C_3198_._27_H_5068_._06_N_821_._28_O_972_._56_S_21_._53_	-25441	-19.7	-0.193	-78.0
endosome	30	484.06	C_2441_._34_H_3871_._24_N_661_._02_O_767_._51_S_14_._17_	-20265	-12.1	-0.133	-75.8
ER	197	245.57	C_1206_._09_H_1897_._94_N_314_._1_O_365_._15_S_8_._58_	-9276	-3.0	-0.173	-77.8
ER.to.Golgi	5	595.02	C_2951_._77_H_4601_._25_N_790_._99_O_907_._31_S_18_._89_	-22861	-13.3	-0.127	NA
Golgi	14	481.43	C_2502_._96_H_3880_._54_N_651_._84_O_740_S_13_._63_	-18516	-5.4	-0.167	-77.6
late.Golgi	29	787.94	C_4015_._6_H_6312_._79_N_1044_._15_O_1217_._15_S_22_._72_	-31255	-24.7	-0.174	-77.4
lipid.particle	17	501.83	C_2573_._36_H_3985_._56_N_672_._88_O_751_._67_S_17_._52_	-18687	-4.1	-0.167	-75.0
microtubule	10	497.13	C_2509_._19_H_3969_._36_N_690_O_774_._61_S_17_._95_	-20031	-4.0	-0.125	-75.0
mitochondrion	426	402.95	C_1987_._87_H_3166_._52_N_542_._82_O_596_._6_S_13_._18_	-15221	3.3	-0.160	-75.9
nuclear.periphery	46	815.58	C_4111_H_6516_._46_N_1092_._04_O_1272_._05_S_20_._55_	-33146	-11.5	-0.159	-77.0
nucleolus	60	605.43	C_2990_._75_H_4768_._81_N_820_._31_O_957_._91_S_14_._52_	-25444	-10.4	-0.121	-75.6
nucleus	453	339.30	C_1683_._49_H_2686_._85_N_472_._89_O_517_._13_S_8_._7_	-13342	3.4	-0.129	-75.0
peroxisome	18	422.30	C_2117_._28_H_3334_._08_N_568_._6_O_641_._97_S_13_._56_	-16400	-2.0	-0.150	-74.8
punctate.composite	61	467.64	C_2320_._38_H_3662_._92_N_633_._84_O_751_._45_S_10_._52_	-19985	-22.3	-0.102	NA
spindle.pole	30	398.53	C_1996_._48_H_3176_._93_N_555_._45_O_642_._49_S_12_._63_	-17251	-13.5	-0.100	-79.5
vacuolar.membrane	45	709.78	C_3532_._93_H_5555_._77_N_943_._21_O_1075_._43_S_23_._29_	-27439	-15.3	-0.150	-73.4
vacuole	67	428.85	C_2078_._86_H_3186_._53_N_542_._71_O_668_._01_S_14_._34_	-17065	-18.1	-0.093	-73.2

**a**. Chemical formulas of nonionized reference model proteins and standard molal Gibbs energy of formation from the elements ( in kcal mol^-1^, at 25°C and 1 bar) and net ionization state (*Z*) at pH = 7 of ionized reference model proteins were calculated using the amino acid compositions given in Additional File 2. Values of the nominal oxidation state of carbon () were calculated using Eqn. (6). The model log  values for the compartments were obtained from the metastability limits of subcellular interactions listed in Table 4 (see text).

Can the relative metastabilities of proteins be linked to the order of their appearance in the cell cycle? It is noteworthy that the reference model proteins representing the two cytoskeletal systems in yeast cells, actin and microtubule, appear near opposite ends of the energy spectrum. This outcome may be consistent with the observation that actin in different forms appears to be present at most stages of the cell cycle [43], but that the microtubule cytoskeleton grows during anaphase (i.e., the stage of the cell cycle characterized by physical separation of the chromosomes [44]) and is degraded during other stages of the cell cycle [43,44]. The outcome of the mitotic cycle in *S. cerevisiae *is the growth of a new cell in the form of a bud [44]. Not all structures in the bud form simultaneously. Instead, it has been observed that [45]"the endoplasmic reticulum, Golgi, mitochondria, and vacuoles all begin to populate the bud well before anaphase and that their segregation into the bud does not require microtubules". From Fig. 2 it is apparent that the proteins in the vacuole, ER, mitochondria and Golgi are all energetically less costly than many of their counterparts in other subcellular locations. The proteins in actin and lipid particles are also relatively metastable, which could imply that they too have a primary position in the formation of new cells. These and other potential consequences of energetic differences between the biomacromolecules in subcellular compartments have not been fully explored.

### Intercompartmental protein interactions

The diagrams in Fig. 2 show the metastability limits for interactions between predominant reference model proteins for different subcellular compartments. However, many subcellular interactions may in fact be meta-metastable with respect to the reaction boundaries shown in Fig. 2. For example, interactions occur between proteins in the cytoplasm and nucleus [46], but the reference model proteins for these compartments do not share a reaction boundary in Fig. 2. Below, known intercompartmental interactions are combined with the oxygen fugacity requirements for equal activities of the reference model proteins to characterize compartmental oxidation-reduction potentials.

To assess the biochemical evidence for specific interactions between proteins in different compartments in yeast cells, a series of review papers was surveyed [43,47,46,50]. The identified source statements are listed in Additional File 3, and simplified pairwise representations of the interactions are summarized in Table 4. Of 190 possible combinations between any two of the 20 subcellular compartments (this count excludes the ambiguous location and ER to Golgi and punctate composite, which did not appear in the literature survey), 46 interactions were identified through this survey.

**Table 4:** Major intercompartmental protein interactions in yeast^a^.

Interaction		log	Interaction		log
**actin**-**bud**	0.262	-74.9	**vacuole**-**bud**	0.384	-75.3
**actin**-bud.neck	0.078	-83.5	**vacuole**-**cell periphery**	0.305	-75.7
**actin**-**cell periphery**	0.183	-75.3	**vacuole**-**cytoplasm**	0.188	-73.4
**actin**-**endosome**	0.129	-75.6	**vacuole**-endosome	0.251	-75.9
actin-**vacuolar membrane**	0.081	-73.3	**vacuole**-**late Golgi**	0.358	-75.4
actin-**mitochondrion**	0.049	-64.2	**nucleus**-actin	-0.039	-74.3
**actin**-microtubule	0.123	-78.3	**nucleus**-microtubule	0.084	-80.1
microtubule-**bud**	0.139	-71.8	**nucleus**-spindle pole	0.014	-82.3
**microtubule**-**bud neck**	-0.045	-69.3	nucleus-**bud**	0.223	-74.9
microtubule-**cell periphery**	0.060	-69.3	**nucleus**-bud neck	0.039	-92.5
microtubule-**cytoplasm**	-0.057	-89.8	nucleus-**cytoplasm**	0.027	-59.7
microtubule-**spindle pole**	-0.070	-79.7	**nucleus**-nucleolus	-0.031	-70.0
spindle.pole-**cytoplasm**	0.013	-35.1	**nuclear periphery**-**bud neck**	-0.081	-69.4
spindle.pole-**nuclear periphery**	0.106	-76.3	nuclear periphery-**cytoplasm**	-0.093	-82.0
**ER**-cell.periphery	0.142	-82.3	**nuclear periphery**-**nucleus**	-0.120	-77.0
**ER**-cytoplasm	0.025	-96.5	**nuclear periphery**-nucleolus	-0.152	-75.5
**ER**-**early Golgi**	0.259	-78.0	**peroxisome**-**cell periphery**	0.064	-78.7
**ER**-nuclear.periphery	0.118	-85.0	peroxisome-**cytoplasm**	-0.053	-80.0
**ER**-peroxisome	0.078	-85.3	**peroxisome**-**lipid particle**	0.140	-74.9
**Golgi**-**endosome**	-0.205	-75.9	peroxisome-**mitochondrion**	-0.071	-80.0
**Golgi**-**vacuole**	-0.456	-75.9	**mitochondrion**-**cell periphery**	0.135	-79.4
**Golgi**-**late Golgi**	-0.097	-77.5	**mitochondrion**-cytoplasm	0.017	-79.9
Golgi-**early Golgi**	-0.034	-89.0	**mitochondrion**-nucleus	-0.010	-23.6

**a**. Interactions between proteins in different subcellular locations in *S. cerevisiae *were identified in the literature. The calculated reaction coefficients on O_2(*g*) _and the metastable equilibrium value of log  were calculated for each reaction between reference model proteins. Names of locations shown in bold indicate that the model value of log  for this compartment (Table 3) lies in the metastability range for the reference model protein in the particular reaction.

Chemical reactions corresponding to each of the interactions listed in Table 4 are listed in Additional File 4. The values of  (coefficient on O_2(*g*) _in the reactions) are listed in Table 4 together with the values of log  calculated for equal chemical activities of the two reference model proteins in each reaction. Note that there are some reactions where the absolute value of  is substantially smaller than the others; these include spindle pole-cytoplasm and mitochondrion-nucleus. Because of the small value of  in these reactions, the values of log  for equal activities of these proteins tend to be more extreme than for other reactions. The sign of  denotes the thermodynamically favored direction of the reaction as log  is changed from its equal-activity value; for example, at log  = -74.9, the reference model proteins of actin and bud can metastably coexist with equal chemical activities, but at higher values that of actin predominates in a metastable assemblage.

The interactions listed in Table 4 were used to obtain model values of the oxygen fugacity in each compartment that are listed in Table 3. The model value of the oxygen fugacity for each compartment was selected so that in as many cases as possible the reactions listed in Table 4 favor the formation of the reference model protein for this compartment relative to those of interacting compartments. For example, the log  listed for the actin compartment is -74.7, which allows this reference model protein to be metastable relative to its interacting partners in the first four reactions listed in Table 4. The limits of the model values of log  were set to between ca. -70 and -80, so that some of the more extreme values listed in Table 4 were not considered in the analysis.

It is notable that at log  = -75, the reference model protein for microtubule is not metastable with respect to any of its interacting partners except for bud neck. The reference model protein for microtubule only becomes relatively metastable at high oxygen fugacities (w.r.t. bud and cell periphery) or at low oxygen fugacities (w.r.t. cytoplasm and spindle pole). Hence, the value of log  -75 taken here for the microtubule compartment is different from the values for other compartments, in that this represents conditions where the formation of its reference model protein is more unfavorable than that of most of its interacting partners.

### Calculation of relative abundances of proteins

Above, the interactions between subcellular homologs of enzymes and reference model proteins for subcellular compartments were used to derive oxygen fugacity limits for metastable reactions of proteins in different compartments. In the second part of this study, attention is focused on the relative abundances and intracompartmental interactions of proteins.

**Figure 3:**
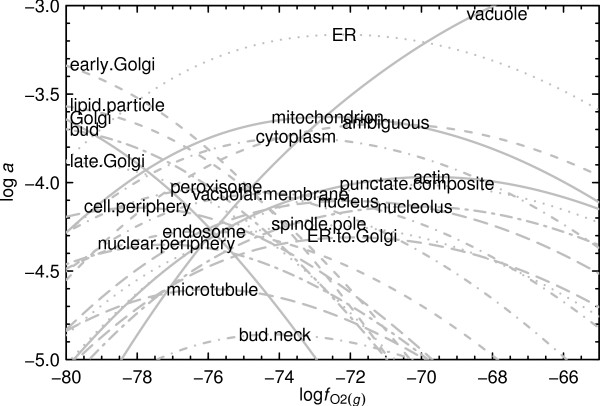
**Metastable equilibrium abundances of reference model proteins and proteins as a function of oxygen fugacity**. The relative abundances of the reference model proteins identified in Table 1 were calculated as a function of log  at 25°C and 1 bar and with total activity of amino acid residues equal to unity.

The logarithms of activities consistent with metastable equilibrium among all 23 reference model proteins are plotted in Fig. 3 as a function of log . The relative abundances of the proteins were calculated as described in Ref. [19] for reactions that conserve amino acid residues; a specific example of this type of calculation is described in the Methods. Fig. 3 presents in a different form the relationships shown in Fig. 2a at log  = 0. Note that the same proteins predominate at the extremes of oxygen fugacity represented in Fig. 3 and in Fig. 2a (reducing – early Golgi; oxidizing – vacuole) and that the reference model protein of microtubule appears with low relative abundance. Also note that there is a minimum in the range of calculated activities of the reference model proteins around log  = -75 to -76; changing oxidation-reduction potential alters not only the identity of the predominant protein in a metastably interacting population but also the relative abundances of all the others. There is probably not a single value of log  where the calculated relative abundances of the reference model proteins shown in Fig. 3 reflect the composition of the cell. Let us therefore look more closely at the relative abundances of proteins within compartments.

### Relative abundances of proteins within compartments

To model each of the compartments, up to 50 experimentally most abundant proteins were identified using data from Ref. [9]. The proteins that were selected were localized exclusively to each compartment, except for those of the bud. The numbers of proteins used to model each compartment are listed in Table 5, and the names of the proteins together with computational results given in Additional File 5.

**Table 5:** Oxygen fugacities, root mean square deviations and correlation coefficients in comparisons of intracompartmental protein interactions^a^.

Most abundant proteins					Model complexes				
	
Location	*n*	log	RMSD	*ρ*	Complex	*n*	log	RMSD	*ρ*
actin	22	-75.5	0.61	0.19	1	5	-77.0	0.49	-0.10
ambiguous	50	-73.5	0.90	0.42	2	7	-76.5	0.52	0.50
bud	50	-72.5	1.17	-0.02	3	5	-73.5	0.53	-0.30
bud neck	11	-75.5	0.73	0.02	4	6	-78.5	0.56	0.66
cell periphery	38	-74.5	0.63	0.42	5	4	-74.5	0.45	0.20
cytoplasm	50	-78.0	1.09	0.19	6	7	-78.5	0.64	-0.82
early Golgi	9	-74.0	0.72	0.45	7	4	-76.0	0.66	-0.80
endosome	30	-75.5	0.86	0.28	8	4	-76.5	0.76	-0.80
ER	49	-76.0	0.97	0.03	9	3	-77.0	0.07	1.00
ER to Golgi	5	-78.0	0.40	0.40	10	4	-76.0	0.45	1.00
Golgi	14	-76.0	0.88	-0.54	11	10	-74.5	0.62	-0.04
late Golgi	29	-76.0	0.73	0.17	12	5	-75.0	1.15	0.60
lipid particle	17	-78.0	0.92	0.22	13	12	-76.0	0.93	-0.19
microtubule	10	-75.0	0.61	0.36	14	7	-74.5	1.04	-0.75
mitochondrion	50	-75.0	0.53	0.46	15	17	-77.5	0.44	0.30
nuclear periphery	46	-76.0	0.62	0.32	16	23	-76.0	0.43	0.52
nucleolus	50	-74.0	0.72	0.18	17	6	-77.5	0.26	1.00
nucleus	50	-75.0	0.80	-0.02	18	5	-78.5	0.24	0.90
peroxisome	18	-75.5	0.55	0.56	19	8	-75.5	0.65	0.57
punctate composite	49	-74.0	0.78	0.19	20	15	-74.0	0.68	0.64
spindle pole	30	-75.5	1.02	0.12	21	5	-72.0	0.78	0.80
vacuolar membrane	45	-74.0	0.92	0.48	22	15	-74.0	0.94	0.50
vacuole	50	-74.5	1.42	0.23	23	9	-75.0	0.71	0.52

**a**. Values of log  in each location were obtained by comparing calculated and experimental logarithms of activities of the most abundant proteins in different subcellular compartments and of model complexes for each location (Additional File 6). *n *denotes the number of model proteins used in the calculations. RMSD values were calculated using Eqn. (7), and ρ denotes the Spearman rank correlation coefficient, calculated using Eqn. (8).

**Figure 4:**
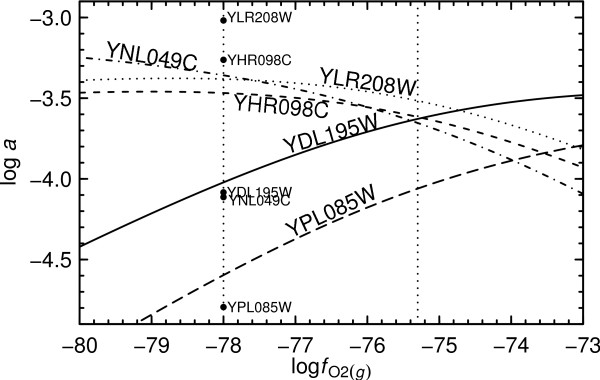
**Metastable equilibrium abundances of reference model proteins and proteins as a function of oxygen fugacity**. The relative abundances of five proteins localized to ER to Golgi whose experimental abundances were reported in [9] were calculated as a function of log  at 25°C and 1 bar and with total activity of amino acid residues equal to unity. The rightmost dotted line indicates conditions where the calculated abundance ranking of the proteins is identical to that found in the experiments, and the leftmost dotted line where the calculated logarithms of activities have a lower overall deviation from experimental ones, which are indicated by the points. This value of log  (-78) was used to construct the corresponding diagram in Fig. 5.

In Fig. 4 the relative abundances of the five model proteins localized exclusively to ER to Golgi are shown as a function of log . A worked-out example of the calculations leading to this figure is described in the Methods. The results of the calculations described there correspond to the dotted line at log  = -75.3 in Fig. 4. At this oxygen fugacity, the rank order of abundances of the model proteins in metastable equilibrium is identical to the rank order of experimental abundances. The figure was generated in whole by carrying out the calculation for different reference values of log . There is a narrow range on either side of log  = -75.3 (ca. ± 0.05) where the relative abundances of the proteins in metastable equilibrium occur in the same rank order. Beyond these limits, changing  drives the composition of the metastable equilibrium assemblage to other states that do not overlap as closely with the experimental rankings.

The experimental abundances of the proteins reported by [9] are 21400, 12200, 1840, 1720 and 358, respectively, in relative units. These abundances were scaled to the same total activity of amino acid residues (unity) used in the calculations to generate the experimental relative abundances plotted at the dashed line in Fig. 4 at log  = -78. Under these conditions, the metastable equilibrium abundances of the proteins do not occur in exactly the same rank order as the experimental ones, but there is a greater overall correspondence with the experimental relative abundances.

**Figure 5:**
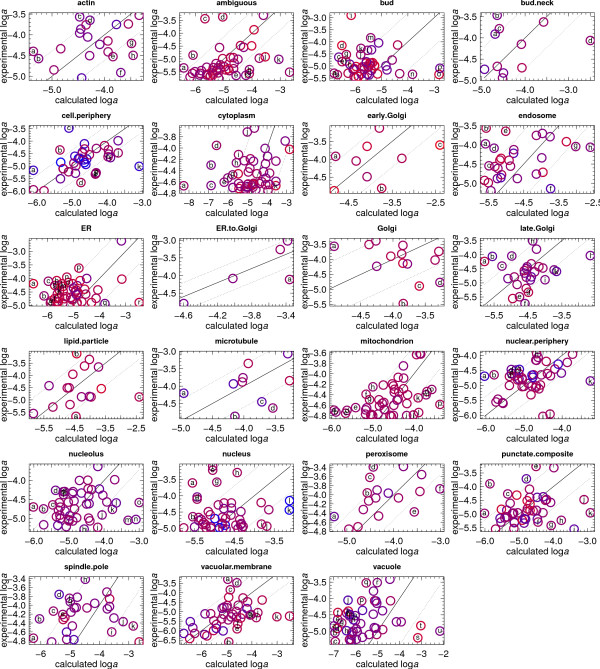
**Comparison of experimental and calculated logarithms of activities of proteins in compartments**. Red and blue colors denote, respectively, low and high average nominal carbon oxidation states () of the protein. Dotted lines are positioned at one RMSD above and below one-to-one correspondence, which is denoted by the solid lines. Outlying points are labeled with letters that are keyed to the proteins in Additional File 5. The values of log  used in the calculations are listed in Table 5.

Similar calculations were repeated for each of the other compartments identified in Ref. [8]. The relative abundances of the proteins were calculated at 0.5 log unit increments from log  = -82 to -70.5. Scatterplots of the experimental vs. calculated relative abundances are shown in Additional File 6. These comparisons were assessed to obtain values of log , listed in Table 5, that yield the best fit between calculated and experimental relative abundances. The best-fit calculated relative abundances are listed together with the experimental ones in Additional File 5, and the corresponding best-fit scatterplots for each set of model proteins are shown in Fig. 5.

The retrieval of optimal values of log  was aided by calculating the root mean square deviation (RMSD) of logarithms of activities using Eqn. (7) and the Spearman rank correlation coefficient (*ρ*; Eqn. 8) between experimental and calculated logarithms of activities. The dotted lines in Fig. 5 were drawn at one RMSD on either side of the one-to-one correspondence, denoted by the solid lines in this figure. The RMSD values were used to identify outliers that are identified in Fig. 5 by letters that are listed in Additional File 5. To aid in distinguishing the points, they were assigned colors on a red (reduced) – blue (oxidized) scale that reflects the average nominal oxidation state of carbon of the protein (Eqn. 6).

There is a considerable degree of scatter apparent in many of the plots shown in Fig. 5, so a low degree of certainty may be associated with the log  values regressed from these comparisons. In specific cases such as peroxisome and nuclear periphery a lower overall deviation is apparent and a positive correlation appears between the calculated and experimental relative abundances. Because they were regressed from intracompartmental protein abundance data, the values of log  listed in Table 5 might not be as representative of subcellular oxidation-reduction conditions as those listed in Table 3, which have the additional benefit of being based partly on known subcellular interactions (see above).

The comparisons depicted in Fig. 5 and in Additional File 6 are also significant because they reveal that the range of protein abundances observed in cells is accessible in a metastable equilibrium assemblage at some values of log . For example, the range of experimental abundances of the model proteins in actin covers about 1.6 orders of magnitude, while the calculated abundances vary over about 2.2 orders of magnitude. Extreme values of log  tend to weaken this correspondence. The lowest degree of correspondence occurs for the cytoplasmic proteins, where ~5 orders of magnitude separate the predicted relative abundances of the top 50 most abundant proteins, which in the experimental measurements have a dynamic range spanning about 1.2 orders of magnitude. The great degree of scatter apparent in many of the comparisons in Fig. 5 could be partly a consequence of including in the comparisons model proteins that do not actually interact with each other, despite their high relative abundances. To address this concern, a more directed approach was adopted below that takes account of fewer numbers of proteins that are known to interact through the formation of complexes.

### Relative abundances of proteins in complexes

The correspondence between the calculated and experimental relative abundances of the five model proteins in ER to Golgi raises the question of what characteristics of the proteins might be responsible for this result. Scanning the functional annotations of these proteins reveals that they are part of the COPII coat complex [51]. The results for this model system suggested that focusing on specific complexes in other compartments could yield interesting results. Because the interactions of proteins to form complexes is essential in cellular structure and regulating the functions of enzymes [12], factors that affect the relative abundances of the complexing proteins may be fundamental to the control of metabolic processes.

The model complexes used in this study are identified in Table 6 and the individual proteins in each complex are listed in Additional File 7. Each complex was nominally associated with a subcellular compartment based on the names and descriptions of the complexes available in the literature. Some exceptions are the cyclin-dependent protein kinase complex, the proteins of which are largely cytoplasmic and nuclear [8], but here is placed in the slot for the ambiguous location because no definitely ambiguously localized complexes could be identified. The proteins listed in Additional File 7 under punctate composite are not part of a named complex but were chosen because they are localized to early Golgi and have the punctate composite characterization [8]. The other exceptions are the vacuolar model proteins (proteases and other canonical vacuolar proteins [52]), enzymes of the ergosterol biosynthetic pathway, some of which are associated with the lipid particle [53], and proteins integral to the peroxisomal membrane, which were identified using the Gene Ontology (GO) annotations in the SGD [51].

**Table 6:** List of selected complexes^a^.

#	Location	Complex	References
1	actin	Arp2/3 complex	[68,69] (423)
2	ambiguous	cyclin-dependent protein kinase complex	(343)
3	bud	actin-associated motor protein complex 2	[70] (49)
4	bud.neck	septin complex	[71] (333)
5	cell.periphery	exocyst complex	(120)
6	cytoplasm	translation initiation factor eIF3	(45)
7	early.Golgi	SNARE complex	[72] (113)
8	endosome	ESCRT I & II complexes	[73,74]
9	ER	signal recognition complex	(52)
10	ER.to.Golgi	coatomer COPII complex	(340)
11	Golgi	Golgi transport complex	(293)
12	late.Golgi	retrograde protein complex	[75] (114)
13	lipid.particle	sterol biosynthesis enzymes	[53]
14	microtubule	DASH complex	[76]
15	mitochondrion	mitochondrial ribosome small subunit	(9)
16	nuclear.periphery	nuclear pore complex	[77]
17	nucleolus	small subunit processome	[78] (70)
18	nucleus	RNA polymerase I	(30)
19	peroxisome	integral to peroxisomal membrane	(GO:0005779)
20	punctate.composite	proteins localized here and early.Golgi	
21	spindle.pole	spindle-pole body complex	[79] (219)
22	vacuolar.membrane	VO vacuolar ATPase complex	(14)
23	vacuole	vacuolar proteases and other canonical proteins	[52]

**a**. Numbers in parentheses refer to the ID number of the complex, if available, from . embl.de [80]. Compositions and localizations of complexes were also taken from references listed in square brackets.

The calculated metastable equilibrium logarithms of activities of the proteins in each complex are shown as a function of log  in Additional File 8. The calculated logarithms of activities of the proteins were compared with experimental ones by constructing scatterplots at 0.5 log unit intervals from log  = -82 to -70.5, which are shown in Additional File 6. As described above, visual assessment of fit was used in combination with the RMSD and Spearman rank correlation coefficients to obtain values of log  that maximize the correspondence with experimental relative abundances. The resulting calculated relative abundances are listed together with the experimental ones in Additional File 9.

**Figure 6:**
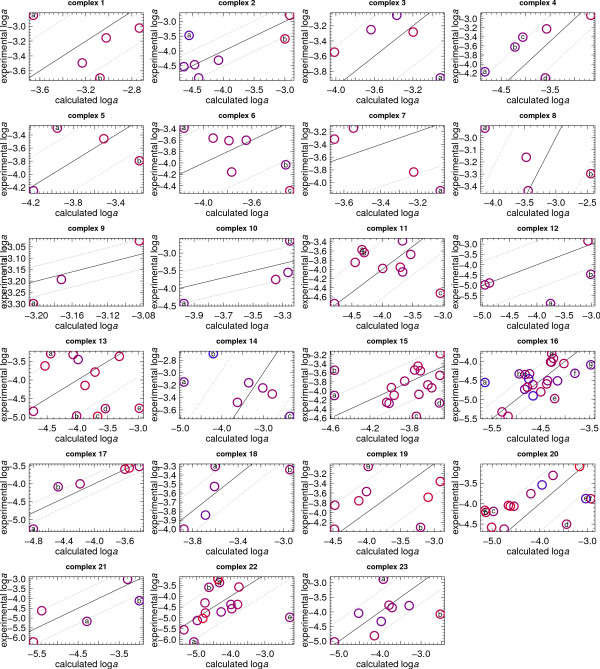
**Comparison of experimental and calculated logarithms of activities of model proteins for complexes**. Symbols are as in Fig. 5; the model proteins and the outliers (identified by letters) are listed in Additional File 9.

The number of model proteins in the complexes is less than the number of most abundant proteins in the compartments considered in the preceding section. Some of the model complexes represented in Fig. 6 exhibit an apparent positive correlation between calculated and experimental logarithms of activities; these include nuclear pore complex and small subunit processome. A negative correlation between calculated and experimental logarithms of activities is apparent for proteins in the ESCRT I & II complexes and DASH complex. A few of the other complexes (Golgi transport complex, sterol biosynthesis enzymes) exhibit very little overall correspondence between calculated and experimental logarithms of activities.

The results in Fig. 6 permit an interpretation of the relative energetic requirements for formation of different groups of interacting proteins. Take for example complex 14, which is the DASH complex that associates with the microtubule. A negative correlation between the experimental and calculated relative abundances is apparent for this complex in Fig. 6. The RMSD between calculated and experimental logarithms of activities of proteins is 1.04, which is among the highest listed in Table 5. Note from Eqn. (11) that a ~1 log unit change in the chemical activity of a chemical species corresponds to a Gibbs energy difference equal to 2.303*RT*. An average difference of ~1 between calculated and experimental logarithms of activity indicates that the formation of the proteins requires 2.303*RT *= 1364 cal mol^-1 ^beyond what would be needed if the proteins formed in metastable equilibrium relative abundances. On the other hand, the formation in specific oxidation-reduction conditions of proteins making up other assemblages where cellular abundances positively correlate with and span the same range as the metastable equilibrium distribution can proceed close to a local minimum energy required for protein formation.

Because of their relatively high energy demands, proteins in complexes such as the DASH complex and the spindle pole body are likely to be more dynamic in the cell. (Note that although a positive rank correlation coefficient for the latter complex is reported in Table 5, at a lower oxygen fugacity (log  = -77.5) an inverse correlation results between experimental abundances and calculated metastable equilibrium relative abundances of the proteins in this complex; see Additional File 6). The finding made elsewhere of some inverse relationships between relative abundance of proteins and corresponding mRNA levels was interpreted as evidence for additional effort on the part of the cell [54]. An inverse relationship that opposes equilibrium may be favored in evolution because of the strategic advantage of incorporating otherwise costly (rare) amino acids that increase enzymatic diversity [55].

The differences in the numbers of proteins considered in each of the comparisons implies that the values of the correlation coefficients are not directly comparable. The p-values for each of the correlations listed in Table 5 were calculated and are reported in Additional File 10. The p-value is the probability that the value of the observed correlation coefficient can be met or exceeded by a random configuration of the system. The present calculations suggest that the lowest p-values are associated with the collections of greater than ca. 40 proteins listed in Table 5 that have a Spearman rank correlation coefficient greater than ca. 0.4. Using the p-value as a criterion, the most convincing demonstrations of the existence of correlations appear in the most abundant proteins of the vacuolar membrane and cell periphery. By comparison, the smaller systems of proteins making up complexes, which in some cases have higher correlation coefficients, also have relatively high p-values, indicating a greater probability that the same result can be obtained in a random configuration.

### Implications of the hypothesis

Tests of several specific predictions of the hypothesis were discussed in the preceding sections. The major results of these calculations and comparisons are listed below.

1. Subcellular homologs of glutaredoxin and thioredoxin are metastable at different log  ranges. The mitochondrial homolog appears to be more reduced, and the nuclear one most oxidized. Reactions within the glutaredoxin system also exhibit sensitivity to hydration state.

2. Reference model proteins for 23 subcellular locations also have metastability limits in log  space. The relationships are consistent with a relatively oxidized nuclear reference model protein, but that for the mitochondrion is intermediate between the nucleus and the cytoplasm. Among the reference model proteins predicted to be most stable (Fig. 2a–b), four are known to be involved in the early stages of formation of the bud. Golgi is predicted to be a reduced compartment while actin and the vacuole are relatively oxidized.

3. The least stable reference model proteins are those for the microtubule and bud neck (Fig. 2f and Fig. 3). This observation suggests that the proteins in the microtubule are very reactive with other cellular components, and/or that they have a relatively high turnover rate.

4. Observed trends in the relative abundances of the most abundant proteins in some compartments can be correlated with the relative abundances of proteins predicted using a metastable equilibrium model. Correlations between observed and predicted relative abundances for smaller numbers of proteins that make up complexes can also be documented. In some cases negative correlations may be supported, such as for the DASH complex (microtubule), translation initiation factor, and the early Golgi SNARE complex. The maintenance of these complexes might entail a higher energy demand than for others in the cell.

If the hypothesis adopted for this study was true, it would imply that there are processes that impart an energetic bias on the appearance of proteins in specific compartments. The thermodynamic model described above by itself gives no information about the possible nature of processes involved. Two processes that could be important are the work against diffusional gradients required for active protein transport and the turnover rates of subcellular populations of proteins. Regarding the former, it is the gradient of chemical potential (not concentration) of the biomacromolecule, that appears in statements such as Fick's Law. Differences in the chemical conditions between compartments would be expected to differentially contribute to the activity coefficients of proteins, so that the cost of transport to various compartment is not equal. Although the activity coefficients of proteins were not considered in this study, their values might depend on oxidation or hydration potential, so the the current results could be implicitly influenced by nonideality in the subcellular system. Regarding the latter process, one may expect that the turnover rates of proteins are tied to the local chemical environment. If, for example, the turnover rate of a population of proteins minimizes at a specific oxygen fugacity, then any deviations away from this oxidation potential would increase the turnover rate and cause the cell to expend more energy in maintaining this population.

If chemical energy minimization by the cell results in an increase in fitness, the energetic effects of the physical-chemical processes outlined above may constrain the overall process of natural selection. Therefore, the hypothesis also implies that an expected outcome of evolution is the formation of biomacromolecules with lower energy demands compared with other possible, and otherwise equal, products. How does this connect with the mechanism of protein transport and trafficking, i.e. that from their place of synthesis (ribosomes) proteins are transported to different subcellular locations, often under the influence of specific signal sequences? At one extreme, it is possible that the energetic differences between compartments are not influential in the evolution of the mechanism of protein sorting and trafficking. However, if the signal sequences themselves are chemically reactive to varying degrees depending on their subcellular environment, then selection for mutations in them might be tuned to both function and chemistry. It would not be surprising then to find evidence for the chemical adaptation of signal sequences to specific compartments.

These results and observations support the notion that changing oxidation-reduction potential can selectively alter the potential for reactions leading to formation of proteins and their complexes. Chemical selectivity in the dynamic formation in the cell of high-energy proteins could lead to transient formation of complexes that function only under certain conditions. Because of the different stability limits of the reference model proteins in log  space, these results also in principle support the notion that "a fundamental redox attractor underpins ... core cellular processes"[56]. In reality, many chemical properties vary spatially in cells, including the hydration state, pH, activities of CO_2 _and H_2_S, and temperature and pressure in the extracellular environment. These all factor into the Gibbs energy changes accompanying the chemical transformations between proteins, as do the thermodynamic properties of protein folding reactions and nonideality in protein solutions. Because of its energetic basis, the model used here can be extended in the future to incorporate the effects of these variables. Building these relationships into a multidimensional thermodynamic assessment is a promising avenue for predicting the chemical features of proteomic adaptation in the context of the cellular environment.

## Methods

The essential steps in the calculations reported here are 1) defining standard states, 2) identifying model proteins for systems of interest, 3) assessing the relative abundances of model proteins in metastable equilibrium, 4) visualizing the results of the calculations on chemical diagrams and 5) comparing the computational results with experimental biochemical and proteomic data.

### Standard states and chemical activities

The activity of a species is related to the chemical potential of the species by

(1)

where *R *and *T *represent, respectively, the gas constant and the temperature, *μ *and *μ*○ stand for the chemical potential and standard chemical potential, respectively, and *a *denotes activity. No provision for activity coefficients of proteins or other species was used in this study; under this approximation, the activity of an aqueous species is equal to its concentration (molality).

The standard state for aqueous species including proteins specifies unit activity of the aqueous species in hypothetical one molal solution referenced to infinite dilution. The standard molal Gibbs energies of the proteins were calculated with the CHNOSZ software package [19] using group additivity properties and parameters taken from Ref. [23].

### Reference model proteins for amino acid compositions

The overall amino acid compositions of proteins in 23 subcellular locations in *S. cerevisiae *were calculated by combining localization [8] and abundance [9] data for proteins measured in the YeastGFP project with amino acid compositions of proteins downloaded from the *Saccharomyces *Genome Database (SGD) [51]. Of 4155 ORF names listed in the YeastGFP dataset, all but 12 are present in SGD (the missing ones are YAR044W, YBR100W, YDR474C, YFL006W, YFR024C, YGL046W, YGR272C, YJL012C-A, YJL017W, YJL018W, YJL021C and YPR090W).

To generate reference model proteins that are most representative of each compartment, proteins that were annotated in the YeastGFP study as being localized to more than one compartment were excluded from this analysis (except for bud; see below), as were those for which no abundance was reported. The names of the open reading frames (ORFs) corresponding to the proteins in the YeastGFP data set were matched against the SGD's protein_properties.tab file downloaded on 2008-08-04. This search yielded a number of model proteins for each compartment, ranging from 5 (ER to Golgi) to 746 (cytoplasm); see Table 3. The names of the compartments used throughout the tables and figures in this paper correspond to the notation used in the YeastGFP data files.

It was found that no proteins with reported abundances and localized to the bud were exclusive to that compartment, hence all of the proteins localized there (which also have localizations in other compartments) were taken as models for the bud reference model protein. The amino acid composition of the reference model protein for each compartment was calculated by taking the sum of the compositions of each model protein for a compartment in proportion to its fractional abundance in the total model protein population of the compartment. The resulting amino acid compositions are listed in Additional File 2. The corresponding chemical formulas of the nonionized reference model proteins and the calculated standard molal Gibbs energies of formation from the elements at 25°C and 1 bar of the ionized reference model proteins are shown in Table 3.

### Metastability calculations

Diagrams showing the predominant proteins and the relative abundances of proteins in metastable equilibrium were generated using the CHNOSZ software package [19]. These calculations take account of formation reactions of the proteins written for their residue equivalents [19]. An example of this approach is described further below for a specific model system.

The basis species appearing in the formation reactions studied here are CO_2(*aq*)_, H_2_O, NH_3(*aq*)_, O_2(*g*)_, H_2_S_(*aq*) _and H^+^. The reference activities used for the basis species were 10^-3^, 10^0^, 10^-4^, 10^-7 ^and 10^-7^, respectively, for CO_2(*aq*)_, H_2_O, NH_3(*aq*)_, H_2_S_(*aq*) _and H^+^. In the case of diagrams showing Eh as a variable, the aqueous electron (*e*^-^) was substituted for O_2(*g*) _in the basis species. Reference values for  or  are not listed here because one or the other is used as an independent variable in each of the calculations described above.

### Conversion between scales of oxidation-reduction potential

Conversion between the log  and Eh scales of oxidation-reduction potential can be made by first writing the half-cell reaction for the dissociation of H_2_O as

(2)

Taking pH = -log  and pe = -log , the logarithmic analog of the law of mass action for Reaction 2 can be written as:

(3)

where log *K*_2 _stands for the logarithm of the equilibrium constant of Reaction 2 as a function of temperature and pressure. Eh is related to pe by [57]

(4)

where *F *and *R *denote the Faraday constant and the gas constant, respectively. Combining Eqns. (3) and (4) yields the following expression for Eh as a function of log  and other variables:

(5)

At 25°C and 1 bar, *F*/2.303*RT *= 16.903 volt^-1 ^and log *K*_2 _= -41.55; for pH = 7 and log  = 0, a value of Eh = 0 V corresponds to log  = -55. Eqn. (5) permits the conversion between Eh and log  as well at other temperatures, pHs, and activities of H_2_O.

### Average nominal oxidation state of carbon

Let us write the chemical formula of a species of interest as  where *Z *denotes the net charge. The average nominal oxidation state of carbon () of this species is given by

(6)

Eqn. (6) is consistent with the electronegativity rules described in [58] and is compatible with the equation for average oxidation number of carbon used in [59]. For example, Eqn. (6) can be used to calculate the average nominal oxidation states of carbon in CO_2 _and CH_4_, which are +4 and -4, respectively. Note that the proportions of oxygen and other covalently bonded heteroatoms contribute to the value of  of a protein or other molecule, but that proton ionization does not alter the nominal carbon oxidation state, because of the opposite contributions from *Z *and *n*_H _in Eqn. (6). In the 4143 proteins identified in the YeastGFP subcellular localization study and found in the *Saccharomyces *Genome Database, the minimum and maximum of  are -0.414 and 0.390, respectively. Of the proteins in this dataset, six have  < -0.35 (YDR193W, YDR276C, YEL017C-A, YJL097W, YML007C-A, YMR292W) and six have  > 0.15 (YCL028W, YHR053C, YHR055C, YKR092C, YMR173W, YPL223C). The points in the scatterplots in this paper (Figs. 5 and 6 and Additional File 6) are colored on a continuous red-blue scale according to the value of  of the proteins, where maximum red occurs at  = -0.35 and maximum blue occurs at  = 0.15.

### Comparison with experimental relative abundances

The root mean square deviation between calculated and experimental logarithms of activities was calculated using

(7)

where *X*_calc, *i *_and *X*_expt, *i *_denote the calculated and experimental logarithms of activities and *n *stands for the number of proteins. In the calculations described above, experimental abundances of proteins in each model system were scaled so that the total chemical activity of amino acid residues was equal to unity.

The Spearman rank correlation coefficient (*ρ*) was calculated using

(8)

where  and *X*_calc, *i *_and *X*_expt, *i *_stand for the ranks of the corresponding logarithms of activities.

### Calculating relative abundances of proteins in metastable equilibrium

The following example demonstrates the procedure used to calculate the relative abundances of proteins in metastable equilibrium. The model proteins for ER to Golgi, in order of decreasing abundance in the cell reported by [9], are YLR208W, YHR098C, YDL195W, YNL049C and YPL085W. (For simplicity, the proteins are identified here by the names of the open reading frames (ORF).) The formula of the uncharged form of the first protein, YLR208W, is C_1485_H_2274_N_400_O_449_S_4_, and its amino acid sequence length is 297 residues. The standard molal Gibbs energy of formation from the elements () of this protein at 25°C and 1 bar calculated using group additivity [23] is -10670 kcal mol^-1^. At this temperature and pressure and at pH = 7, group additivity can also be used to calculate the charge of the protein (-10.8832) and the standard molal Gibbs energy of formation from the elements of the charged protein (-10880 kcal mol^-1^). The formula of the protein in this ionization state is C_1485_H_2263.1168_N_400_O_449 _. Dividing by the length of the protein, we find that the formula and standard molal Gibbs energy of formation from the elements of the residue equivalent of YLR208W are C_5.0000_H_7.6199_N_1.3468_O_1.5118 _ and -36.633 kcal mol^-1^, respectively.

The formation from basis species of the residue equivalent of YLR208W is consistent with

(9)

Similar reasoning can be applied to write the formation reaction of the residue equivalent of YHR098C as

(10)

At 929 residues, YHR098C is over 3 times as long as YLR208W, but in the formation reactions from the basis species of the residue equivalents of the two proteins, the coefficients on the basis species are similar. The difference between the coefficients of the same basis species in the reactions signifies the response of the metastable equilibrium assemblage to changes in the corresponding chemical activity or fugacity. For example, because  and  increasing ,  or  at constant *T*, *P *and chemical activities of the other basis species shifts the metastable equilibrium in favor of YLR208W at the expense of YHR098C. Here, *ν*_*i *_denotes the reaction coefficient of the *i*th basis species or protein, which is negative for reactants and positive for products as written. Conversely, because  and  increasing ,  or  (decreasing pH) at constant *T*, *P *and chemical activities of the other basis species shifts the metastable equilibrium in favor of YHR098C at the expense of YLR208W. The magnitude of the effect is proportional to the size of the difference between the coefficients of the basis species in the reactions, and it can be quantified for a specific model system using the following calculations.

To assess the relative abundances of the proteins in metastable equilibrium, we proceed by calculating the chemical affinities of each of the formation reactions. The chemical affinity (***A***) is calculated by combining the equilibrium constant (*K*) with the reaction activity product (*Q*) according to [60]

(11)

where 2.303 is the natural logarithm of 10, *R *stands for the gas constant, *T *is temperature in degrees Kelvin,  is the standard molal Gibbs energy of the reaction, and *a*_*i *_and *ν*_*i *_represent the chemical activity and reaction coefficient of the *i*th basis species or species of interest (i.e., residue equivalent of the protein) in the reaction. Let us calculate  (in kcal mol^-1^) of Reaction 9 by writing

(12)

In Eqn. (12) the values of  of O_2(*g*) _and H^+ ^are both zero, which are consistent with the standard state conventions for gases and the hydrogen ion convention used in solution chemistry. The values of  of the other basis species are taken from the literature [61-63]. The value of log *K*_9 _consistent with Eqn. (12) is -392.19.

We now calculate the activity product of the reaction using

(13)

The values of *a*_*i *_used to write Eqn. (13) are the reference values listed in the Methods for  and . The value of  used in Eqn. (13) (log  = -75.3) is also a reference value that, it will be shown, characterizes a metastable equilibrium distribution of proteins that is rank-identical to the measured relative abundances of the proteins. Finally, the value of *a *of the residue equivalent of the protein in Eqn. (13) is set to a reference value of unity (log *a *= 0). If we are only concerned with the relative abundances of the proteins in metastable equilibrium, the actual value used here does not matter so long as it is the same in the analogous calculations for the other proteins.

Combining Eqns. (11)–(13) yields ***A***_9_/2.303*RT *= -25.25 (this is a non-dimensional number). Following the same procedure for the other four proteins (YHR098C, YDL195W, YNL049C and YPL085W) results in ***A***/2.303*RT *equal to -24.86, -24.74, -24.93 and -24.94, respectively. Now let us turn to the relative abundances of the proteins in metastable equilibrium, which can be expressed in a manner analogous to a Maxwell-Boltzmann distribution:

(14)

where *a*_*t *_denotes the total activity of residue equivalents in the system and *n *stands for the number of proteins in the system. Note regarding the left-hand side of Eqn. (14) that because we are taking activity coefficients of unity, the ratio *a*_*i*_/*a*_*t *_is equal to the ratio of concentrations of residue equivalents in the system. No negative sign appears in front of ***A***/*RT *in the exponents Eqn. (14) because the chemical affinity is the negative of Gibbs energy change of the reaction. Note in addition that the values of ***A***/2.303*RT *given above must be multiplied by ln 10 = 2.303 before being substituted in Eqn. (14). By taking *a*_*t *_= 1, we can combine Eqn. (14) with ***A***/*RT *of each of the formation reactions to calculate chemical activities of the residue equivalents of the proteins equal to 0.0905, 0.2248, 0.2994, 0.1944 and 0.1909, respectively. The lengths of the proteins are 297, 929, 1273, 876 and 2195, so the corresponding logarithms of activities of the proteins are e.g. log (0.0905/297) = -3.52 for YLR208W, and -3.61, -3.63, -3.65 and -4.06 for the remaining proteins, respectively.

## Competing interests

The author declares that he has no competing interests.

## Authors' contributions

JMD conceived the study and wrote the manuscript.

## Supplementary Material

Additional file 1 **Program script and data files for generating figures**. This program script and supporting files were used to generate the figures shown above. To generate the figures, the contents of the zip file can be placed into the R working directory before loading the CHNOSZ package (version 0.8). Then read in the script with source('plot.R'). More details on the operation are provided at the top of the script file. Click here for file

Additional file 2 **Amino acid compositions of reference model proteins**. Overall amino acid compositions of proteins in subcellular locations of *S. cerevisiae *were calculated from YeastGFP localization [8] and abundance [9] data downloaded from  combined with protein compositions downloaded from the *Saccharomyces *Genome Database . The amino acid compositions of the reference model proteins were used to calculate the properties listed in Table 3. Click here for file

Additional file 3 **Interactions between subcellular compartments in yeast**. This file lists statements from Refs. [43,47,46,50] used to identify the interactions between proteins in different compartments of *Saccharomyces cerevisiae *that are listed in Table 4. Click here for file

Additional file 4 **Intercompartmental protein reactions**. This table lists chemical reactions between residue equivalents of reference model proteins for interactions identified above. The charges of the reference model proteins were calculated at 25°C, 1 bar and pH = 7. Click here for file

Additional file 5 **Abundance data for model proteins for compartments**. For the up to 50 most abundant model proteins in each compartment are listed the ORF name, sequence length, average nominal oxidation state of carbon (Eqn. 6), computed standard molal Gibbs energy at 25°C and 1 bar of the ionized protein and charge at pH = 7 and calculated and experimental logarithm of activity. This file also identifies the outlying points labeled with letters in Fig. 3. Click here for file

Additional file 6 **Abundance comparison for model proteins for compartments and complexes**. Scatterplots of experimental vs. calculated logarithm of activity of model proteins in subcellular compartments were generated for a range of logarithm of oxygen fugacity from -82 to -70.5. The legend of each diagram indicates the logarithm of oxygen fugacity ("O2"in the legend), root mean square deviation ("rmsd" in the legend; RMSD in Eqn. 7) and the Spearman rank correlation coefficient ("rr" in the legend; ρ in Eqn. 8). Click here for file

Additional file 7 **Identities of proteins in selected complexes**. Lists the proteins in the selected model complexes and whether their abundances are reported in the YeastGFP dataset. Proteins without experimental abundance data were not used in the comparisons discussed in this study. Click here for file

Additional file 8**Plots of relative abundances of model proteins for complexes**. The calculated relative abundances of model proteins in selected complexes are shown as a function of log .Click here for file

Additional file 9 **Abundance data for model proteins for complexes**. For model proteins in selected complexes (see Additional File 7) are listed the ORF name, sequence length, average nominal oxidation state of carbon (Eqn. 6), computed standard molal Gibbs energy at 25°C and 1 bar of the ionized protein and charge at pH = 7 and calculated and experimental logarithm of activity. Click here for file

Additional file 10 **Calculation of p-values for abundance rank correlations**. This file lists calculated p-values for the Spearman rank correlation coefficients and describes the steps used in the calculations. Click here for file

## References

[B1] Preston RA, Murphy RF, Jones EW (1989). Assay of vacuolar pH in yeast and identification of acidification-defective mutants. Proc Natl Acad Sci USA.

[B2] Hwang C, Sinskey AJ, Lodish HF (1992). Oxidized redox state of glutathione in the endoplasmic reticulum. Science.

[B3] Morrill GA, Kostellow AB, Osterlow K, Gupta RK (1996). Differences in hydration state of nucleus and cytoplasm of the amphibian oocyte. J Membrane Biol.

[B4] Al-Habori M (1995). Microcompartmentation, metabolic channelling and carbohydrate metabolism. Int J Biochem Cell Biol.

[B5] Aw TY, Walter H, Brooks DE, Srere PA (2000). Intracellular compartmentation of organelles and gradients of low molecular weight species. Microcompartmentation and Phase Separation in Cytoplasm, Int Rev Cytol.

[B6] Cedano J, Aloy P, Pérez-Pons JA, Querol E (1997). Relation between amino acid composition and cellular location of proteins. J Mol Biol.

[B7] Andrade MA, O'Donoghue SI, Rost B (1998). Adaptation of protein surfaces to subcellular location. J Mol Biol.

[B8] Huh WK, Falvo JV, Gerke LC, Carroll AS, Howson RW, Weissman JS, O'Shea EK (2003). Global analysis of protein localization in budding yeast. Nature.

[B9] Ghaemmaghami S, Huh W, Bower K, Howson RW, Belle A, Dephoure N, O'Shea EK, Weissman JS (2003). Global analysis of protein expression in yeast. Nature.

[B10] Gasch AP, Spellman PT, Kao CM, Carmel-Harel O, Eisen MB, Storz G, Botstein D, Brown PO (2000). Genomic expression programs in the response of yeast cells to environmental changes. Mol Biol Cell.

[B11] Schekman R (1985). Protein localization and membrane traffic in yeast. Annu Rev Cell Biol.

[B12] Doxsey S, McCollum D, Theurkauf W (2005). Centrosomes in cellular regulation. Annu Rev Cell Dev Biol.

[B13] Halvorson H (1958). Intracellular protein and nucleic acid turnover in resting yeast cells. Biochim Biophys Acta.

[B14] Morowitz HJ (1978). Foundations of Bioenergetics.

[B15] Seligmann H (2003). Cost-minimization of amino acid usage. J Mol Evol.

[B16] Swire J (2007). Selection on synthesis cost affects interprotein amino acid usage in all three domains of life. J Mol Evol.

[B17] Berezovsky IN, Zeldovich KB, Shakhnovich EI (2007). Positive and negative design in stability and thermal adaptation of natural proteins. PLoS Comput Biol.

[B18] Kondepudi DK, Prigogine I (1998). Modern Thermodynamics: From Heat Engines to Dissipative Structures.

[B19] Dick JM (2008). Calculation of the relative metastabilities of proteins using the CHNOSZ software package. Geochem Trans.

[B20] Wicken JS (1980). A thermodynamic theory of evolution. J Theor Biol.

[B21] Aita T, Husimi Y (1996). Fitness spectrum among random mutants on Mt. Fuji-type fitness landscape. J Theor Biol.

[B22] Demetrius L, Ziehe M (2007). Darwinian fitness. Theor Popul Biol.

[B23] Dick JM, LaRowe DE, Helgeson HC (2006). Temperature, pressure, and electrochemical constraints on protein speciation: Group additivity calculation of the standard molal thermodynamic properties of ionized unfolded proteins. Biogeosciences.

[B24] Pedrajas JR, Porras P, Martínez-Galisteo E, Padilla CA, Miranda-Vizuete A, Bárcena JA (2002). Two isoforms of *Saccharomyces cerevisiae *glutaredoxin 2 are expressed *in vivo *and localize to different subcellular compartments. Biochem J.

[B25] Molina MM, Bellí G, de la Torre MA, Rodríguez-Manzaneque MT, Herrero E (2004). Nuclear monothiol glutaredoxins of *Saccharomyces cerevisiae *can function as mitochondrial glutaredoxins. J Biol Chem.

[B26] Herrero E, Ros J, Tamarit J, Belli G (2006). Glutaredoxins in fungi. Photosynth Res.

[B27] Pedrajas JR, Kosmidou E, Miranda-Vizuete A, Gustafsson JA, Wright APH, Spyrou G (1999). Identification and functional characterization of a novel mitochondrial thioredoxin system in *Saccharomyces cerevisiae*. J Biol Chem.

[B28] Trotter EW, Grant CM (2005). Overlapping roles of the cytoplasmic and mitochondrial redox regulatory systems in the yeast *Saccharomyces cerevisiae*. Eukaryot Cell.

[B29] Garrels RM (1960). Mineral Equilibria.

[B30] Dahm LJ, Jones DP (2000). Rat jejunum controls luminal thiol-disulfide redox. J Nutr.

[B31] Trotter EW, Grant CM (2003). Non-reciprocal regulation of the redox state of the glutathione-glutaredoxin and thioredoxin systems. EMBO Rep.

[B32] Drakulic T, Temple MD, Guido R, Jarolim S, Breitenbach M, Attfield PV, Dawes IW (2005). Involvement of oxidative stress response genes in redox homeostasis, the level of reactive oxygen species, and ageing in *Saccharomyces cerevisiae*. FEMS Yeast Res.

[B33] Hanson GT, Aggeler R, Oglesbee D, Cannon M, Capaldi RA, Tsien RY, Remington SJ (2004). Investigating mitochondrial redox potential with redox-sensitive green fluorescent protein indicators. J Biol Chem.

[B34] Macville M, Schröck E, Padilla-Nash H, Keck C, Ghadimi BM, Zimonjic D, Popescu N, Ried T (1999). Comprehensive and definitive molecular cytogenetic characterization of HeLa cells by spectral karyotyping. Cancer Res.

[B35] ATCC (2008). The Global Bioresource Center: Product Description. CRL-1606.

[B36] Mojaverian P (1996). Evaluation of gastrointestinal pH and gastric residence time via the Heidelberg Radiotelemetry Capsule: Pharmaceutical application. Drug Dev Res.

[B37] Imai T, Ohno T (1995). Measurement of yeast intracellular pH by image processing and the change it undergoes during growth phase. J Biotech.

[B38] Llopis J, McCaffery JM, Miyawaki A, Farquhar MG, Tsien RY (1998). Measurement of cytosolic, mitochondrial, and Golgi pH in single living cells with green fluorescent proteins. Proc Natl Acad Sci USA.

[B39] Singh A, Kaur N, Kosman DJ (2007). The metalloreductase Fre6p in Fe-Efflux from the yeast vacuole. J Biol Chem.

[B40] Hansen JM, Go YM, Jones DP (2006). Nuclear and mitochondrial compartmentation of oxidative stress and redox signaling. Annu Rev Pharmacol Toxicol.

[B41] Go YM, Jones DP (2008). Redox compartmentalization in eukaryotic cells. Biochim Biophys Acta-Gen Subj.

[B42] Päuser S, Zschunke A, Khuen A, Keller K (1995). Estimation of water content and water mobility in the nucleus and cytoplasm of *Xenopus laevis *oocytes by NMR spectroscopy. Magn Reson Imaging.

[B43] Botstein D, Amberg D, Mulholland J, Huffaker T, Adams A, Drubin D, Stearns T, Pringle JR, Broach JR, Jones EW (1997). The yeast cytoskeleton. The Molecular and Cellular Biology of the Yeast Saccharomyces: Cell Cycle and Cell Biology.

[B44] Alberts B, Bray D, Lewis J, Raff M, Roberts K, Watson JD (1989). Molecular Biology of the Cell.

[B45] Lew DJ, Weinert T, Pringle JR, Pringle JR, Broach JR, Jones EW (1997). Cell cycle control in *Saccharomyces cerevisiae*. The Molecular and Cellular Biology of the Yeast Saccharomyces: Cell Cycle and Cell Biology.

[B46] Wente SR, Gasser SM, Caplan AJ, Pringle JR, Broach JR, Jones EW (1997). The nucleus and nucleocytoplasmic transport in *Saccharomyces cerevisiae*. The Molecular and Cellular Biology of the Yeast Saccharomyces: Cell Cycle and Cell Biology.

[B47] Kaiser CA, Gimeno RE, Shaywitz DA, Pringle JR, Broach JR, Jones EW (1997). Protein secretion, membrane biogenesis, and endocytosis. The Molecular and Cellular Biology of the Yeast Saccharomyces: Cell Cycle and Cell Biology.

[B48] Jones EW, Webb GC, Hiller MA, Pringle JR, Broach JR, Jones EW (1997). Biogenesis and function of the yeast vacuole. The Molecular and Cellular Biology of the Yeast Saccharomyces: Cell Cycle and Cell Biology.

[B49] Lazarow PB, Kunau W, Pringle JR, Broach JR, Jones EW (1997). Peroxisomes. The Molecular and Cellular Biology of the Yeast Saccharomyces: Cell Cycle and Cell Biology.

[B50] Pon L, Schatz G, Broach JR, Pringle JR, Jones EW (1991). Biogenesis of yeast mitochondria. The Molecular and Cellular Biology of the Yeast Saccharomyces: Genome Dynamics, Protein Synthesis, and Energetics.

[B51] SGD Project (2007). *Saccharomyces *Genome Database. http://www.yeastgenome.org.

[B52] Sarry JE, Chen S, Collum RP, Liang S, Peng M, Lang A, Naumann B, Dzierszinskil F, Yuan CX, Hippler M, Rea PA (2007). Analysis of the vacuolar luminal proteome of *Saccharomyces cerevisiae*. FEBS J.

[B53] Mo CQ, Bard M (2005). Erg28p is a key protein in the yeast sterol biosynthetic enzyme complex. J Lipid Res.

[B54] Tuller T, Kupiec M, Ruppin E (2007). Determinants of protein abundance and translation efficiency in *S. cerevisiae*. PLoS Comput Biol.

[B55] Wicken JS (1987). Evolution, Thermodynamics, and Information.

[B56] Murray DB (2004). On the temporal self-organisation of *Saccharomyces cerevisae*. Curr Genomics.

[B57] Drever JI (1997). The Geochemistry of Natural Waters.

[B58] Hendrickson JB, Cram DJ, Hammond GS (1970). Organic Chemistry.

[B59] Buvet R, Milazzo G, Blank M (1983). General criteria for the fulfillment of redox reactions. Bioelectrochemistry I: Biological Redox Reactions, of Ettore Majorana International Science Series.

[B60] Prigogine I, Defay R (1954). Chemical Thermodynamics.

[B61] Helgeson HC, Kirkham DH (1974). Theoretical prediction of the thermodynamic behavior of aqueous electrolytes at high pressures and temperatures: I. Summary of the thermodynamic/electrostatic properties of the solvent. Am J Sci.

[B62] Wagman DD, Evans WH, Parker VB, Schumm RH, Halow I, Bailey SM, Churney KL, Nuttall RL (1982). The NBS tables of chemical thermodynamic properties. Selected values for inorganic and C_1 _and C_2 _organic substances in SI units. J Phys Chem Ref Data.

[B63] Shock EL, Helgeson HC, Sverjensky DA (1989). Calculation of the thermodynamic and transport properties of aqueous species at high pressures and temperatures: Standard partial molal properties of inorganic neutral species. Geochim Cosmochim Acta.

[B64] Boeckmann B, Bairoch A, Apweiler R, Blatter MC, Estreicher A, Gasteiger E, Martin MJ, Michoud K, O'Donovan C, Phan I, Pilbout S, Schneider M (2003). The SWISS-PROT protein knowledgebase and its supplement TrEMBL in 2003. Nucleic Acids Res.

[B65] Shock EL, Sassani DC, Willis M, Sverjensky DA (1997). Inorganic species in geologic fluids: Correlations among standard molal thermodynamic properties of aqueous ions and hydroxide complexes. Geochim Cosmochim Acta.

[B66] Chan P, Lovrić J, Warwicker J (2006). Subcellular pH and predicted pH-dependent features of proteins. Proteomics.

[B67] Wu MM, Llopis J, Adams S, McCaffery JM, Kulomaa MS, Machen TE, Moore HPH, Tsien RY (2000). Organelle pH studies using targeted avidin and fluorescein-biotin. Chem Biol.

[B68] Welch MD, Iwamatsu A, Mitchison TJ (1997). Actin polymerization is induced by Arp2/3 protein complex at the surface of *Listeria monocytogenes*. Nature.

[B69] Mullins RD, Pollard TD (1999). Structure and function of the Arp2/3 complex. Curr Opin Struct Biol.

[B70] Schmid M, Jaedicke A, Du TG, Jansen RP (2006). Coordination of endoplasmic reticulum and mRNA localization to the yeast bud. Curr Biol.

[B71] Frazier JA, Wong ML, Longtine MS, Pringle JR, Mann M, Mitchison TJ, Field C (1998). Polymerization of purified yeast septins: Evidence that organized filament arrays may not be required for septin function. J Cell Biol.

[B72] Burri L, Lithgow T (2004). A complete set of SNAREs in yeast. Traffic.

[B73] Kostelansky MS, Schluter C, Tam YYC, Lee S, Ghirlando R, Beach B, Conibear E, Hurley JH (2007). Molecular architecture and functional model of the complete yeast ESCRT-I heterotetramer. Cell.

[B74] Hierro A, Sun J, Rusnak AS, Kim J, Prag G, Emr SD, Hurley JH (2004). Structure of the ESCRT-II endosomal trafficking complex. Nature.

[B75] Conibear E, Cleck JN, Stevens TH (2003). Vps51p mediates the association of the GARP (Vps52/53/54) complex with the late Golgi t-SNARE Tlg1p. Mol Biol Cell.

[B76] Miranda JJL, De Wulf P, Sorger PK, Harrison SC (2005). The yeast DASH complex forms closed rings on microtubules. Nat Struct Mol Biol.

[B77] Rout MP, Aitchison JD, Suprapto A, Hjertaas K, Zhao YM, Chait BT (2000). The yeast nuclear pore complex: Composition, architecture, and transport mechanism. J Cell Biol.

[B78] Bernstein KA, Gallagher JEG, Mitchell BM, Granneman S, Baserga SJ (2004). The small-subunit processome is a ribosome assembly intermediate. Eukaryot Cell.

[B79] Vinh DBN, Kern JW, Hancock WO, Howard J, Davis TN (2002). Reconstitution and characterization of budding yeast *γ*-tubulin complex. Mol Biol Cell.

[B80] Gavin AC, Aloy P, Grandi P, Krause R, Boesche M, Marzioch M, Rau C, Jensen LJ, Bastuck S, Dumpelfeld B, Edelmann A, Heurtier MA, Hoffman V, Hoefert C, Klein K, Hudak M, Michon AM, Schelder M, Schirle M, Remor M, Rudi T, Hooper S, Bauer A, Bouwmeester T, Casari G, Drewes G, Neubauer G, Rick JM, Kuster B, Bork P, Russell RB, Superti-Furga G (2006). Proteome survey reveals modularity of the yeast cell machinery. Nature.

