# Supplementary material for: Calculation of the relative metastabilities of proteins in subcellular compartments of Saccharomyces cerevisiae
Source: BMC Syst Biol. 2009 Jul 18;3:75. doi: 10.1186/1752-0509-3-75 (PMC2734844; doi:10.1186/1752-0509-3-75)
Supplement: Additional file 3 — Interactions between subcellular compartments in yeast. This file lists statements from Refs. [43,47,46,50] used to identify the interactions between proteins in different compartments of Saccharomyces cerevisiae that are listed in Table 4. [file 1752-0509-3-75-S3.pdf]

Additional File 3: Statements from the literature regarding protein interactions in *Saccharomyces cerevisiae*

Statements implying interaction between proteins in different compartments were identified by scanning for action words including interact, are at, align, end at, organize, embed, move, associate, found, locate, extend, bisect, move, migrate, enter, attach, translocate, carry, sort, composed of, line, dock and fuse, recycle, transport, pinch, proceed, reach, degrade in, deliver, colocalize, contain, associate, separate, protrude, penetrate, cooperate, crosstalk, anchor, reside, continuous with, shuttle, oxidize, essential to, convey, arrange, import, and transcribe.

The sections below correspond to different review articles surveyed. Following each statement (action words highlighted here in bold type), a summary of the interaction is given, e.g. *microtubule–spindle.pole*. Negative associations are indicated by e.g. *actin/microtubule*.

Cytoskeleton [Botstein et al., 1997]

- The two major types of actin structures are (1) patches at the periphery, or cortex, of the cell and (2) cables, also **aligned** along the cortex of the cell ... (p. 3)

  actin–cell.periphery

- All microtubules in yeast have one end at the spindle pole body (SPB), a microtubule-**organizing** center that is **embedded** in the nuclear envelope, which does not break down during mitosis in fungi. (p. 3-4)

  microtubule–spindle.pole

  spindle.pole–nuclear.envelope

- The functions of the microtubule cytoskeleton appear to be limited to processes involving movement of the chromosomes (mitosis and meiosis and spindle orientation) and **movements** of the nucleus during the cell cycle (nuclear migration) and after mating (karyogamy). (p. 4)

  microtubule–nucleus

- It should be pointed out that calmodulin is one of the points of proven **interaction** between the actin and tubulin cytoskeletons. (p. 14)

  actin–microtubule

- The [actin] patches are seen as electron-dense regions almost always **associated** with invaginations of the plasma membrane. (p. 14)

  actin–cell.periphery

- When the bud first emerges, the ring of patches **remains** as a collar at its base ... disappearing as the bud enlarges. (p. 22-23)

  actin–bud.neck

- In cells with small buds, actin patches are **found** concentrated in the developing bud. (p. 23)

  actin–bud

- The [actin] ring ... is similar in **location** to the septin rings found at the neck. (p. 23)

  actin–bud.neck

- Recent advances in fluorescence microscopy have made it clear that most of the [actin] fibers run along the inner surface of cells and that very few, if any, **extend** any distance into the cytoplasm. (p. 24)

  actin/cytoplasm

- Cytoplasmic microtubules [**colocation** inferred] are required for both of these [nuclear division] processes. (p. 39)

  cytoplasm–microtubule

- Thus, nuclear positioning is another point at which the actin and microtubule cytoskeletons functionally **interact**. (p. 39)

  actin–nucleus–microtubule

- Covisualization of actin cables and mitochondria in wild-type yeast cell has shown that mitochondria are **organized** along actin cables and that this organization is disrupted in certain actin mutants. (p. 43)

  actin–mitochondria

- Byers and Goetsch found two classes of microtubules in yeast: those that **extend** into the nucleus, which we refer to as intranuclear microtubules, and those that **extend** outward from the nuclear membrane, which we refer to as cytoplasmic microtubules. (p. 47)

  microtubule–cytoplasm

  microtubule–nucleus

- Both intranuclear and cytoplasmic microtubules originate at a structure **embedded** in the nuclear membrane called the spindle pole body ... (p. 47)

  microtubule–spindle.pole

- The mitotic spindle can be observed by DIC microscopy ... Under optimal conditions, the spindle is visible as a bar **bisecting** the nucleus. (p. 47)

  spindle.pole–nucleus

- It appears that a set of **movements** of the nucleus within the cytoplasm of the mother function to position the spindle properly. (p. 53)

  nucleus–cytoplasm

- After SPB duplication and before the onset of spindle elongation, the nucleus **migrates** to the neck, typically remaining on the mother side of the junction between mother and bud. (p. 53)

  nucleus–bud.neck

- In most cases [of mitosis], the cytoplasmic microtubules from the budward SPB **enter** the bud, often extending all the way to the tip of the bud ... (p. 54)

  microtubule–bud

- In many immunofluorescence images, it appears that the cytoplasmic microtubules **interact** with the cortex of the cell, particularly in the bud. (p. 54)

  microtubule–cell.periphery

- Like the centrosome, it [spindle pole body] **occupies** a perinuclear location in the cell ... (p. 73)

  spindle.pole–nuclear.periphery

- The SPB is a trilaminate structure with a central plaque **embedded** in the nuclear envelope and inner and outer plaques attached to the central plaque. The inner plaque is within the nucleus and the outer plaque is in the cytoplasm. (p. 73)

  nucleus–spindle.pole–cytoplasm

- All microtubules in the cell appear to be **attached** to the SPB throughout the cell cycle. (p. 73)

  microtubule–spindle.pole

Protein Secretion [Kaiser et al., 1997]

- Secretory proteins are **translocated** from the cytosol into the lumen of the endoplasmic reticulum (ER). (p. 93)

  cytoplasm–ER

- These vesicles [budding from the ER] then **deliver** their contents to the Golgi by fusion with the Golgi membrane ... The Golgi apparatus is composed of three biochemically distinguishable compartments. Secretory proteins are **carried** through successive compartments of the Golgi probably by more rounds of vesicle formation and fusion. (p. 94)

  ER–early.Golgi

  early.Golgi–Golgi

  Golgi–late.Golgi

- Resident ER proteins that have **reached** an early Golgi compartment are returned to the ER by retrograde vesicles that bud from the Golgi and fuse with the membrane of the ER. (p. 94)

  ER–early.Golgi

- Clearly, there are fundamental differences between the behavior of soluble and membrane proteins in the secretory pathway. A prominent example being the **sorting** of proteins to the vacuole and to the plasma membrane that occurs in a late Golgi compartment. (p. 96)

  late.Golgi–vacuole

- Localization of the ER proteins ... shows that the ER is **composed** of the nuclear envelope and a single discontinuous cisternae that lines the inner surface of the plasma membrane. (p. 97)

  ER–nuclear.periphery

  ER–cell.periphery

- Vesicles that have successfully budded from the ER must next **dock** and **fuse** with the appropriate target membrane, the *cis*-face of the Golgi. (p. 131)

  ER–early.Golgi

- In mammalian cells, soluble vacuolar proteins and their receptors are transported together to a prevacuolar/late endosomal sorting compartment. In this compartment, the receptor **recycles** back to the Golgi complex, and the cargo protein is **transported** to the vacuole. (p. 159)

  endosome–vacuole

  endosome–Golgi

- Possibly, the actin cytoskeleton provides the mechanical force necessary for the formation or **pinching** off of endocytic vesicles. (p. 183)

  endosome–actin

Vacuole [Jones et al., 1997]

- Some membrane proteins **proceed** there [vacoule] directly from their normal locations in the Golgi cisternae, presumably by a normal delivery pathway, others from the plasma membrane via (often selective) endocytosis, and still others via autophagy. (p. 365)

  vacuole–Golgi

  vacuole–endosome

  vacuole–cell.periphery

- Most vacuolar enzymes are synthesized as inactive precursors, whether they **reach** the vacuole via the secretory pathway or the extra-secretory cytoplasm-to-vacuole pathway. (p. 365)

  vacuole–cytoplasm

- Mutant forms of the plasma membrane [proteins] ... are both **delivered** directly to the vacuole and degrade there. (p. 391)

  vacuole–cell.periphery

- **Sorting** of both soluble and membrane vacuolar hydrolase precursors appears to take place in a late Golgi compartment. (p. 409)

  vacuole–late.Golgi

- ... evidence that an endosomal compartment is present **between** the Golgi complex and the vacuole. (p. 415)

  vacuole–endosome–Golgi

- This compartment [endosome] is kinetically **located** after the Golgi complex but before the vacuole. (p. 421)

  vacuole–endosome–Golgi

- Small vacuoles **move** into the bud, followed by fusion and expansion of the vacuole as cell density decreases. (p. 443)

  vacuole–bud

- ... actin cables appear to **colocalize** with vacuolar membranes ... (p. 444)

  vacuolar.membrane–actin

Nucleus [Wente et al., 1997]

- There are at least two functional compartments within the nucleus, one containing the majority of the chromatin ... and one **containing** the nucleolus ... (p. 473)

  nucleus–nucleolus

- ... [nucleolus] **occupies** one side of the nucleus and associates closely with the nuclear envelope. (p. 474)

  nucleolus–nuclear.periphery

- The cytoplasm and nucleoplasm are **separated** by the two lipid bilayer membranes of the nuclear envelope ... (p. 474)

  nuclear.periphery–cytoplasm

  nuclear.periphery–nucleus

- The outer nuclear membrane is **continuous** with the endoplasmic reticulum (ER), so that the intermembrane space is continuous with the lumen of the ER. (p. 474)

  nuclear.periphery–ER

- As the newly formed bud enlarges, the nucleus migrates to a position proximal to the bud site ... This is followed by **extension** of the nucleus into the bud, which involves a very elongated **protrusion** of the nuclear membrane through the mother-bud neck. (p. 487)

  nucleus–bud

  nuclear.periphery–bud.neck

- However, once the nucleus has **migrated** to the bud neck, it can be stably maintained in that position independent of microtubule function. (p. 487)

  nucleus–bud.neck

- During or after nuclear migration, the spindle becomes oriented such that one pole **penetrates** the mother-bud neck prior to anaphase ... (p. 487)

  microtubule–bud.neck

- These data suggest **cooperation** or **crosstalk** between the actin- and microtubule-based cytoskeletal networks during nuclear movement and spindle orientation. (p. 489)

  actin–nucleus–microtubule

- Since actin is polarized in the bud, it seems likely that microtubule-based nuclear movement is given directionality by **anchoring** to actin-based structures residing in the bud itself. (p. 489)

  bud–actin–microtubule

- ... distinct cytosolic components facilitate **docking** at the nuclear envelope ... (p. 509)

  nuclear.periphery–cytoplasm

- ... it [specific protein] **shuttles** rapidly between the nucleus and the cytoplasm. (p. 523)

  nucleus–cytoplasm

Peroxisome [Lazarow and Kunau, 1997]

- A second major peroxisomal function is the β-**oxidation** of fatty acids ... (p. 548)

  peroxisome–lipid.particle

- ... [peroxisomes] are often **found** at the periphery of the cell ... (p. 551)

  peroxisome–cell.periphery

- These peroxisomes are often found in clusters, sometimes **near** the cell surface but also throughout the cytoplasm. (p. 553)

  peroxisome–cytoplasm

- Deletion of [gene for specific peroxisomal protein] causes massive accumulation of fat droplets ... indicating that [specific peroxisomal protein] is **essential** for fatty acid metabolism ... (p. 578)

  peroxisome–lipid.particle

- Phospholipids ... are synthesized in the endoplasmic reticulum and in mitochondria and **conveyed** to peroxisomes by an unknown mechanism. (p. 582)

  peroxisome–ER

  peroxisome–mitochondria

Mitochondria [Pon and Schatz, 1991]

- As the cells switched from glucose fermentation to ethanol oxidation during early stationary phase ... the mitochondrial reticulum broke up into many small, regular mitochondria that were preferentially **arranged** at the periphery of the cell. (p. 336)

  mitochondria–cell.periphery

- **Transcription** of mitochondrial genes appears to be catalyzed by a nucleus-encoded RNA polymerase ... (p. 347)

  mitochondria–nucleus

- All of the nucleus-encoded proteins and many of the small molecules are made in the cytosol and subsequently **imported** into the mitochondria. (p. 357)

  mitochondria–cytoplasm

References

D. Botstein, D. Amberg, J. Mulholland, T. Huffaker, A. Adams, D. Drubin, and T. Stearns. The yeast cytoskeleton. In J. R. Pringle, J. R. Broach, and E. W. Jones, editors, *The Molecular and Cellular Biology of the Yeast* Saccharomyces*: Cell Cycle and Cell Biology*, pages 1 – 90. Cold Spring Harbor Laboratory Press, New York, 1997.

E. W. Jones, G. C. Webb, and M. A. Hiller. Biogenesis and function of the yeast vacuole. In J. R. Pringle, J. R. Broach, and E. W. Jones, editors, *The Molecular and Cellular Biology of the Yeast* Saccharomyces*: Cell Cycle and Cell Biology*, pages 363 – 470. Cold Spring Harbor Laboratory Press, New York, 1997.

C. A. Kaiser, R. E. Gimeno, and D. A. Shaywitz. Protein secretion, membrane biogenesis, and endocytosis. In J. R. Pringle, J. R. Broach, and E. W. Jones, editors, *The Molecular and Cellular Biology of the Yeast* Saccharomyces*: Cell Cycle and Cell Biology*, pages 91 – 227. Cold Spring Harbor Laboratory Press, New York, 1997.

P. B. Lazarow and W. Kunau. Peroxisomes. In J. R. Pringle, J. R. Broach, and E. W. Jones, editors, *The Molecular and Cellular Biology of the Yeast* Saccharomyces*: Cell Cycle and Cell Biology*, pages 547 – 605. Cold Spring Harbor Laboratory Press, New York, 1997.

L. Pon and G. Schatz. Biogenesis of yeast mitochondria. In J. R. Broach, J. R. Pringle, and E. W. Jones, editors, *The Molecular and Cellular Biology of the Yeast* Saccharomyces*: Genome Dynamics, Protein Synthesis, and Energetics*, pages 333 – 406. Cold Spring Harbor Laboratory Press, New York, 1991.

S. R. Wente, S. M. Gasser, and A. J. Caplan. The nucleus and nucleocytoplasmic transport in *Saccharomyces cerevisiae*. In J. R. Pringle, J. R. Broach, and E. W. Jones, editors, *The Molecular and Cellular Biology of the Yeast* Saccharomyces*: Cell Cycle and Cell Biology*, pages 471 – 546. Cold Spring Harbor Laboratory Press, New York, 1997.
