# Supplementary material for: Calculation of the relative metastabilities of proteins in subcellular compartments of Saccharomyces cerevisiae
Source: BMC Syst Biol. 2009 Jul 18;3:75. doi: 10.1186/1752-0509-3-75 (PMC2734844; doi:10.1186/1752-0509-3-75)
Supplement: Additional file 6 — Abundance comparison for model proteins for compartments and complexes. Scatterplots of experimental vs. calculated logarithm of activity of model proteins in subcellular compartments were generated for a range of logarithm of oxygen fugacity from -82 to -70.5. The legend of each diagram indicates the logarithm of oxygen fugacity ("O2"in the legend), root mean square deviation ("rmsd" in the legend; RMSD in Eqn. 7) and the Spearman rank correlation coefficient ("rr" in the legend; ρ in Eqn. 8). [file 1752-0509-3-75-S6.pdf]

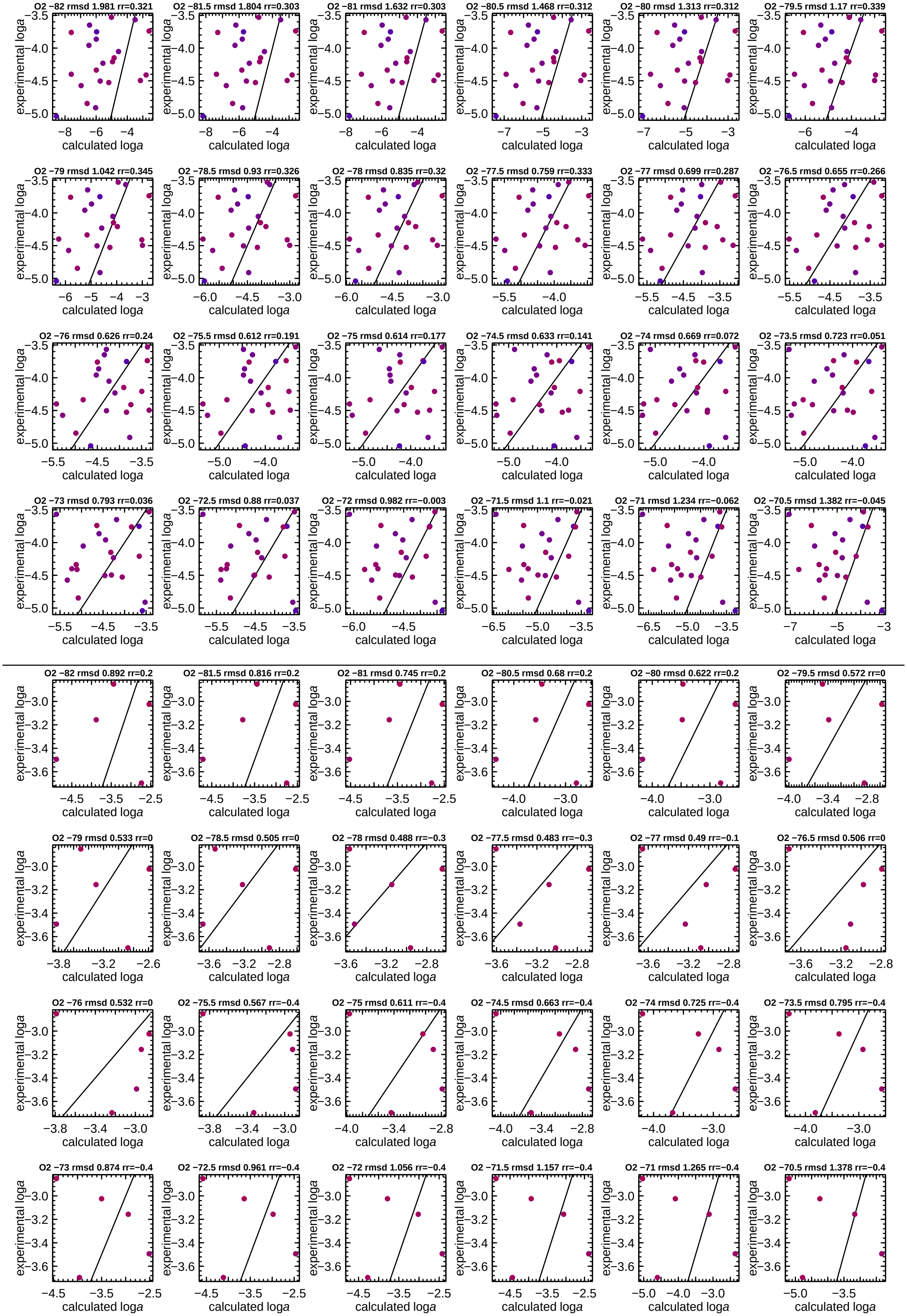

Figure S6-i: actin (top); Arp2/3 complex (bottom)

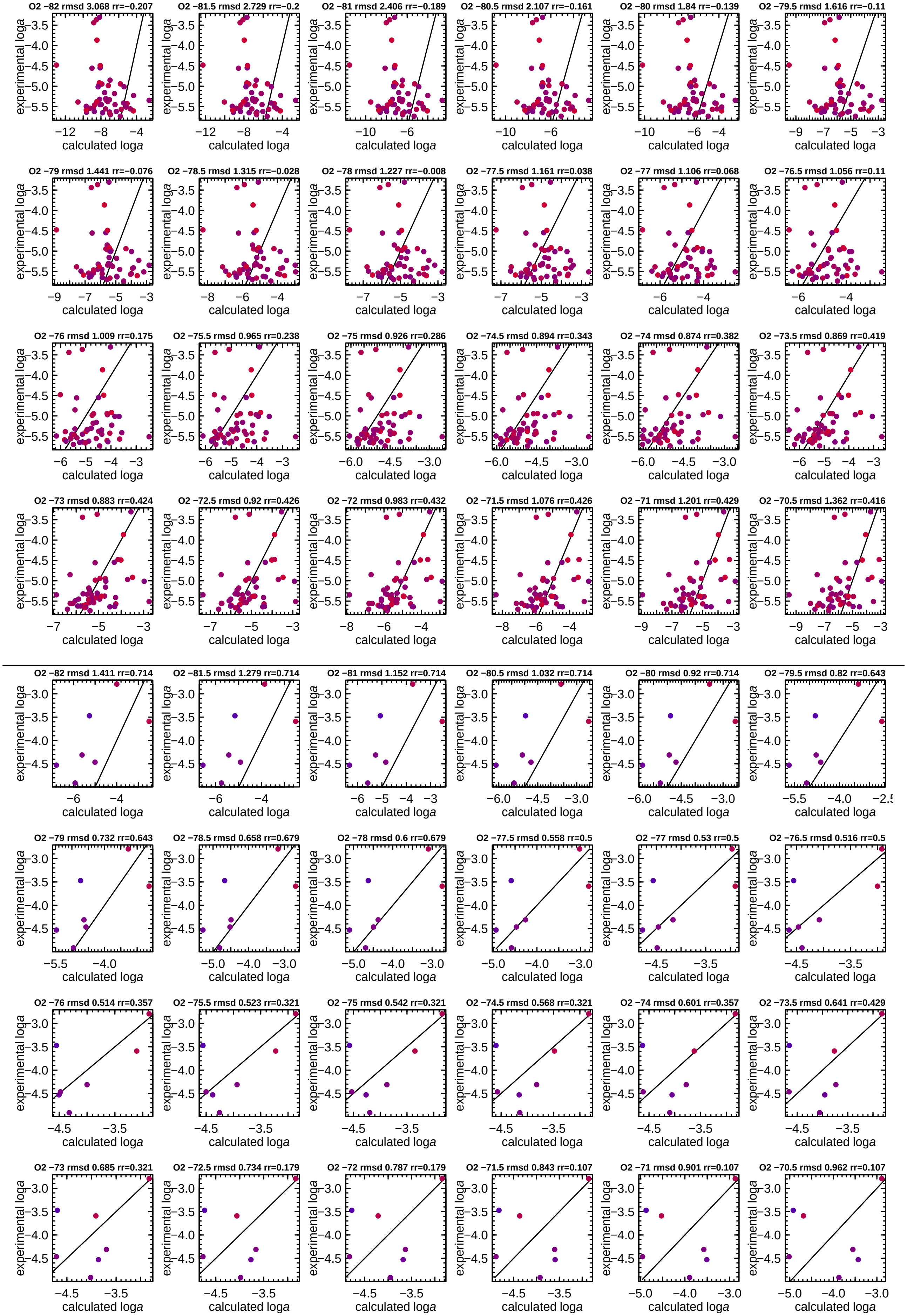

Figure S6-ii: ambiguous (top); cyclin-dependent protein kinase complex (bottom)

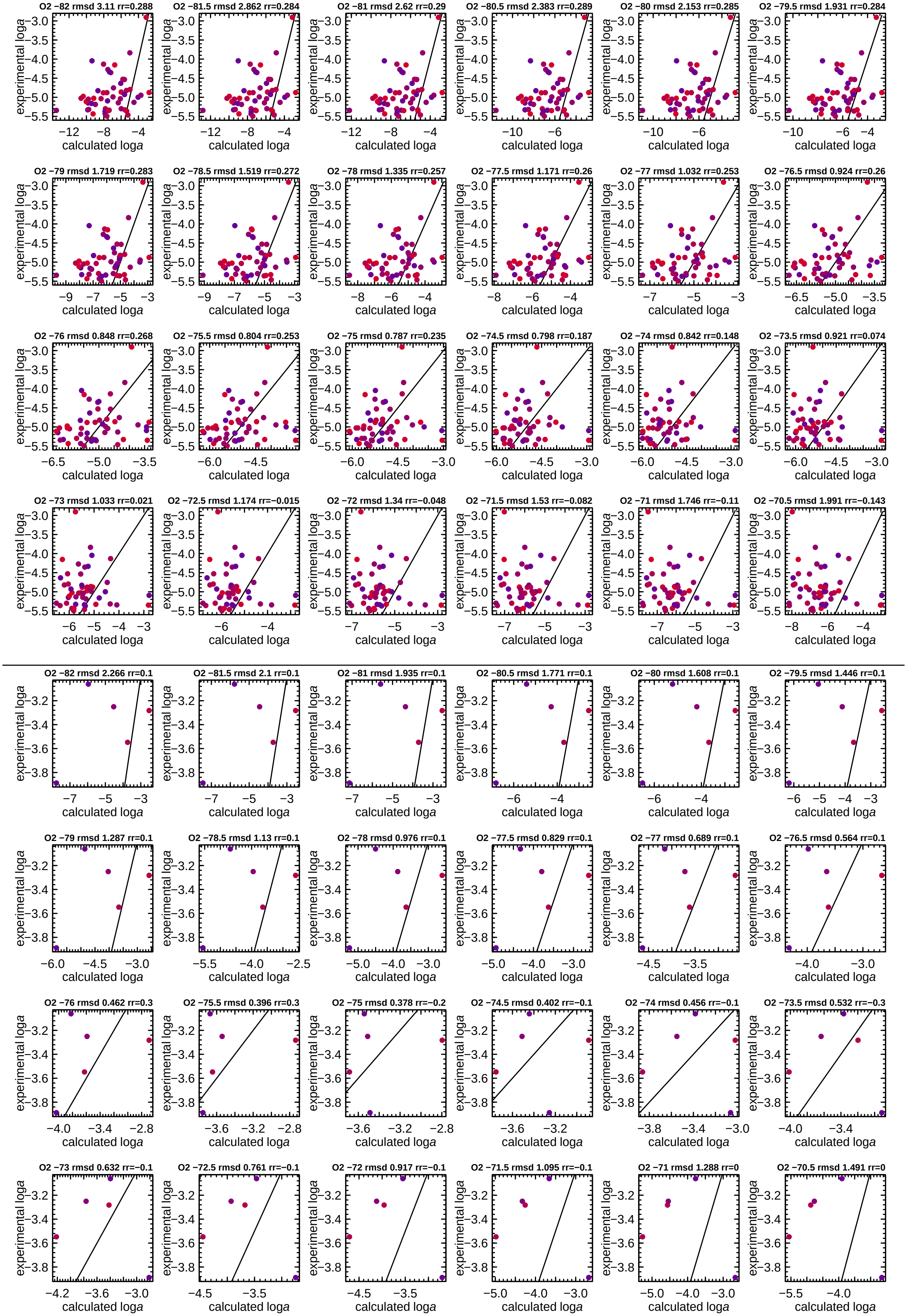

Figure S6-iii: bud (top); actin-associated motor complex (bottom)

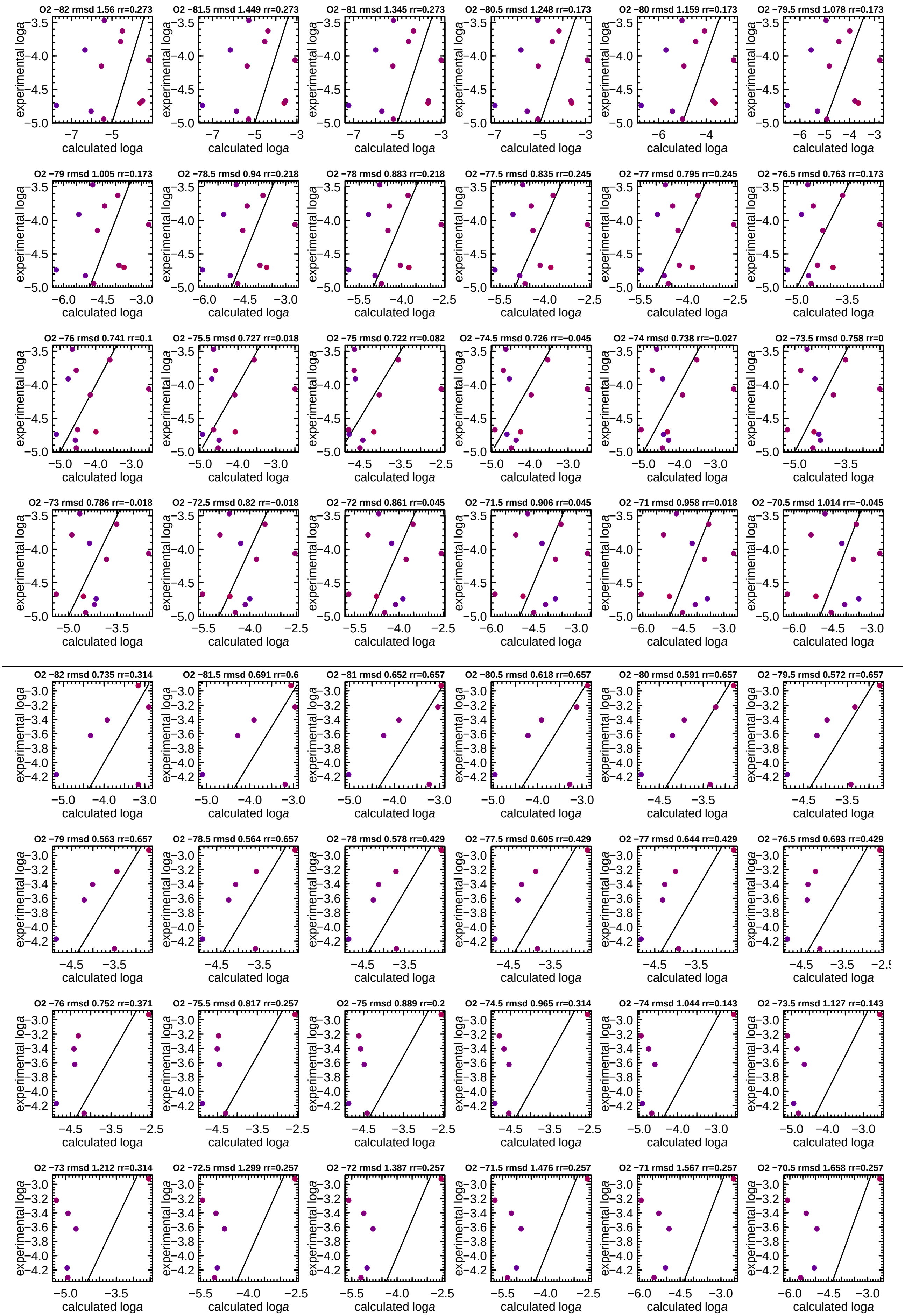

Figure S6-iv: bud.neck (top); septin complex (bottom)

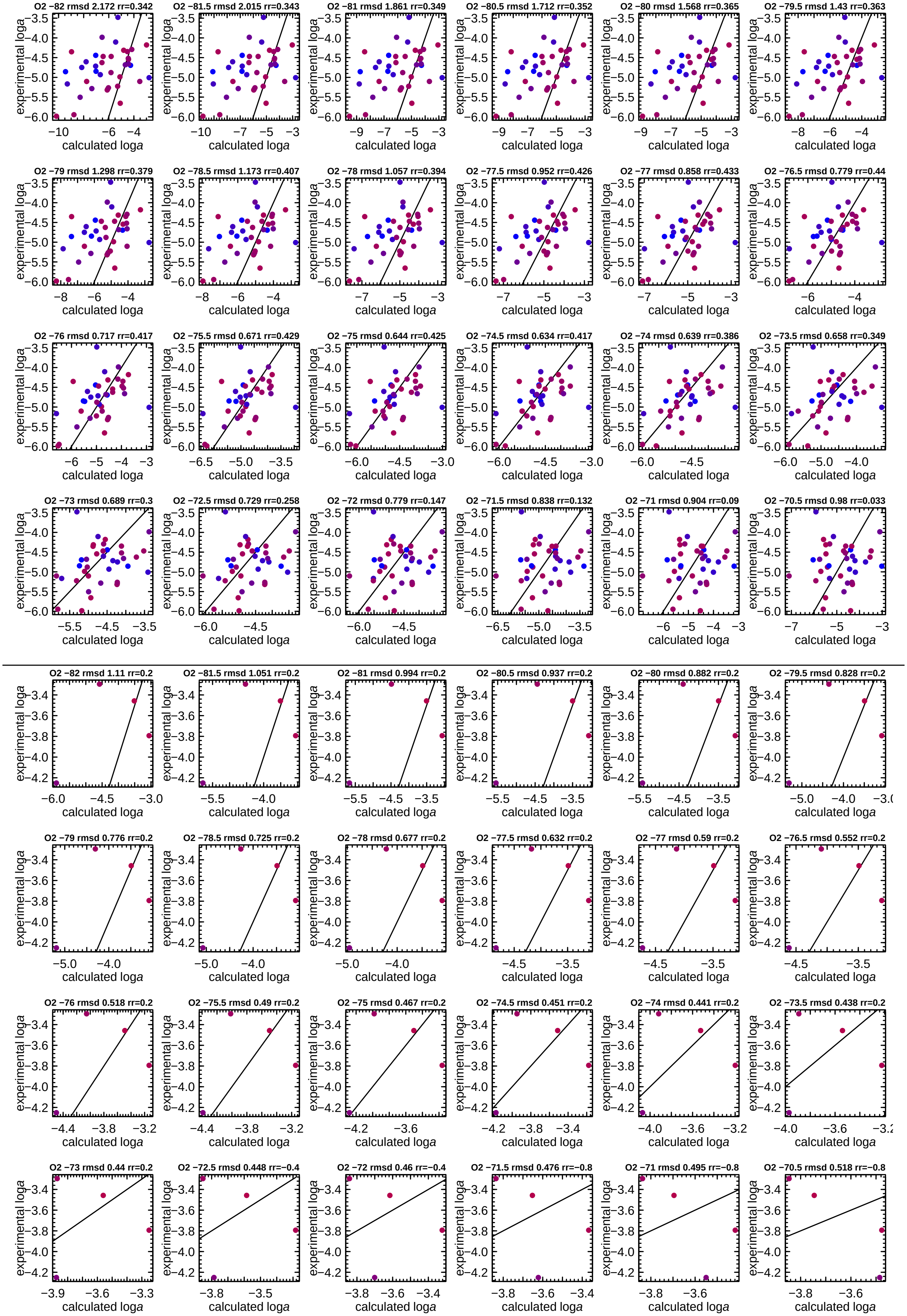

Figure S6-v: cell.periphery (top); exocyst complex (bottom)

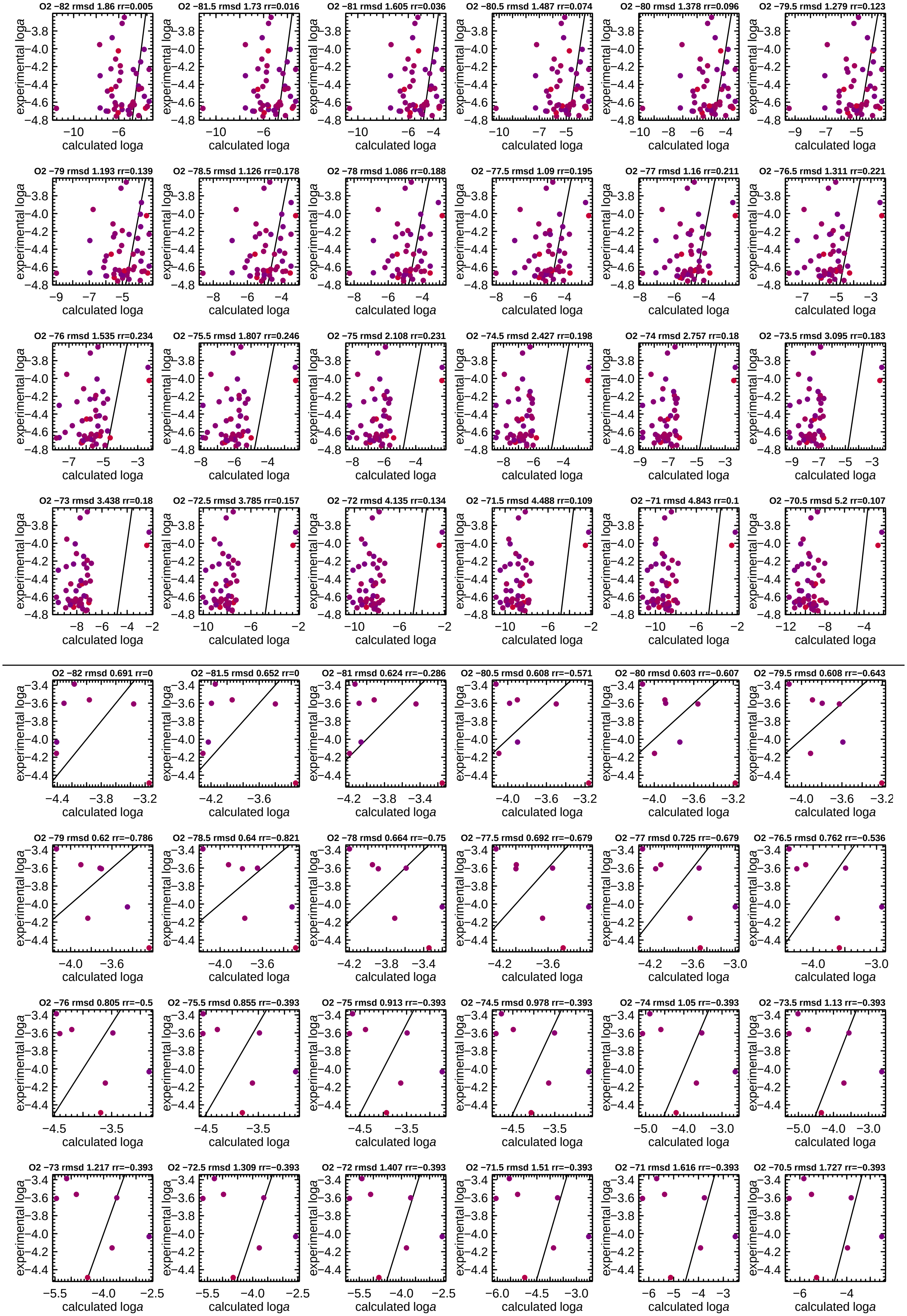

Figure S6-vi: cytoplasm (top); translation initiation factor eIF3 (bottom)

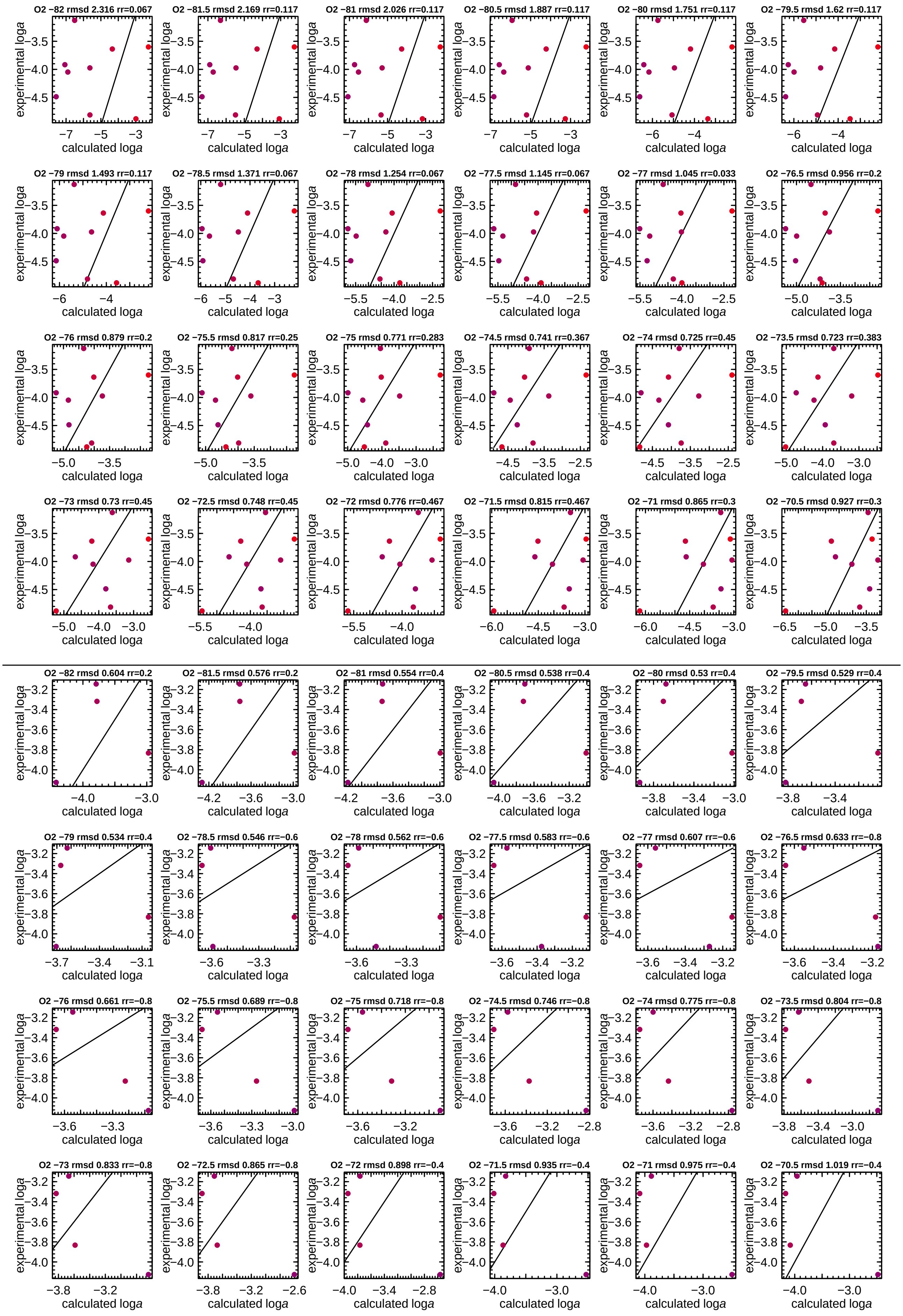

Figure S6-vii: early.Golgi (top); SNARE complex (bottom)

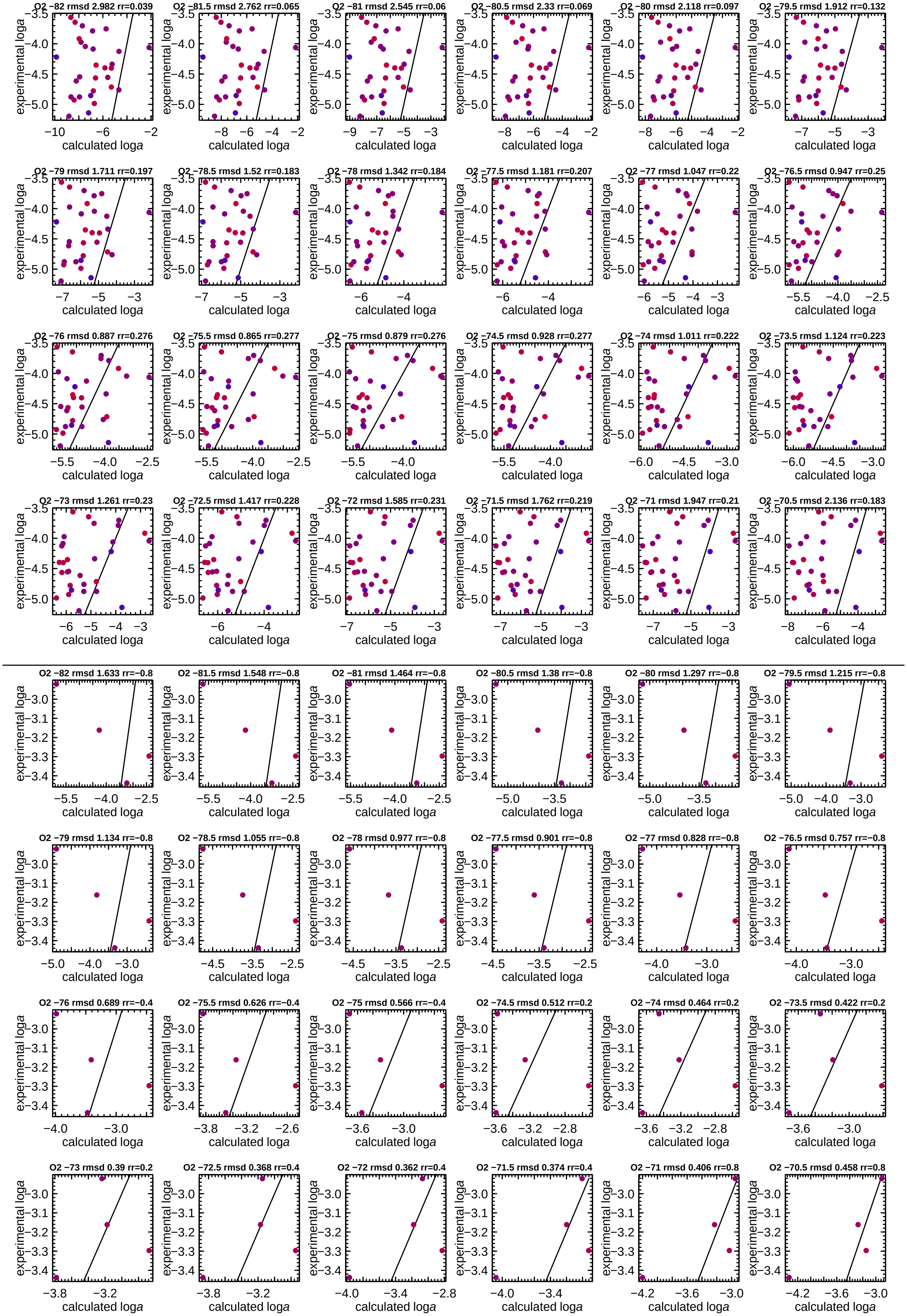

Figure S6-viii: endosome (top); ESCRT I & II complexes (bottom)

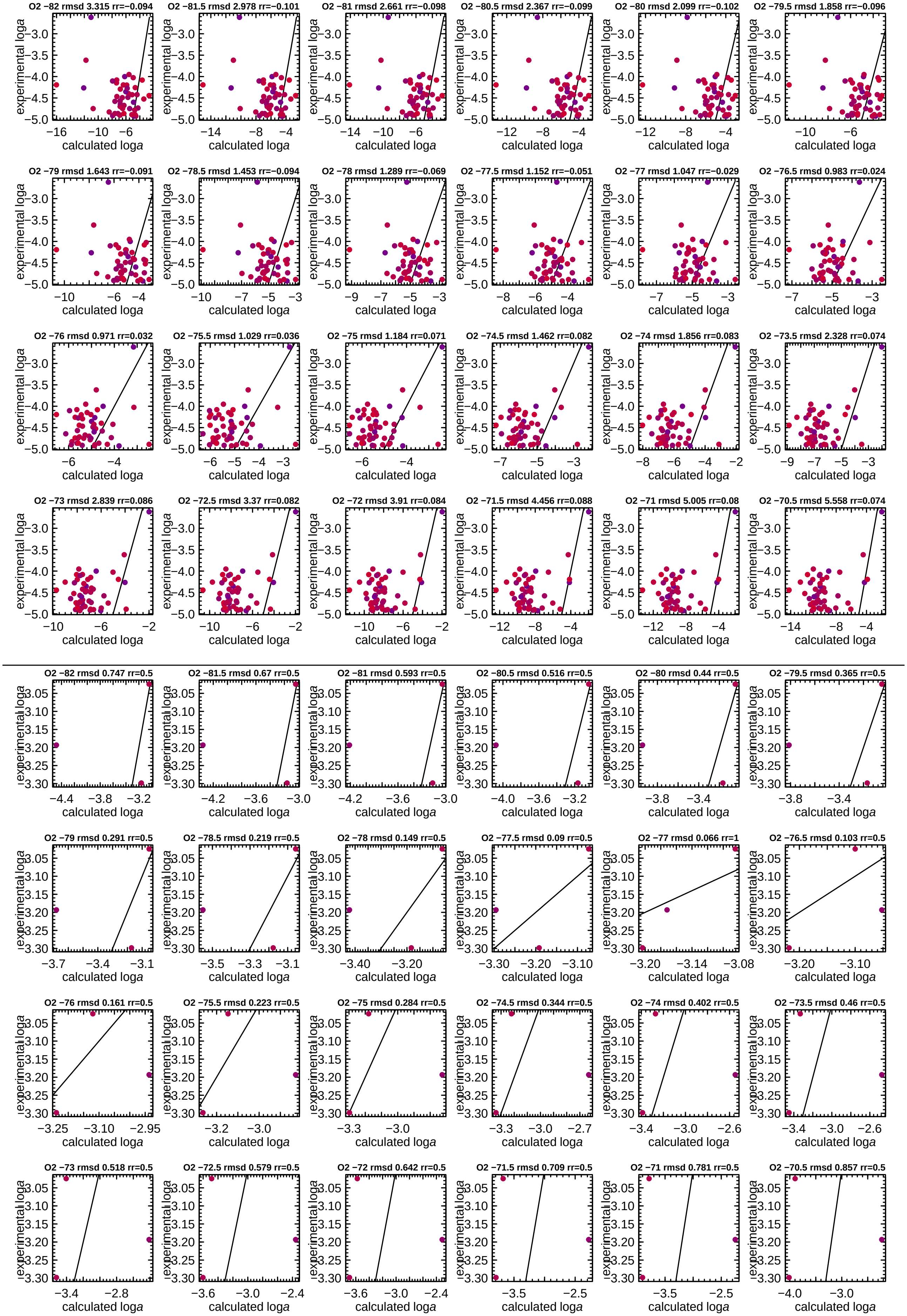

Figure S6-ix: ER (top); signal recognition complex (bottom)

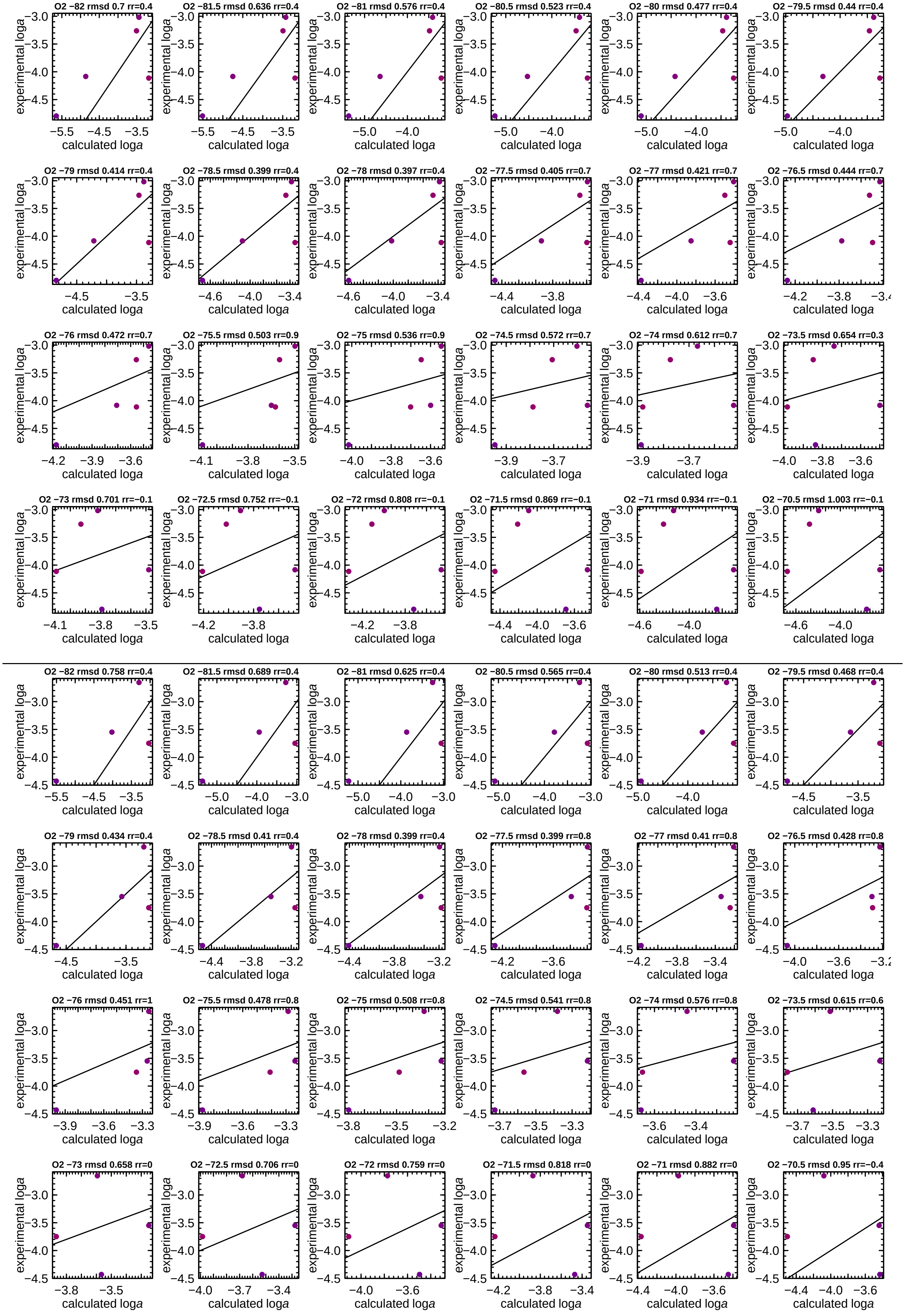

Figure S6-x: ER.to.Golgi (top); coatomer COPII complex (bottom)

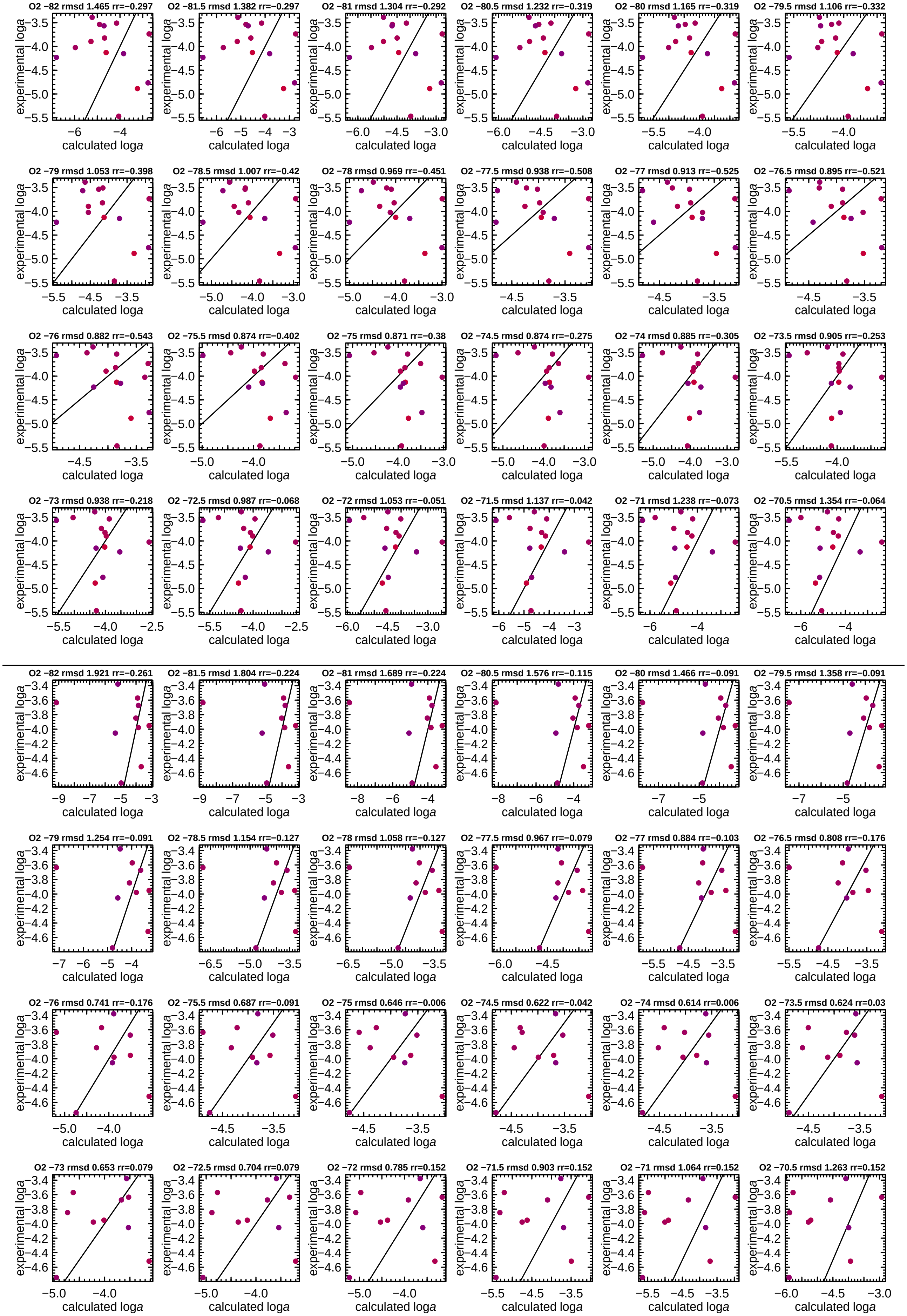

Figure S6-xi: Golgi (top); Golgi transport complex (bottom)

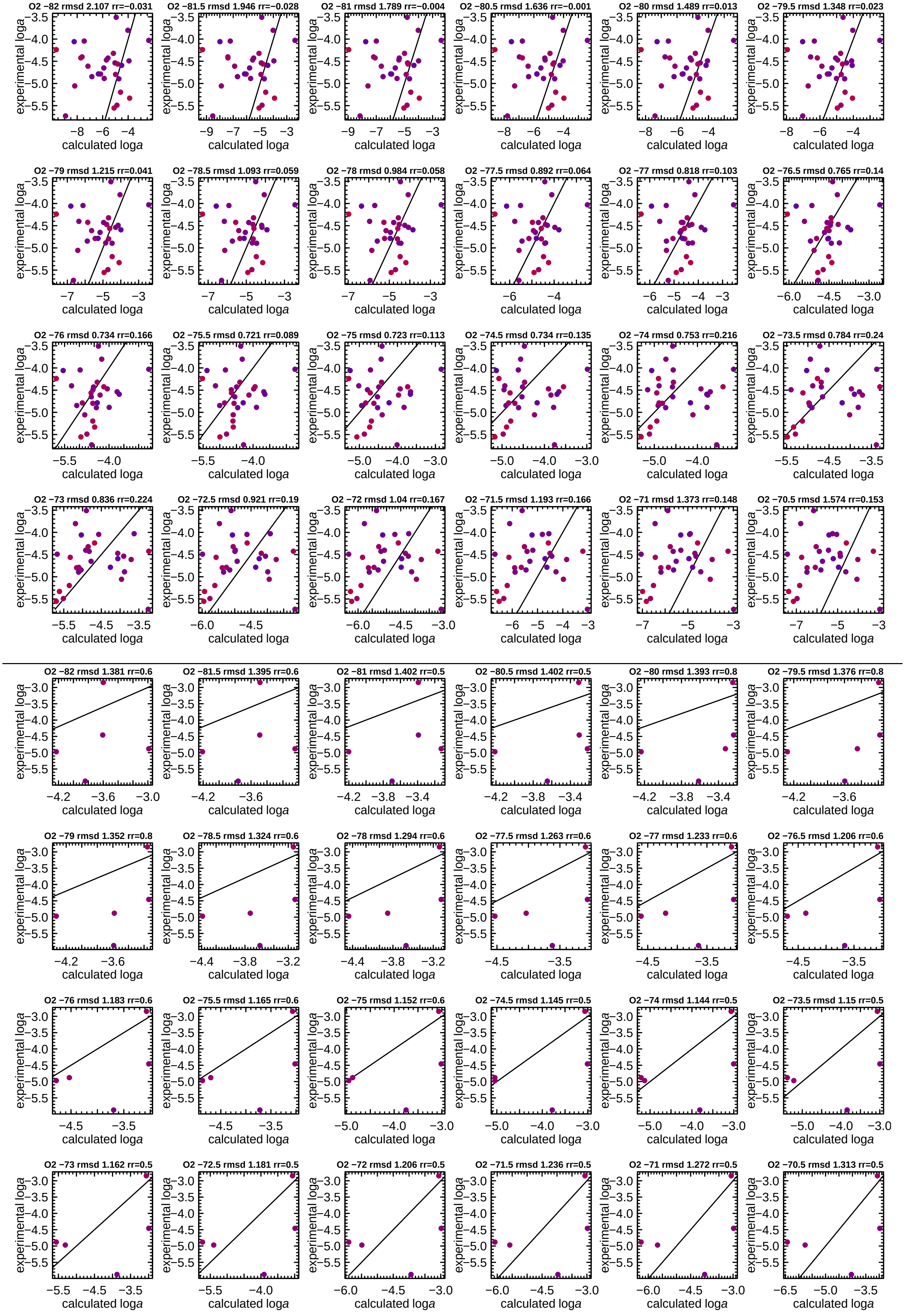

Figure S6-xii: late.Golgi (top); retrograde protein complex (bottom)

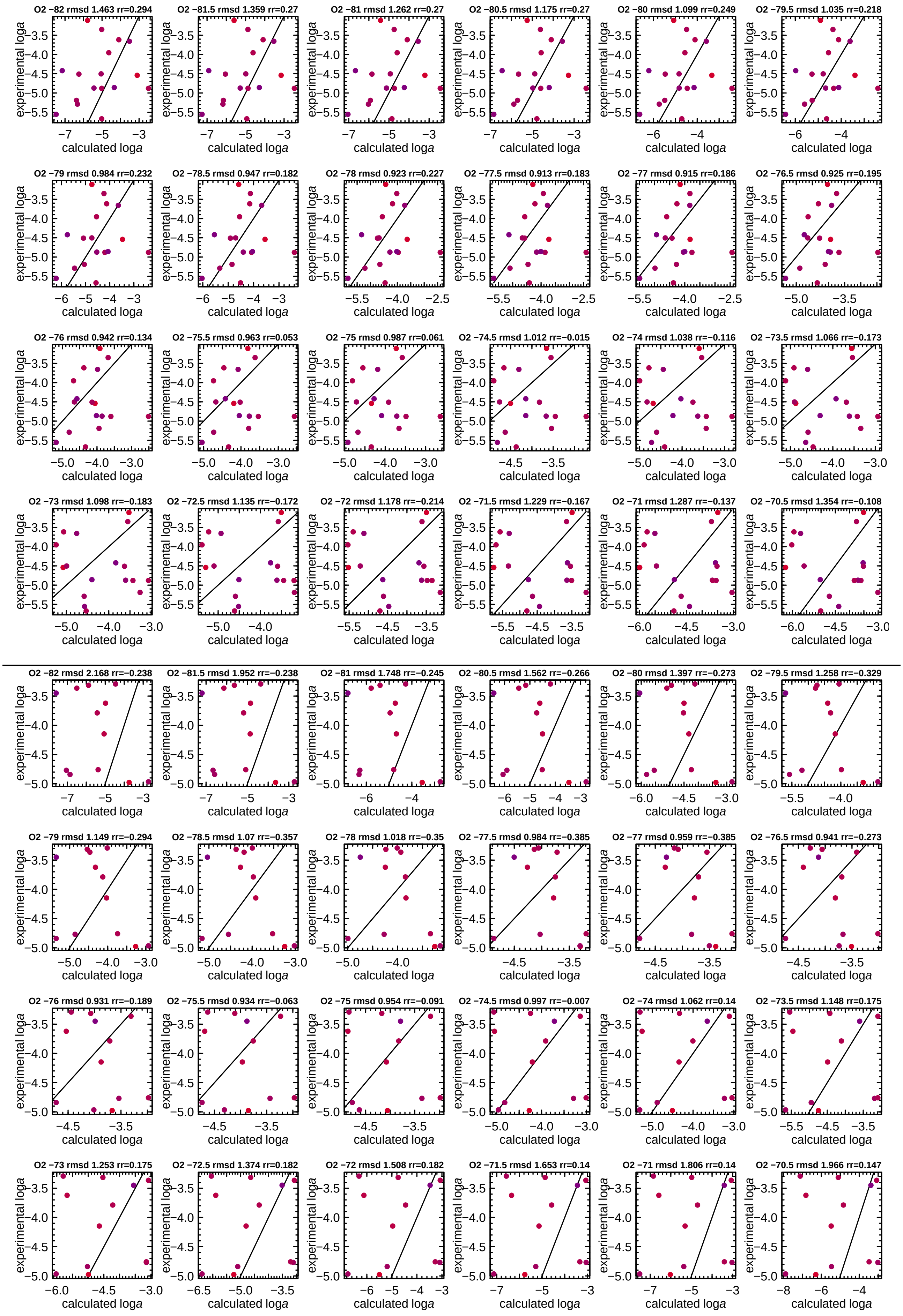

Figure S6-xiii: lipid.particle (top); sterol biosynthesis enzymes (bottom)

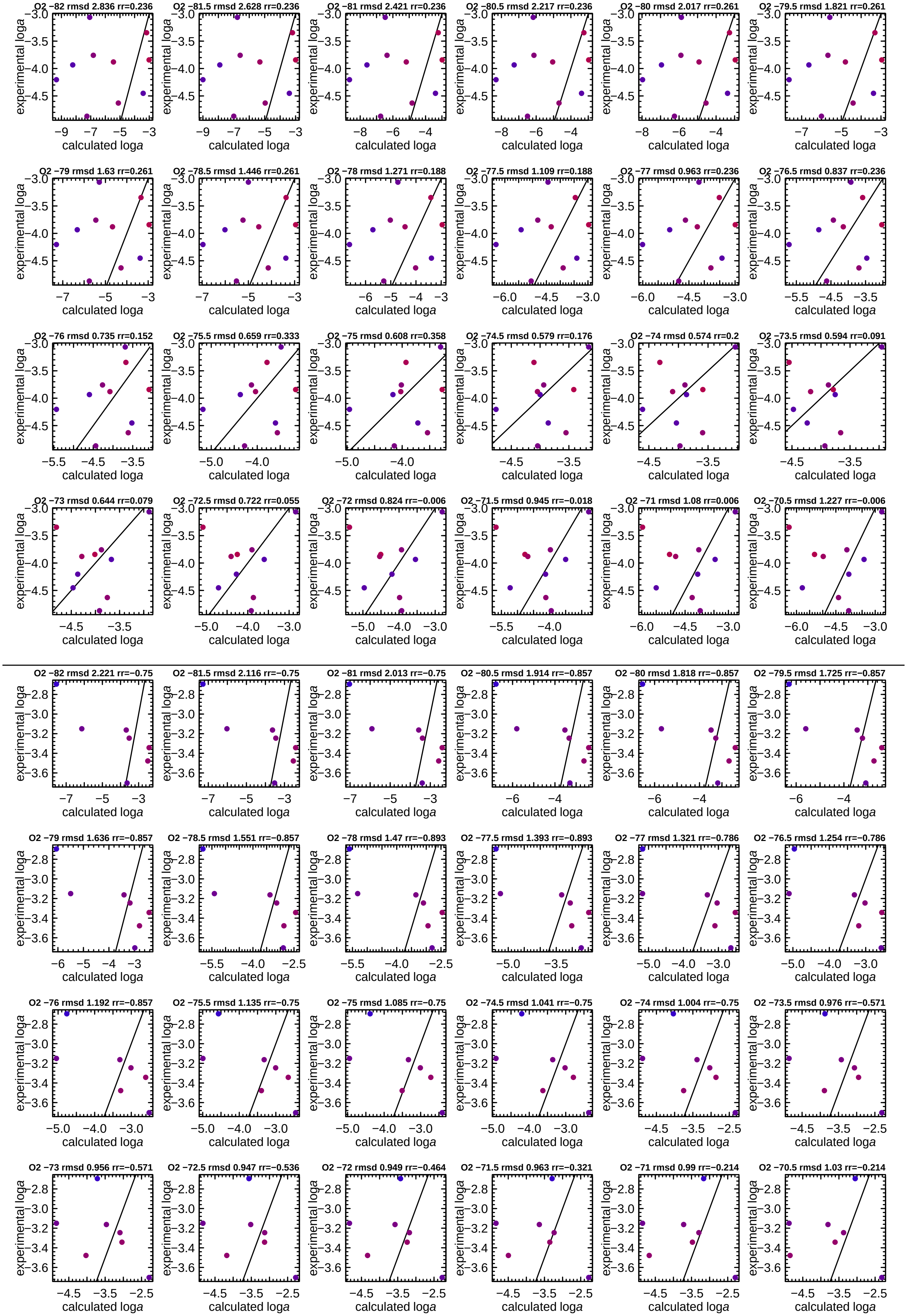

Figure S6-xiv: microtubule (top); DASH complex (bottom)

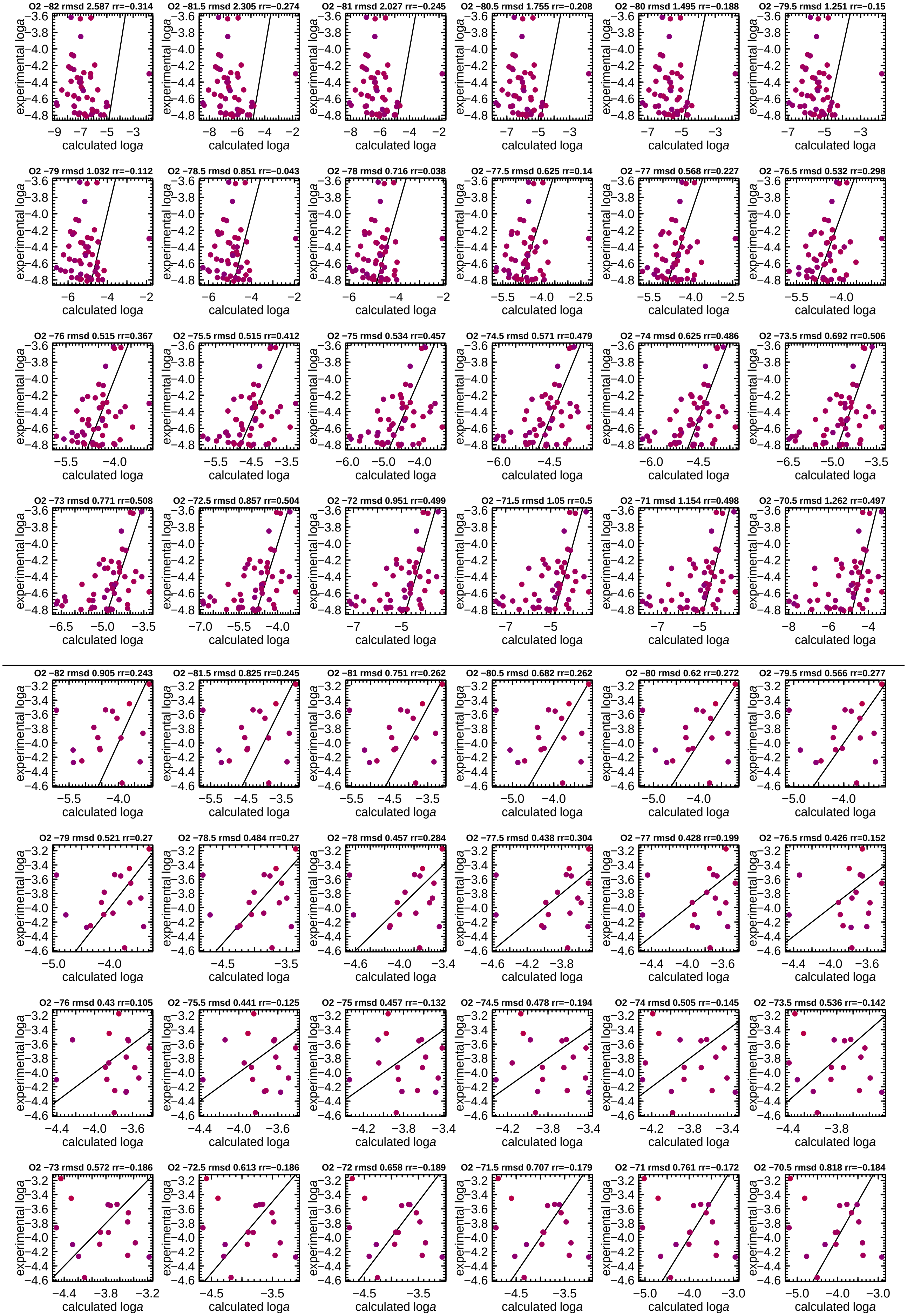

Figure S6-xv: mitochondrion (top); mitochondrial ribosome small subunit (bottom)

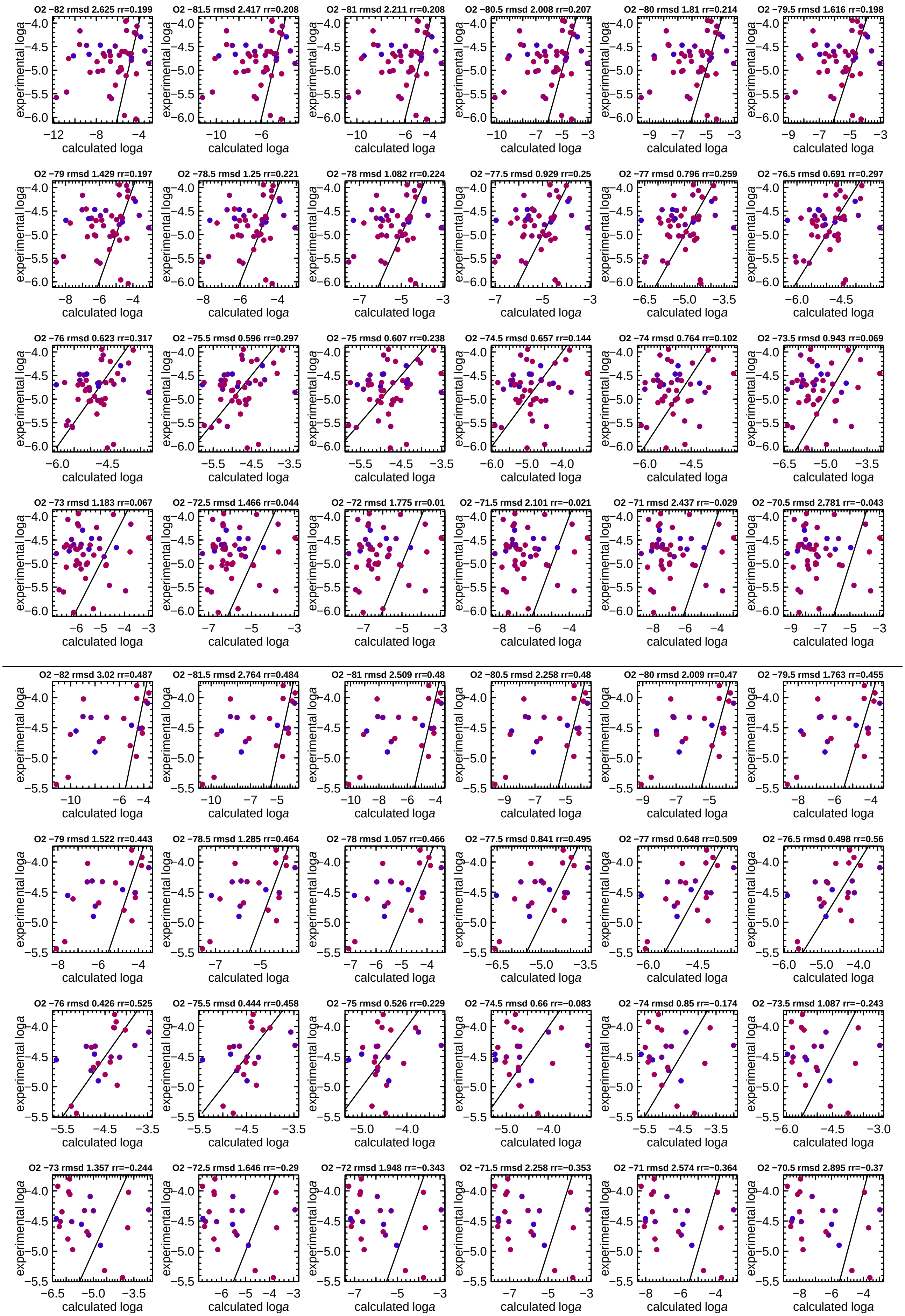

Figure S6-xvi: nuclear.periphery (top); nuclear pore complex (bottom)

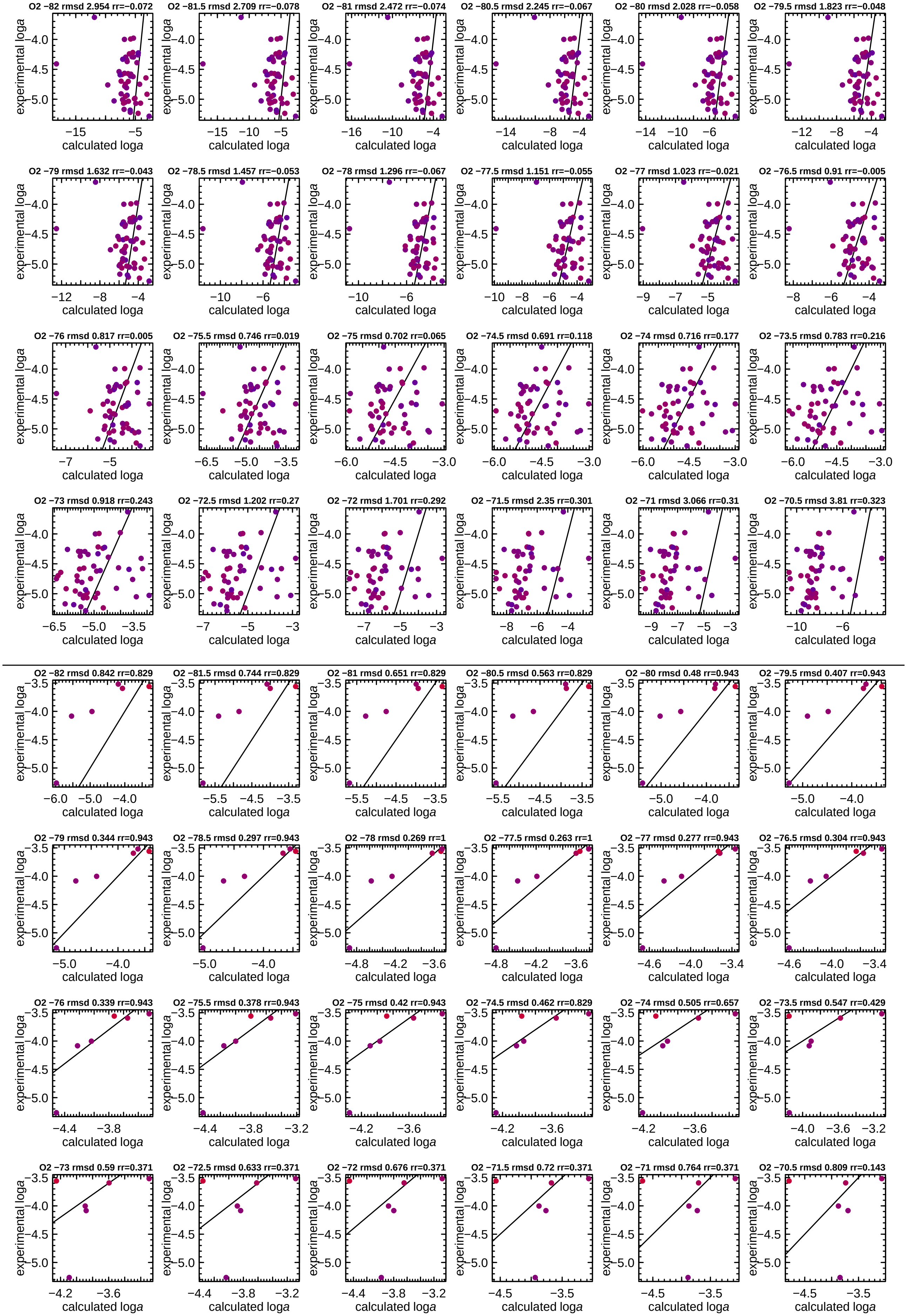

Figure S6-xvii: nucleolus (top); small subunit processome (bottom)

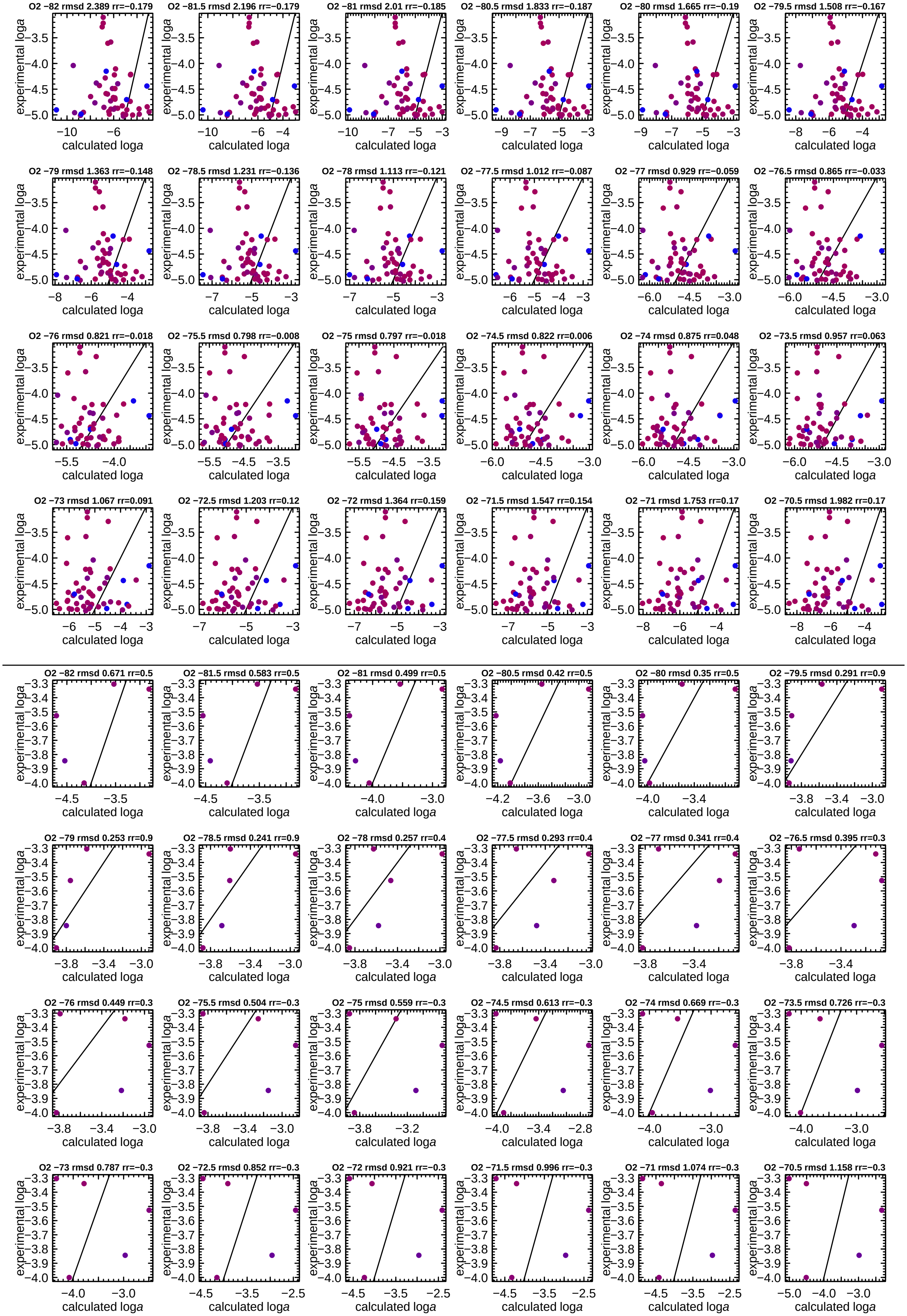

Figure S6-xviii: nucleus (top); RNA polymerase I (bottom)

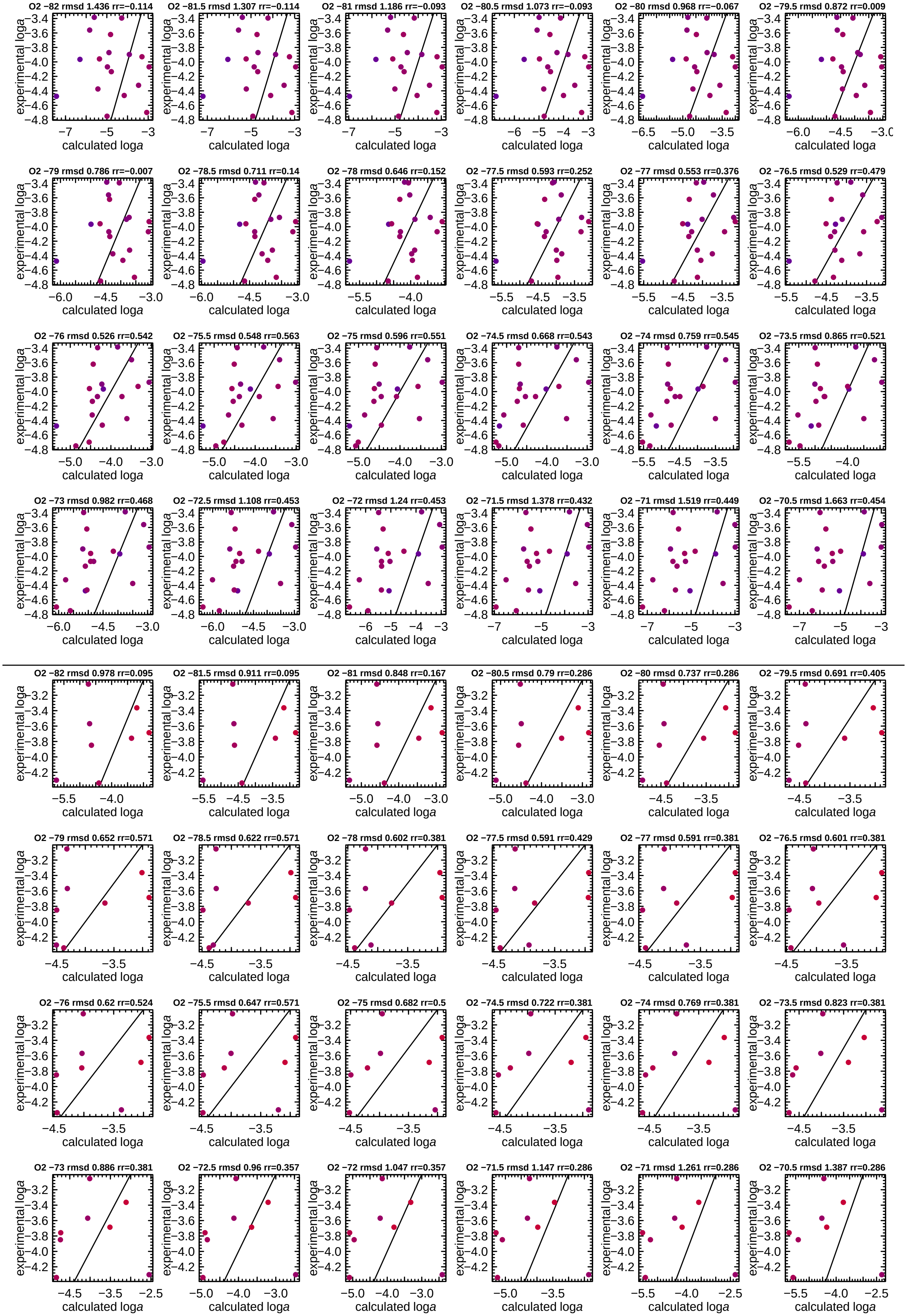

Figure S6-xix: peroxisome (top); integral to peroxisomal membrane (bottom)

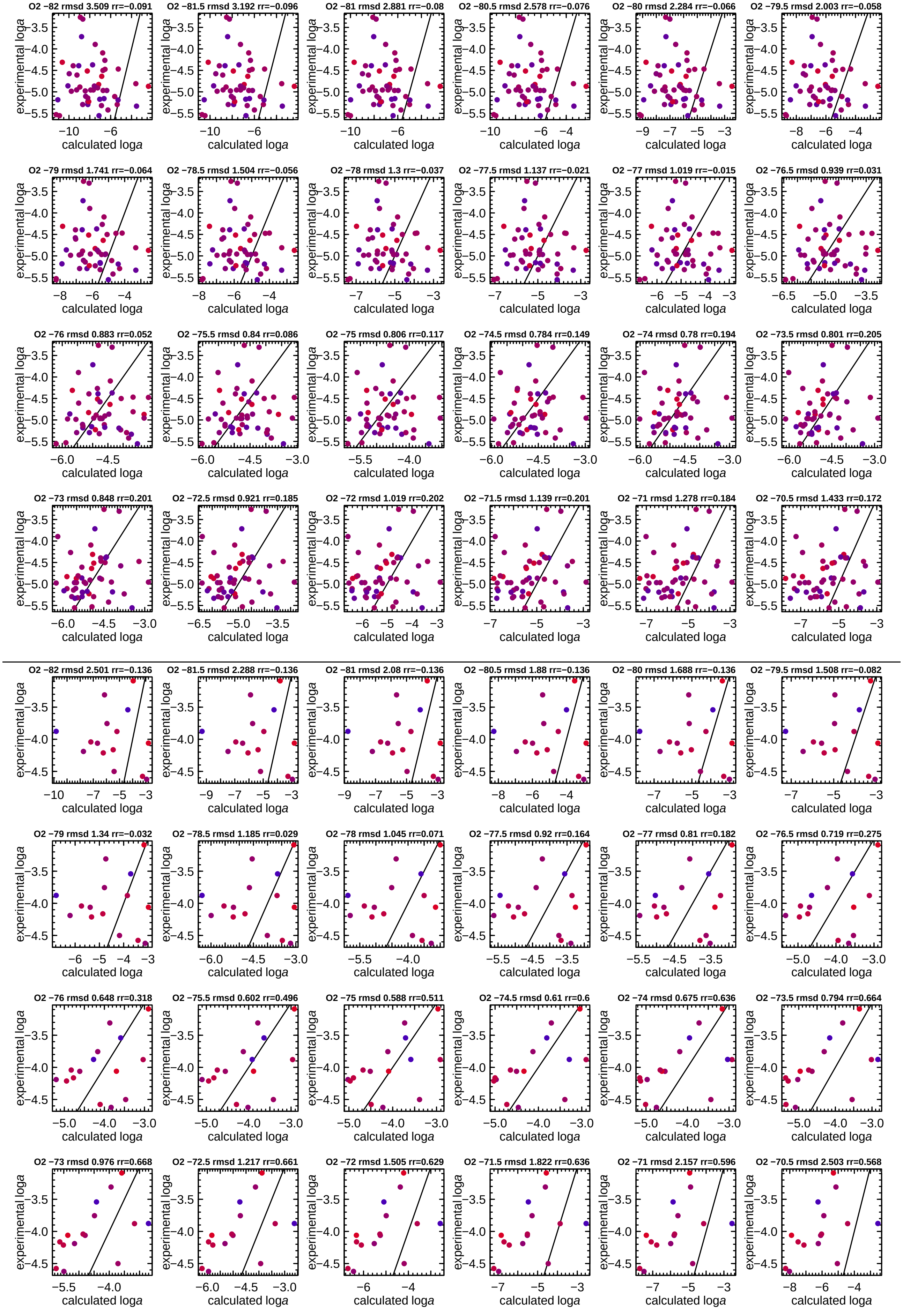

Figure S6-xx: punctate.composite (top); proteins localized here and early.Golgi (bottom)

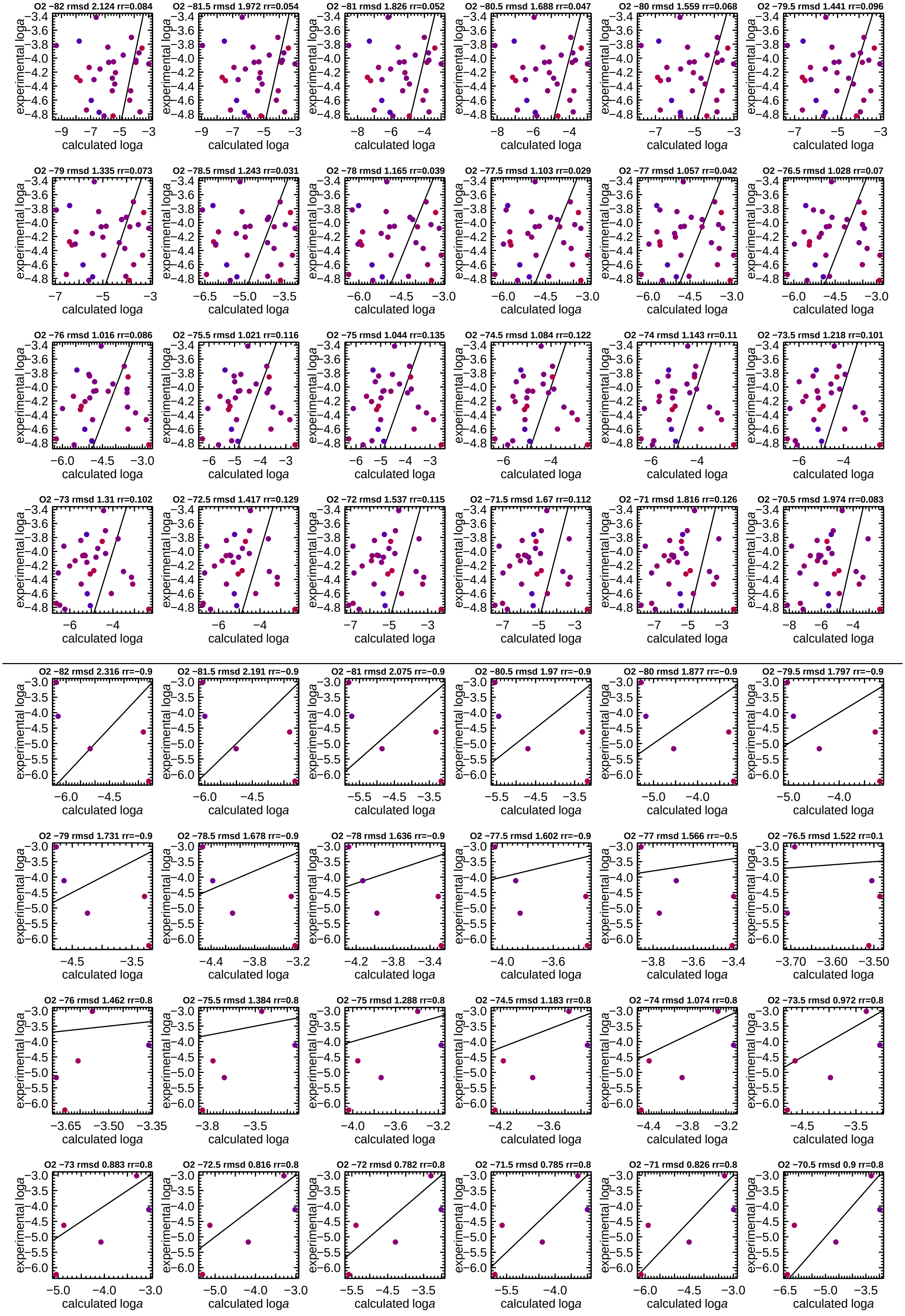

Figure S6-xxi: spindle.pole (top); spindle-pole body complex (bottom)

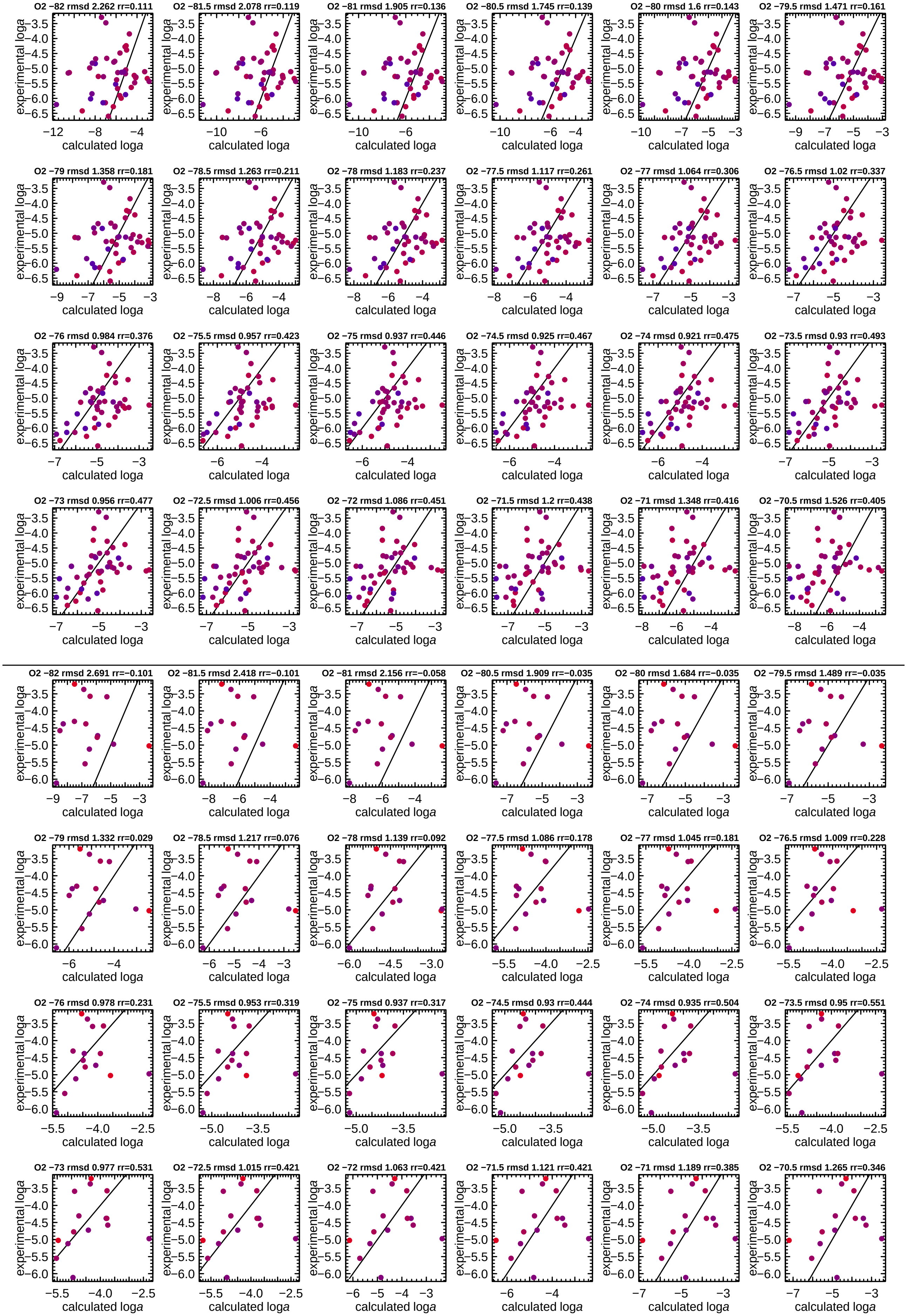

Figure S6-xxii: vacuolar.membrane (top); V0 vacuolar ATPase complex (bottom)

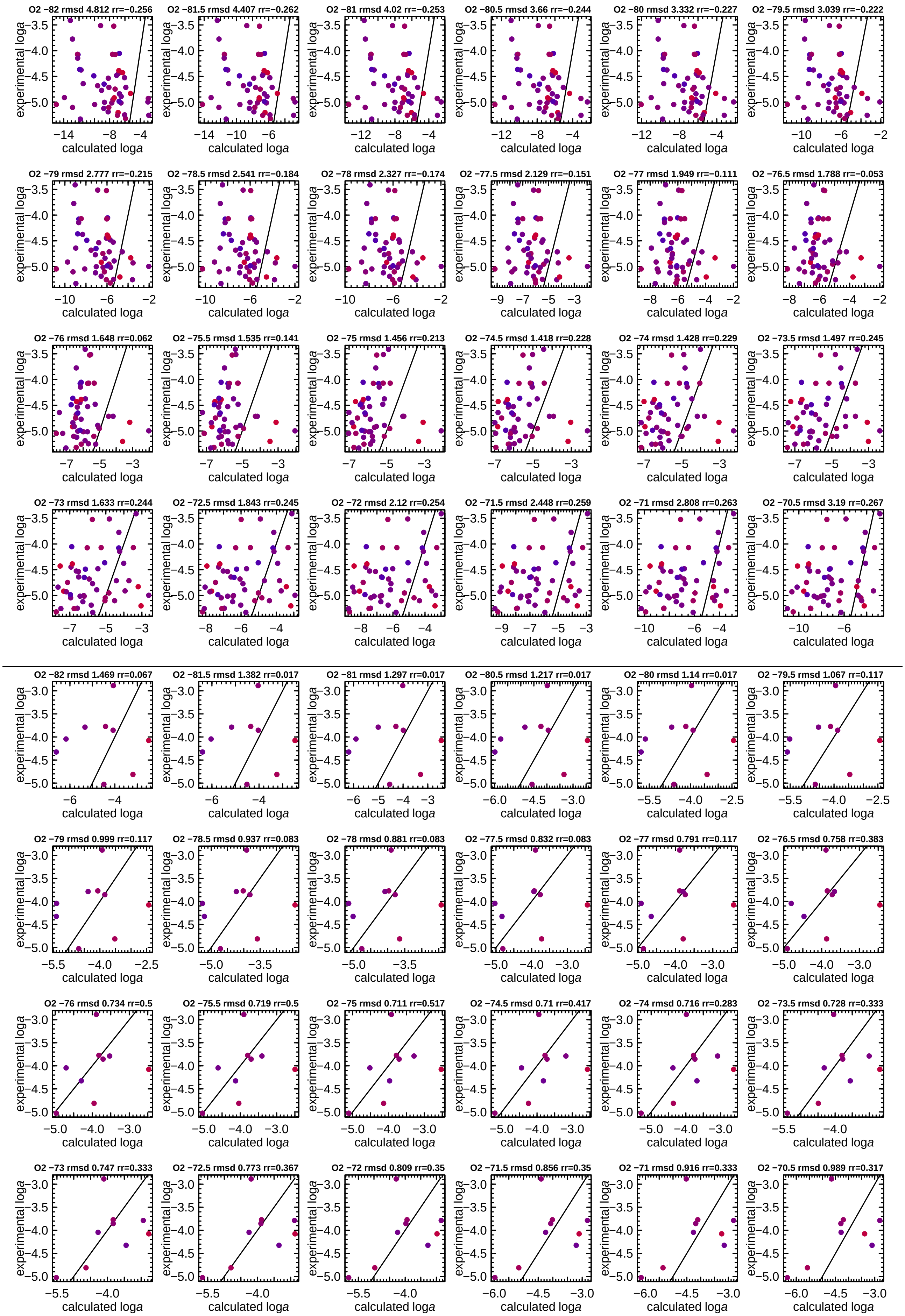

Figure S6-xxiii: vacuole (top); vacuolar proteases and other canonical proteins (bottom)
