# Supplementary material for: Calculation of the relative metastabilities of proteins in subcellular compartments of Saccharomyces cerevisiae
Source: BMC Syst Biol. 2009 Jul 18;3:75. doi: 10.1186/1752-0509-3-75 (PMC2734844; doi:10.1186/1752-0509-3-75)
Supplement: Additional file 7 — Identities of proteins in selected complexes. Lists the proteins in the selected model complexes and whether their abundances are reported in the YeastGFP dataset. Proteins without experimental abundance data were not used in the comparisons discussed in this study. [file 1752-0509-3-75-S7.pdf]

Additional File 7: Model proteins in complexes[a]

Name		ORF	
1. actin: Arp2/3 complex			
Arc15		YIL062C	
Arc18		YLR370C	
Arc19		YKL013C	
Arc35		YNR035C	
Arc40		YBR234C	NA
Arp2	*	YDL029W	
Arp3		YJR065C	
2. ambiguous: cyclin-dependent protein kinase complex			
Cdc28	*	YBR160W	
Cks1	*	YBR135W	
Cln2	*	YPL256C	
Cys4	*	YGR155W	
Sic1	*	YLR079W	
Clb3	*	YDL155W	
Cln1	*	YMR199W	
3. bud: actin-associated motor protein complex 2			
Myo2		YAL029C	
She4	*	YKL130C	
Mlc1		YBR130C	
Myo1		YGL106W	X
Cmd1	*	YKL007W	
Myo5	*	YIL034C	
4. bud.neck: septin complex			
Bud4		YJR092W	
Cdc10		YCR002C	
Cdc11		YJR076C	
Cdc12		YHR107C	
Cdc3		YLR314C	X
Shs1		YDL225W	
Mdh1	*	YKL085W	
5. cell.periphery: exocyst complex			
Exo84		YBR102C	NA
Sec10		YLR166C	
Sec3		YER008C	
Sec5		YDR166C	
Sec6		YIL068C	
Sec8		YPR055W	NA
6. cytoplasm: translation initiation factor eIF3			
Fun12		YAL035W	
Hcr1		YLR192C	
Nip1		YMR309C	
Prt1		YOR361C	
Rli1		YDR091C	
Rpg1		YBR079C	
Tif34		YMR146C	X
Tif35		YDR429C	NA
Tif5		YPR041W	
7. early.Golgi: SNARE complex			
Dsl1	*	YNL258C	
Sec39	*	YLR440C	
Tip20	*	YGL145W	
Ufe1	*	YOR075W	NA
Use1	*	YGL098W	
Pep12		YOR036W	X
Ykt6		YKL196C	X
8. endosome: ESCRT I & II complexes			
Vps23		YCL008C	X
Vps28	*	YPL065W	
Vps37		YLR119W	X
Mvb12		YGR206W	
Vps22	*	YPL002C	
Vps36	*	YLR417W	
Vps25		YJR102C	X

Name		ORF	
9. ER: signal recognition complex			
Sec65		YML105C	NA
Srp14		YDL092W	
Srp54		YPR088C	X
Spp68	*	YPL243W	
Srp72		YPL210C	
10. ER.to.Golgi: coatomer COPII complex			
Sec13		YLR208W	
Sec16		YPL085W	
Sec23		YPR181C	X
Sfb2		YNL049C	
Sec24		YIL109C	NA
Grh1	*	YDR517W	
11. Golgi: Golgi transport complex			
Cog1	*	YGL223C	
Cog2		YGR120C	
Cog3		YER157W	
Cog4	*	YPR105C	
Cog5		YNL051W	
Cog6		YNL041C	
Cog7		YGL005C	
Cog8	*	YML071C	
Iml1	*	YJR138W	
Nrp1	*	YDL167C	
12. late.Golgi: retrograde protein complex			
Kar2	*	YJL034W	
Vps52	*	YDR484W	
Vps53	*	YJL029C	NA
Vps54	*	YDR027C	
Vps51	*	YKR020W	
Scj1	*	YMR214W	
13. lipid.particle: sterol biosynthesis enzymes			
Erg9	*	YHR190W	
Erg1	*	YGR175C	
Erg7		YHR072W	
Erg11	*	YHR007C	
Erg24	*	YNL280C	
Erg25	*	YGR060W	
Erg26	*	YGL001C	
Erg27		YLR100W	NA
Erg6		YML008C	
Erg2	*	YMR202W	
Erg3	*	YLR056W	
Erg5	*	YMR015C	
Erg4	*	YGL012W	
15. mitochondrion: mitochondrial ribosome small subunit			
Ehd3		YDR036C	
Mrp13		YGR084C	
Mrp17		YKL003C	NA
Mrp21		YBL090W	
Mrp4		YHL004W	
Mrp51		YPL118W	
Mrps16		YPL013C	NA
Mrps17		YMR188C	
Mrps18		YNL306W	
Mrps28		YDR337W	
Mrps5		YBR251W	
Mrps8		YMR158W	X
Mrps9		YBR146W	X
Pet123		YOR158W	
Rsm10		YDR041W	
Rsm19		YNR037C	X
Rsm22		YKL155C	
Rsm23		YGL129C	
Rsm27		YGR215W	NA
Rsm7		YJR113C	
Mrp1		YDR347W	
Rsm25		YIL093C	
Nam9		YNL137C	

Name		ORF	
14. microtubule: DASH complex			
Dam1		YGR113W	X
Duo1	*	YGL061C	
Dad1	*	YDR016C	
Dad2	*	YKR083C	
Spc19	*	YDR201W	
Spc34	*	YKR037C	NA
Ask1	*	YKL052C	
Dad3	*	YBR233W-A	
Dad4	*	YDR320C-A	
Hsk3		YKL138C-A	X
16. nuclear.periphery: nuclear pore complex			
Nup60		YAR002W	
Nup170		YBL079W	
Asm4		YDL088C	
Nup84		YDL116W	
Gle1		YDL207W	
Nup42		YDR192C	X
Nup157		YER105C	
Gle2		YER107C	
Nic96		YFR002W	
Nup145		YGL092W	
Seh1		YGL100W	X
Nup49		YGL172W	
Nup57		YGR119C	
Nup159		YIL115C	
Nup192		YJL039C	
Nsp1		YJL041W	
Nup82		YJL061W	
Nup85		YJR042W	
Nup120		YKL057C	X
Nup100		YKL068W	
Nup133		YKR082W	
Pom34		YLR018C	NA
Ndc1		YML031W	
Nup188		YML103C	
Nup116	*	YMR047C	NA
Pom152		YMR129W	
Nup53		YMR153W	
Nup1		YOR098C	
Cdc31		YOR257W	X
17. nucleolus: small subunit processome			
Utp8		YGR128C	
Nan1		YPL126W	
Utp10		YJL109C	
Utp15		YMR093W	
Utp4		YDR324C	
Utp9		YHR196W	
18. nucleus: RNA polymerase I			
Rpa49	*	YNL248C	NA
Rpa12	*	YJR063W	
Rpa190	*	YOR341W	
RPApa3	*	YOR340C	
Rpc40		YPR110C	
Rpa135	*	YPR010C	
Rpb5		YBR154C	X
19. peroxisome: integral to peroxisomal membrane			
Ant1		YPR128C	
Inp2	*	YMR163C	
Pex12		YMR026C	
Pex15	*	YOL044W	
Pex22	*	YAL055W	
Pex3		YDR329C	
Pex30		YLR324W	
Pex31	*	YGR004W	
Pex32	*	YBR168W	NA
Pxa1		YPL147W	X
Pxa2		YKL188C	X

Name		ORF	
20. punctate.composite: proteins localized here and early.Golgi			
Arl1		YBR164C	
Apm3		YBR288C	
Bug1		YDL099W	
Arf1		YDL192W	
Luv1		YDR027C	
Tvp23		YDR084C	
Dop1		YDR141C	
Kei1		YDR367W	
Vrg4		YGL225W	
Apl6		YGR261C	
Aps3		YJL024C	
Vps53		YJL029C	NA
Tvp38		YKR088C	
Ssp120		YLR250W	
NA		YMR010W	
NA		YMR253C	NA
Kex2		YNL238W	NA
Mon2		YNL297C	
21. spindle.pole: spindle-pole body complex			
Pfk1	*	YGR240C	
Spc72		YAL047C	
Spc97		YHR172W	
Spc98		YNL126W	
Tub4		YLR212C	
22. vacuolar.membrane: VO vacuolar ATPase complex			
Emi2	*	YDR516C	
Vma6		YLR447C	
Vph2	*	YKL119C	
Bni1	*	YNL271C	
Drs2	*	YAL026C	
Gaa1	*	YLR088W	NA
Lys9	*	YNR050C	
Nop6	*	YDL213C	
Pdc1	*	YLR044C	
Pgi1	*	YBR196C	
Vac8		YEL013W	
Vma10		YHR039C-A	
Vma2		YBR127C	
Vma7		YGR020C	
Vph1		YOR270C	
Vtc4		YJL012C	X
Yor1	*	YGR281W	
Yra1	*	YDR381W	NA
23. vacuole: vacuolar proteases and other canonical proteins			
Ape1	*	YKL103C	
Ape3	*	YBR286W	
Lap3	*	YNL239W	
Pep4		YPL154C	NA
Prb1	*	YEL060C	
Prb1		YMR297W	
Ams1	*	YGL156W	
Ath1		YPR026W	X
Pho8		YDR481C	
Vtc4		YJL012C	X
Ypt7	*	YML001W	
Npc2		YDL046W	
NA		YHR202W	NA

a. Symbols: “*” the protein was not localized in the compartment (Huh et al., 2003); “X” or “NA” not tagged or no abundance (Ghaemmaghami et al., 2003).
