# Supplementary material for: Calculation of the relative metastabilities of proteins in subcellular compartments of Saccharomyces cerevisiae
Source: BMC Syst Biol. 2009 Jul 18;3:75. doi: 10.1186/1752-0509-3-75 (PMC2734844; doi:10.1186/1752-0509-3-75)
Supplement: Additional file 8 — Plots of relative abundances of model proteins for complexes. The calculated relative abundances of model proteins in selected complexes are shown as a function of log . [file 1752-0509-3-75-S8.pdf]

Additional File 8: Calculated logarithms of activities of model proteins in complexes

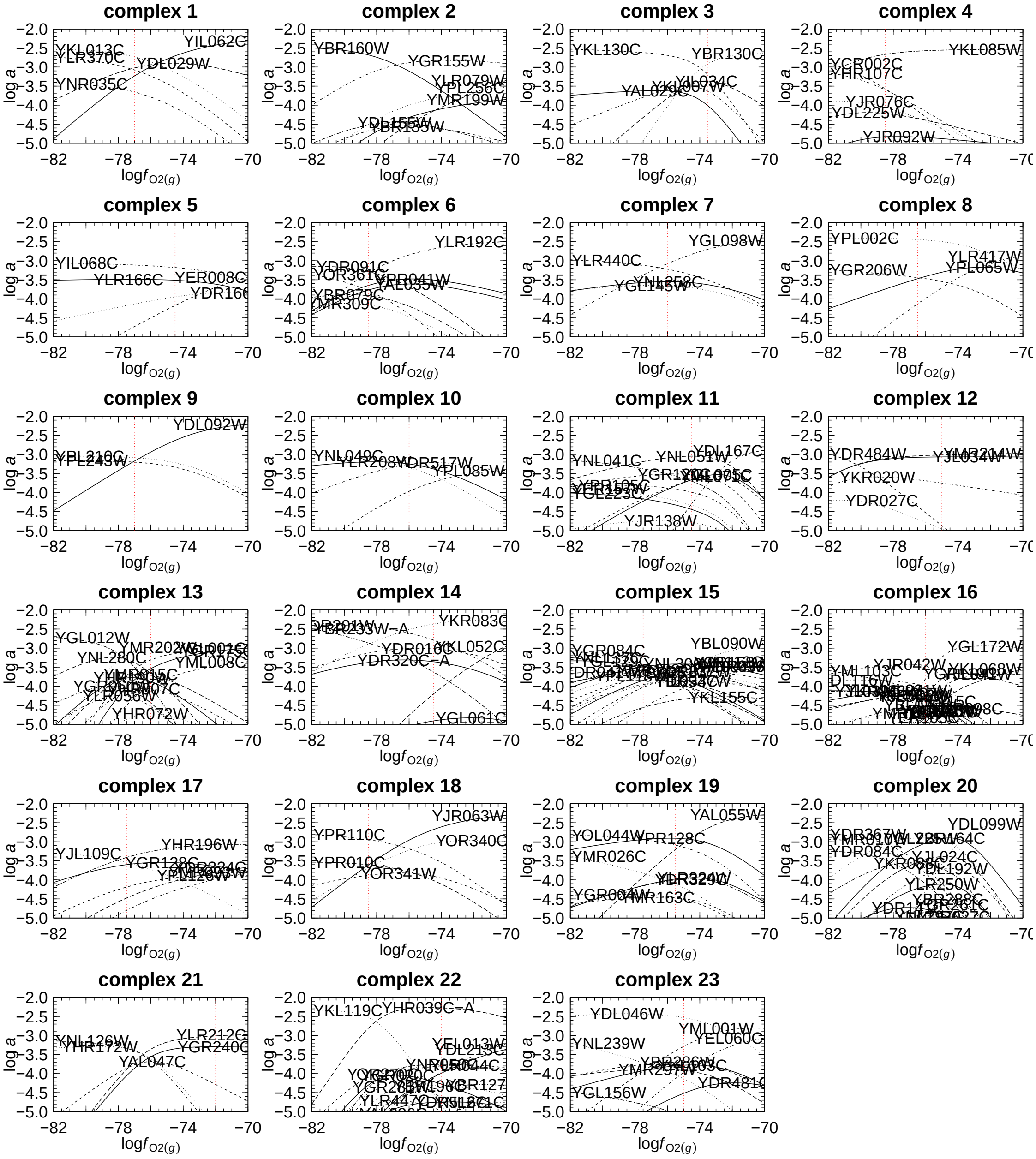

The numbered complexes are identified in Table 6 in the text and Additional File 8. Metastable equilibrium activities of proteins in the complexes were calculated as a function of log $f_{O_{2(g)}}$ for total activity of residues set to unity. Dotted red lines denote values of log $f_{O_{2(g)}}$ (listed in Table 5 in the text) and calculated relative abundances that were used in making Fig. 6 in the text.
