# Supplementary material for: Calculation of the relative metastabilities of proteins in subcellular compartments of Saccharomyces cerevisiae
Source: BMC Syst Biol. 2009 Jul 18;3:75. doi: 10.1186/1752-0509-3-75 (PMC2734844; doi:10.1186/1752-0509-3-75)
Supplement: Additional file 10 — Calculation of p-values for abundance rank correlations. This file lists calculated p-values for the Spearman rank correlation coefficients and describes the steps used in the calculations. [file 1752-0509-3-75-S10.pdf]

Additional File 10: Calculating the p-values for Spearman rank correlation

The p-value is (Wikipedia): "the probability of obtaining a result at least as extreme as the one that was actually observed, assuming that the null hypothesis is true" or (mathworld.wolfram.com): "the probability that a variate would assume a value greater than or equal to the observed value strictly by chance". For the following analysis, the null hypothesis can be stated as: the observed value of the Spearman rank correlation coefficient is obtained (or surpassed) by a completely random ordering of the proteins. A low enough p-value is considered to be sufficient evidence to reject this hypothesis.

The p-value does not measure the significance of the correlations, but it might be useful in deciding whether a correlation exists at all. The p-values for the Spearman correlation coefficient calculated below can also help one interpret the relative weights of each of the correlations, which involve different numbers of proteins.

The p-value can be calculated exactly when the size of the data set is small enough. For example, when $n = 3$, the number of permutations is 3!. Suppose we found a Spearman rank correlation coefficient of 1 (perfect positive rank correlation); an example is the outcome for the complex representing the ER compartment. There is only one permutation that leads to this outcome, so the p-value is $1/3! = 0.1667$. Traditionally, a p-value of 0.05 or lower is required to reject the null hypothesis. A perfect rank correlation among only 3 data points is not as convincing as a perfect rank correlation among 4 data points (e.g. the complex representing the ER to Golgi), which entails a p-value of $1/4! = 0.042$.

The exact calculation of the p-value is limited by available computational power, and in the present study was calculated for $n < 10$. Consider the case when $n = 10$, where the number of permutations is $10! = 3,628,800$ (e.g. complex 11 representing the Golgi compartment). In the present analysis up to $500,000$ random permutations of the calculated relative abundances of proteins were generated, and the Spearman rank correlation coefficient calculated between these and the observed relative abundances. If the correlation coefficient was equal to or greater than (for positive correlations only) or less than (for negative correlations only - i.e., the Golgi complex) that for the original set of abundances, the random sample was counted in the numerator of the p-value calculation. When done three times ($500,000$ random samples each), p-values of 0.45784, 0.45976 and 0.46116 were obtained in this case (second Table of this supplement).

This analysis supports the interpretation that there is a salient rank correlation between calculated and observed relative abundances of proteins in some of the cases studied here. Some of the strongest positive correlations are found in the model proteins from cell periphery, mitochondrion, and vacuolar membrane, and among the complexes, those selected to represent nucleolus, nuclear periphery and punctate composite. Among the negative rank correlations, only the most abundant proteins in Golgi, and the proteins in the selected complexes for cytoplasm and microtubule, are significant at a p-value cutoff of 0.05.

Table 1: Summary of Spearman rank correlation coefficients and p-values in comparisons of calculated and observed relative abundances of most abundant proteins in compartments. Abbreviations: n - number of data points; ρ - Spearman rank correlation coefficient; p.exact - exactly calculated p-value; p1–p3 - p-values calculated from random permutations; p.avg - average of p1–p3; factorial - factorial of n; n.samp - number of random permutations tested.

location	n	ρ	p.exact	p1	p2	p3	p.avg	factorial	n.samp
actin	22	0.191	NA	0.196	0.196	0.196	0.196	1.12E+21	500000
ambiguous	50	0.419	NA	0.001	0.001	0.001	0.001	3.04E+64	500000
bud	50	-0.015	NA	0.459	0.459	0.46	0.459	3.04E+64	500000
bud.neck	11	0.018	NA	0.485	0.483	0.484	0.484	39916800	500000
cell.periphery	38	0.417	NA	0.005	0.005	0.005	0.005	5.23E+44	500000
cytoplasm	50	0.188	NA	0.096	0.095	0.096	0.095	3.04E+64	500000
early.Golgi	9	0.45	0.115	0.115	0.115	0.114	0.115	362880	50000
endosome	30	0.277	NA	0.069	0.069	0.069	0.069	2.65E+32	500000
ER	49	0.033	NA	0.412	0.412	0.412	0.412	6.08E+62	500000
ER.to.Golgi	5	0.4	0.258	0.235	0.258	0.26	0.251	120	1000
Golgi	14	-0.543	NA	0.024	0.024	0.024	0.024	87178291200	500000
late.Golgi	29	0.166	NA	0.195	0.194	0.193	0.194	8.84E+30	500000
lipid.particle	17	0.227	NA	0.19	0.189	0.19	0.19	355687428096000	500000
microtubule	10	0.358	NA	0.157	0.157	0.157	0.157	3628800	500000
mitochondrion	50	0.457	NA	0	0	0.001	0	3.04E+64	500000
nuclear.periphery	46	0.317	NA	0.016	0.016	0.016	0.016	5.50E+57	500000
nucleolus	50	0.177	NA	0.109	0.108	0.11	0.109	3.04E+64	500000
nucleus	50	-0.018	NA	0.45	0.451	0.451	0.451	3.04E+64	500000
peroxisome	18	0.563	NA	0.008	0.008	0.008	0.008	6.40E+15	500000
punctate.composite	49	0.194	NA	0.09	0.091	0.091	0.09	6.08E+62	500000
spindle.pole	30	0.116	NA	0.27	0.271	0.27	0.27	2.65E+32	500000
vacuolar.membrane	45	0.475	NA	0.001	0.001	0.001	0.001	1.19E+56	500000
vacuole	50	0.228	NA	0.056	0.056	0.056	0.056	3.04E+64	500000

Table 2: Summary of Spearman rank correlation coefficients and p-values in comparisons of calculated and observed relative abundances of proteins in complexes. Abbreviations: n - number of data points; ρ - Spearman rank correlation coefficient; p.exact - exactly calculated p-value; p1–p3 - p-values calculated from random permutations; p.avg - average of p1–p3; factorial - factorial of n; n.samp - number of random permutations tested.

location	n	ρ	p.exact	p1	p2	p3	p.avg	factorial	n.samp
actin	5	-0.1	0.475	0.472	0.465	0.485	0.474	120	1000
ambiguous	7	0.5	0.133	0.133	0.144	0.123	0.133	5040	5000
bud	5	-0.3	0.342	0.348	0.297	0.32	0.322	120	1000
bud.neck	6	0.657	0.088	0.087	0.098	0.075	0.087	720	1000
cell.periphery	4	0.2	0.458	0.446	0.446	0.46	0.451	24	1000
cytoplasm	7	-0.821	0.017	0.016	0.017	0.019	0.017	5040	5000
early.Golgi	4	-0.8	0.167	0.166	0.166	0.178	0.17	24	1000
endosome	4	-0.8	0.167	0.161	0.159	0.151	0.157	24	1000
ER	3	1	0.167	0.166	0.173	0.187	0.175	6	1000
ER.to.Golgi	4	1	0.042	0.035	0.048	0.054	0.046	24	1000
Golgi	10	-0.042	NA	0.458	0.46	0.461	0.46	3628800	50000
late.Golgi	5	0.6	0.175	0.171	0.17	0.181	0.174	120	1000
lipid.particle	12	-0.189	NA	0.276	0.277	0.275	0.276	479001600	50000
microtubule	7	-0.75	0.033	0.033	0.036	0.036	0.035	5040	5000
mitochondrion	17	0.304	NA	0.117	0.117	0.119	0.118	3.55E+14	50000
nuclear.periphery	23	0.525	NA	0.005	0.005	0.006	0.006	2.58E+22	50000
nucleolus	6	1	0.001	0.001	0	0	0	720	1000
nucleus	5	0.9	0.042	0.047	0.029	0.052	0.043	120	1000
peroxisome	8	0.571	0.076	0.081	0.075	0.072	0.076	40320	5000
punctate.composite	15	0.636	NA	0.007	0.007	0.006	0.006	1307674368000	50000
spindle.pole	5	0.8	0.067	0.081	0.059	0.062	0.067	120	1000
vacuolar.membrane	15	0.504	NA	0.028	0.029	0.028	0.028	1307674368000	50000
vacuole	9	0.517	0.081	0.082	0.082	0.081	0.082	362880	50000
